# Biodiversity patterns diverge along geographic temperature gradients

**DOI:** 10.1111/gcb.16457

**Published:** 2022-11-16

**Authors:** Charlie J. G. Loewen, Donald A. Jackson, Benjamin Gilbert

**Affiliations:** ^1^ Department of Ecology and Evolutionary Biology University of Toronto Toronto Ontario Canada; ^2^ Department of Ecology, Evolution, and Organismal Biology Iowa State University Ames Iowa USA

**Keywords:** body size, conservation biogeography, elevational diversity gradient, environmental filtering, functional diversity, latitudinal diversity gradient, mountain lakes, phylogenetic diversity

## Abstract

Models applying space‐for‐time substitution, including those projecting ecological responses to climate change, generally assume an elevational and latitudinal equivalence that is rarely tested. However, a mismatch may lead to different capacities for providing climatic refuge to dispersing species. We compiled community data on zooplankton, ectothermic animals that form the consumer basis of most aquatic food webs, from over 1200 mountain lakes and ponds across western North America to assess biodiversity along geographic temperature gradients spanning nearly 3750 m elevation and 30° latitude. Species richness, phylogenetic relationships, and functional diversity all showed contrasting responses across gradients, with richness metrics plateauing at low elevations but exhibiting intermediate latitudinal maxima. The nonmonotonic/hump‐shaped diversity trends with latitude emerged from geographic interactions, including weaker latitudinal relationships at higher elevations (i.e. in alpine lakes) linked to different underlying drivers. Here, divergent patterns of phylogenetic and functional trait dispersion indicate shifting roles of environmental filters and limiting similarity in the assembly of communities with increasing elevation and latitude. We further tested whether gradients showed common responses to warmer temperatures and found that mean annual (but not seasonal) temperatures predicted elevational richness patterns but failed to capture consistent trends with latitude, meaning that predictions of how climate change will influence diversity also differ between gradients. Contrasting responses to elevation‐ and latitude‐driven warming suggest different limits on climatic refugia and likely greater barriers to northward range expansion.

## Graphical abstract

Integrating historical lake sampling from a series of adjacent mountain ranges in western North America (a) we assessed variation across multiple dimensions of zooplankton biodiversity both within, and between, elevational and latitudinal gradients (b). We discovered contrasting patterns linked to geographic interactions, including weaker latitudinal relationships in high altitude, alpine lakes (c). Diversity trends diverged when contrasting elevational and latitudinal patterns, as did responses to mean temperatures and seasonality. These differences suggest that elevation and latitude have limited equivalence in their capacities to provide climate‐change refuge to migrating species.

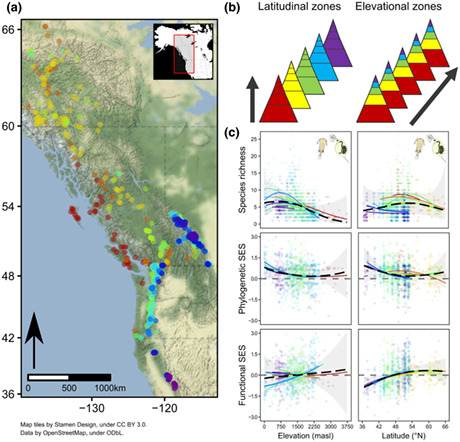

## 1 INTRODUCTION

Geographic patterns of biological variation emerge across multiple taxa, including zooplankton (Pinel‐Alloul et al., 2013), fish (Brucet et al., 2013), stream bacteria and diatoms (Wang et al., 2017), soil bacteria (Bryant et al., 2008), mammals (McCain, 2005), plants (Alahuhta et al., 2020), birds (Terborgh, 1977), and others. A common observation is that species richness declines with increasing elevation or latitude (Hillebrand, 2004; Rahbek, 1995). However, gradients may take different forms (from monotonic to hump‐shaped; Hof et al., 2008; Montaño‐Centellas et al., 2020; Peters et al., 2016) and the nature of relationships across multiple dimensions of biodiversity is relatively unexplored (Kirk et al., 2022; Kohli et al., 2021).

While interchangeability of elevation and latitude is often implicit in space‐for‐time substitution and assessment of ecological risk from climate change (Davis & Shaw, 2001; Meerhoff et al., 2012; Pauchard et al., 2016), geographic gradients are usually studied separately, limiting understanding of how they co‐influence communities across broader scales. This lack of integration likely stems from a deficiency of datasets capturing both gradients with adequate scope and resolution. Research restricted to one or a few elevational transects cannot reveal latitudinal differences (e.g. Peters et al., 2016; Terborgh, 1977) and latitudinal studies frequently overlook elevation or lack sufficient range to permit useful comparisons (e.g. Alahuhta et al., 2020; Hillebrand, 2004). Global syntheses have been conducted to overcome single‐study limitations and test for geographic variations (e.g. Guo et al., 2013; Jarzyna et al., 2021; Montaño‐Centellas et al., 2020; Picazo et al., 2020), but these studies have their own challenges. For instance, the diversity of terrestrial and marine taxa is often estimated from species' range maps (e.g. McCain, 2005; Ready et al., 2010), where communities are compiled as grid cells of overlapping distributions (and biased by widely dispersed species; Quintero & Jetz, 2018) rather than actual observations of co‐occurring species. As lake communities are comparatively well‐defined and spatially replicated, with clear boundaries and common sampling protocols allowing lake‐level inferences, they are ideal model systems for testing how diversity varies both within, and between, foundational geographic and climatic gradients.

Most freshwater taxa are ectothermic, meaning that their distributions and community diversity are expected to be constrained by temperature (e.g. Atkinson, 1994; Buckley et al., 2012), but it is unclear whether temperature restricts ectotherm richness by limiting productivity (energy‐richness hypothesis), imposing seasonality (breadth of physiological tolerance), or setting the rate of speciation (Currie et al., 2004). Speciation also depends on dispersal, as barriers to movement facilitate diversification and development of continental diversity patterns (e.g. Claramunt et al., 2012). While the relatively strong dispersal capacity of plankton and other passively dispersed microorganisms may limit reproductive isolation (Whitaker, 2006), colonization is contingent upon climatic suitability and few studies have compared the importance of energy availability (as related to mean temperatures) to range of thermal environments that impact species through tolerance limits (seasonal temperature difference; but see Picazo et al., 2020). Understanding how different facets of thermal regimes influence communities is critical for predicting the consequences of global change as properties such as mean temperature and its variance may shift independently yet exert joint impacts (e.g. Easterling et al., 2000; Vasseur et al., 2014) to which geographic gradients may offer different refuge for migrating species.

While functional and phylogenetic diversity of communities are closely tied to species richness (Mason et al., 2013; Mazel et al., 2016), environmental filtering and biotic interactions vary with species' traits and thus the functional characteristics of communities can provide insight into how communities are assembled. For example, assemblages with similar traits for tolerance to warm, cold, or seasonally varying temperatures (i.e. functional clustering or underdispersion) may indicate strong climate drivers. In contrast, species might be more phenotypically distinct (i.e. functionally overdispersed) in richer, more productive communities where potential for strong competition selects for species with reduced niche sizes and overlap (Lesser et al., 2020; Pigot et al., 2016). However, competition (as well as facilitation) can also lead to functional clustering along specific trait axes, such as those permitting access to limiting resources (Mayfield & Levine, 2010). Therefore, functional dispersion patterns often differ between individual traits, which tend to correlate with fitness differences among species (determining competitive dominance), and combinations of traits, which can foster stabilizing differences that facilitate species coexistence (i.e. niche differentiation; Kraft et al., 2015). In lieu of comprehensive trait data, phylogenies (or their taxonomic surrogates) can be useful proxies for multiple, unmeasured traits if niches are sufficiently conserved within lineages (Tucker et al., 2018; Winter et al., 2013). Convergent evolution and character displacement complicate phylogenetic interpretations, and thus traits are preferable for linking ecological processes to functional differences among species (Münkemüller et al., 2020). Nonetheless, phylogenetic diversity is frequently linked to ecosystem functioning (e.g. Flynn et al., 2011) and can reveal valuable patterns for conservation planning (Faith, 2013).

Leveraging a unique limnological dataset spanning broad geographic gradients across a series of adjacent mountain ranges in western North America, we assessed variation across multiple dimensions of zooplankton biodiversity to test a series of ecological hypotheses. Our first objective was to examine the congruence of different metrics and evaluate their trends across elevational and latitudinal gradients to obtain mechanistic insights into community assembly. We expected that diversity would generally decline with increasing elevation and latitude, though the shape of these relationships (e.g. exhibiting low plateaus or mid‐gradient peaks) was uncertain, and we anticipated potentially different trends (e.g. steeper slopes along elevational gradients) due to differences in gradient length, rate of change, or underlying processes (Hypothesis H1a). We predicted greater taxonomic than functional or phylogenetic differences with both elevation and latitude, reflecting greater trait redundancies in species‐rich regions of each gradient (H1b). We also anticipated that filtering by harsh environmental conditions in high‐elevation and ‐latitude regions would cause functional and phylogenetic underdispersion (i.e. greater relatedness or clustering), where only hardy species can persist (e.g. Kirk et al., 2022; Wang et al., 2012). Specifically, we expected larger body sizes in colder, northern and alpine lakes due to reduced developmental rates and selection for species with greater size at maturity (H1c; Atkinson, 1994). In contrast, we predicted longer food chains with a greater diversity of predators in more productive systems at lower latitudes and elevations (H1d; Dodds et al., 2019). Although species interactions can lead to clustering of certain key traits (e.g. mediating resource limitation or prey susceptibility; Mayfield & Levine, 2010), they can also drive overdispersion by limiting similarity across multiple trait dimensions (reflecting greater specialization; Kraft et al., 2015). Therefore, we hypothesized that communities in more productive and environmentally stable southern and montane lakes would exhibit relatively greater functional and phylogenetic dispersion indicative of niche differentiation permitting the coexistence of more species (H1e).

Our second objective was to explore latitudinal differences along elevational gradients and elevational differences along latitudinal gradients, hypothesizing weaker relationships (i.e. flatter slopes and shifted intercepts) in alpine and northern lakes indicative of an antagonistic interaction whereby geographic gradients have less influence at their extremes (H2). Our third objective was to investigate the sensitivity of zooplankton diversity to thermal conditions. Here, we hypothesized diversity patterns would be more associated with seasonal than mean temperatures (H3), reflecting the notion that ‘mountain passes are higher’ nearer the equator (Janzen, 1967) where reduced climatic variation selects for narrower thermal tolerances and stronger stratification of communities (Polato et al., 2018; Rahbek et al., 2019). Species with restricted physiological tolerances should experience stronger dispersal limitation, encouraging biological diversification and the establishment of diversity gradients.

## 2 MATERIALS AND METHODS

### 2.1 Study system

**FIGURE 1:**
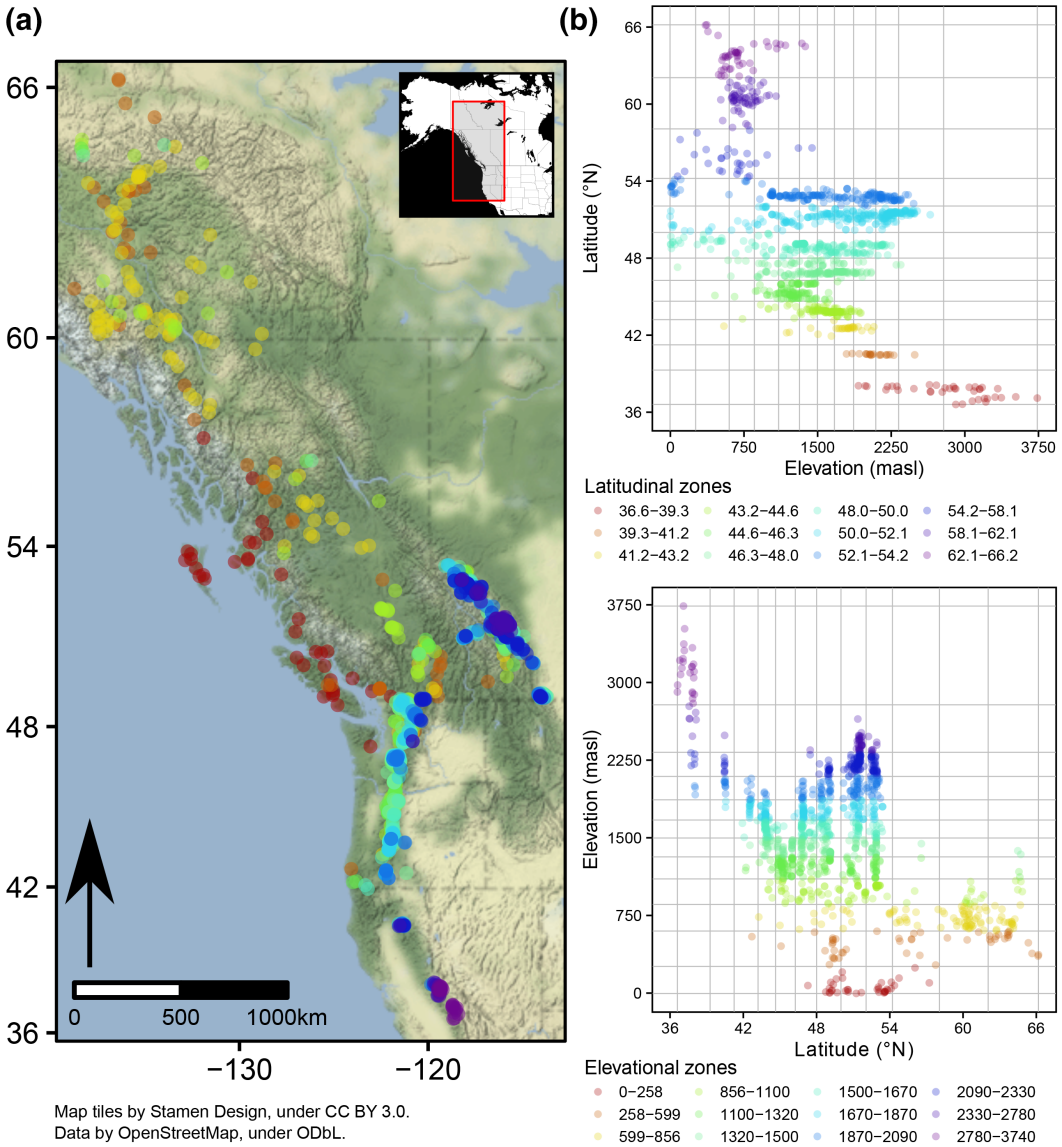
Sampling locations across mountain regions of western North American (a) and their discretization into elevational and latitudinal zones (b). The color of sampling locations in panel (a) reflect elevational zones. Map lines delineate study areas and do not necessarily depict accepted national boundaries.

We assessed geographic gradients of crustacean zooplankton biodiversity using historical sampling records for 1241 lakes and ponds (herein referred to as lakes) in mountainous regions of western Canada and the USA (Figure 1). Most sampling locations (1069; 86%) would be classified as lakes (as opposed to ponds) based on their size (greater than 5 ha) or depth (greater than 5 m; Richardson et al., 2022). Lakes ranged from ~0 to 3740 m above sea level (m a.s.l.) and 36.6°–66.2° latitude (spanning over 3200 km) across large swaths of the North American Cordillera in the Yukon, British Columbia, Alberta, Washington, Oregon, and California (Loewen, 2022).

Records of zooplankton occurrence over a 52‐year period (1964–2015) were compiled from multiple sources, including published articles, technical reports, and government datasets (previously described by Loewen et al., 2019). Lakes were generally sampled between May and September by pulling conical nets (63–100‐μm mesh sizes) through the water column, either vertically or horizontally, and collected organisms were enumerated using stereomicroscopy. As many records were either aggregated across multiple events or presented with uncertain collection dates, we gathered cumulative species lists for each lake permitting varied sampling protocols in favor of discounting zooplankton known to be present. However, we excluded several nontarget benthic invertebrates (of class Ostracoda, order Harpacticoida, and suborder Laevicaudata) and large, fast‐moving shrimps (of orders Amphipoda, Anostraca, Notostraca, and Mysida) that were known to be undersampled. Data were standardized to reflect current taxonomy as per appendix S1 of Loewen et al. (2019), and 119 taxa (identified to genus or higher) were retained for analysis.

### 2.2 Biodiversity metrics

We calculated metrics representing major taxonomic, functional, and phylogenetic dimensions of local (alpha) diversity at each sampling location. Taxonomic (species) richness was the only metric for which juveniles and other taxa identified below genus were used, adding to counts when no corresponding adults were found. Functional diversity was assessed using species body lengths and feeding strategies. Trait values were used to generate a species‐species distance matrix, based on Gower dissimilarities (Gower, 1971) providing equal weighting to each of the two traits, and define a functional ordination space for richness and dispersion metrics. Body lengths were obtained from the literature, prioritizing measurements taken within the study area where available, and treated as a continuous variable (Table S1). Feeding strategies were defined by species' morphological traits reflecting their behaviour as consumers and assigned ordinally (using the ranked ‘metric’ approach; Podani, 1999) to reflect a rough trophic gradient with increasing carnivory, from (1) substrate‐grazing, to (2) seston‐filtering, (3) stationary suspension‐feeding with occasional grasping, and (4) raptorial‐feeding (Loewen et al., 2020; Mimouni et al., 2018). Trait values were used to estimate functional richness and dispersion, as well as community‐weighted means reflecting the average size and feeding guild/trophic role of species in each community.

As trait‐based richness and dispersion metrics may be sensitive to the number of species present in a community, we also calculated species richness‐controlled measures reflecting the similarity of species traits compared to random communities (Mason et al., 2013). While the use of null models may not always eliminate richness‐dependence, standardized richness and dispersion metrics provide a useful estimate of relative trait clustering (Qian et al., 2020). However, because differences between observed measures and the mean of random communities may be biased when the distribution of null values is asymmetric, we calculated standardized effect sizes (SES) as probit‐transformed quantile *p*‐values (following Lhotsky et al., 2016; see Appendix S1 for details). Functional metrics were calculated using *dbFD* and related functions in the ‘FD’ package (Laliberté & Legendre, 2010).

Phylogenetic diversity was assessed based on the branching structure of a tree constructed from taxonomic ranks as a proxy for species relatedness. As a complete, time‐calibrated phylogeny was not available, Linnaean nomenclature (species through class) was used as input for the *class2tree* function in the ‘taxize’ package (Chamberlain & Szöcs, 2013) to represent phylogenetic relationships among species in the regional pool (Figure S1). We estimated Faith's index to sum branches and mean pairwise distance to assess average relatedness for subsets of the regional tree corresponding to each local community with at least two constituent species (using the *pd* and *mpd* functions from the ‘picante’ package; Kembel et al., 2010). We also calculated standardized measures of richness (known as the phylogenetic diversity index) and mean pairwise distance (known as the net relatedness index) to evaluate complementary ‘terminal’ and ‘basal’ dimensions of phylogenetic structure (Mazel et al., 2016), comparing observed values to those of zonal null communities as described in Appendix S1 (using the *ses.pd* and *ses.mpd* functions; Kembel et al., 2010). For all SES, higher values indicated relative excess of diversity (overdispersion) and lower values indicated clustering (underdispersion). As raw functional dispersion and phylogenetic mean pairwise distance showed similar patterns to their standardized counterparts (i.e. were not strongly related to species richness), we present only the latter.

### 2.3 Statistical analysis

A challenge in testing both elevational and latitudinal trends in diversity is that any interactions between the two may create complex patterns that can be difficult to interpret. We used an approach in which we isolated geographic relationships while simultaneously identifying their interactive effects. First, we divided our data into (1) transects that were within a narrow elevational range (mean = 312 m a.s.l.) but varied in latitude (‘elevational zones’ to test latitude trends), and (2) transects that were within a narrow latitudinal range (mean = 2.5°) but varied in elevation (‘latitudinal zones’ to test elevation trends; Figure 1). Zonal boundaries were defined as natural breaks in the distribution of sampling locations, with classes determined using the Fisher algorithm (*classIntervals* function with Sturges' formula; ‘classInt’ package; Bivand, 2020) constrained such that both elevation and latitude had the same number of zones (12). While there are many alternatives for defining class intervals, the Fisher algorithm performed well at identifying breaks between adjacent ranges and Sturge's formula offered a common method to determining the optimal number of transects given the number of observations.

We then evaluated geographical relationships to biodiversity using generalized linear mixed effect/multilevel modelling. This approach implicitly assumed greater similarity of samples collected in closer latitudinal or elevational proximity but offered flexibility in how trends could vary and accounted for differences in variation among groups. Separate models were developed for each biodiversity metric (see Table S2 for error distribution and link specifications), with fixed effects corresponding to first‐ and second‐order orthogonal polynomials of either elevation or latitude (obtained using the *poly* function; R Core Team, 2021). Orthogonal quadratic terms were included to capture anticipated nonlinearities, such as plateaus and mid‐gradient peaks (e.g. Montaño‐Centellas et al., 2020), while avoiding predictor collinearity. Random intercepts and slope coefficients were estimated for latitudinal zones in elevation models, and elevational zones in latitude models, providing information on the degree of random variation across zones (termed ‘group‐level’ effects).

With partial pooling of information across groups (shrinking parameter estimates towards the mean) and the propagation of uncertainty to population‐level estimates, multilevel models were used to obtain robust trend estimates given our unbalanced design (groups with different numbers of observations) and differences in elevational and latitudinal ranges among zones with potential disparities in sampling protocols. By estimating latitudinal trends at different elevations (and elevational trends at different latitudes) we were also able to explore a wide range of potential nonlinear responses. In contrast to traditional interaction models, where the multiplicative combination of variables is assumed to change at a constant rate (estimated by its slope coefficient), our approach permitted us to model more complex, nonmonotonic interactions involving mid‐gradient change points. While such relationships may be captured by multiplicative polynomial terms, their parameter estimates are difficult to interpret and lack other benefits of multilevel modelling. For instance, as elevational trends differed across mountain ranges (and latitudinal trends differed across elevational zones), random effects were useful to account for the nonindependence of observations within groups. We present parallel results of standard multiplicative interaction models for reference (biodiversity metrics regressed on latitude, elevation, and their interaction; Table S3; Figure S2); however, we limit interpretations to our primary analyses given the nonlinearity of observed patterns.

We evaluated climatic relationships to elevation, latitude, and the full suite of biodiversity metrics discussed above using the same modelling approach (only species richness shown in main text). Here, fixed effects corresponded to first‐ and second‐order orthogonal polynomials of either mean annual temperature or temperature difference between the means of the warmest and coldest months (i.e. seasonality). Climate variables were scale‐free point estimates averaged across the entire study period (1964–2015) obtained using ClimateNA v6.40 (Wang et al., 2016), which downscales gridded, monthly climate surfaces (30‐arcsec) interpolated from historical weather station measurements (Daly et al., 2008; Hijmans et al., 2005). Despite samples being collected over a 52‐year period, temporal shifts in climate were small compared to spatial differences. For example, while 10‐year normals for mean annual air temperature at sampling locations increased between 0.23 and 2.47°C (mean = 1.29°C) over this time, the average temperatures at the warmest and coolest lakes differed by 18.86°C (range = −7.86–11.00°C). Elevation, latitude, and climate predictors were centered and scaled prior to analysis (first subtracting the mean and then dividing by the standard deviation) to facilitate interpretation of interactions and derive comparable, unitless measures; however, raw values were used for plotting. As these variables were standardized as *z*‐scores, Gaussian models regressing climate against elevation and latitude were used to assess the strength and direction of their correlations (i.e. showing climatic relationships to geographic gradients).

All models were fit applying the Stan computational framework for Bayesian analysis (Stan Development Team; https://mc‐stan.org/) as implemented with the ‘brms’ package (Bürkner, 2017) using R 4.0.4 (R Core Team, 2021). This approach offered strong model convergence, flexible error distribution and link functions, and an intuitive, probabilistic means of evaluating parameter estimates accounting for uncertainty at both the group‐ and population‐levels. Four Markov chains were generated to sample posterior distributions for each model using the No‐U‐Turn Sampler (NUTS) run for 3000 iterations (plus 1000 warmup). NUTS is a variant of the highly efficient Hamiltonian Monte Carlo algorithm that avoids random walk behaviour and adaptively sets path lengths without hand‐tuning (Hoffman & Gelman, 2014). Analyses were applied with weakly informative, default priors, except population‐level coefficients (class ‘b’) were set to follow a normal distribution with mean = 0 and standard deviation = 5 (rather than default flat priors). While these priors were chosen to improve convergence by focusing on more plausible values, their influence on posterior distributions were generally minor given our large sample size (and thus principal importance of likelihood functions). Model checks indicated strong convergence (except for functional richness, which was bimodally distributed and estimated by a mixture model with different priors for population‐level intercepts; see Appendix S2 for details) and generally good fit to observed data (Figures S3–S60).

Support for model parameters were assessed based on the full posterior probability distribution, where 95% credible intervals (CI) excluding zero provided strong evidence that relationships (such as slope or curvature) were either positive or negative. Median group‐ and population‐level predictions for plotting were obtained from posterior draws of the linear predictor using the *ggpredict* function in the ‘ggeffects’ package (Lüdecke, 2018). To assess importance of taking group differences into account, we fit additional models without random effects (and without second‐order polynomials). Models were compared based on their widely applicable (WAIC) and leave‐one‐out cross‐validation information criteria (LOOIC), with better scores (lower values) when random effects were included signifying meaningful differences among lakes from different zones, such as differences in mean richness or rate of change (Table S4). Similarly, better scores with polynomial terms indicated models benefiting from the consideration of quadratic trends. Plots for fixed‐effect models of species richness (naïve of group‐level effects) are presented in Figure S61.

Finally, we used a similar approach to assess the robustness of our findings to variations in habitat size and sampling effort. For habitat size, we tested the effects of area and depth on species richness and their potentially confounding influence on conditional elevational and latitudinal patterns when treated as covariates. We also examined relationships between habitat size and geographic variables. For sampling effort, while data limitations prevented us from accounting for differences in the number of counted individuals through rarefaction, we tested the importance of number of sampling events conducted and number of years sampled.

## 3 RESULTS

**FIGURE 2:**
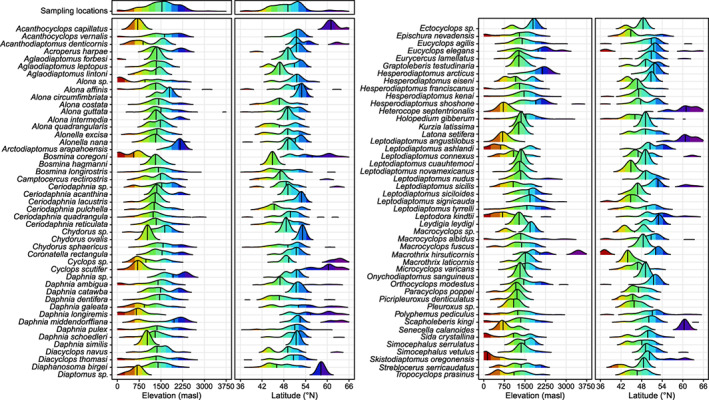
Ridgeline density plots for sampling locations and each zooplankton taxon (with at least three occurrences) showing distributions and median positions (vertical bars) across elevational and latitudinal gradients (colors correspond to zones defined in Figure 1).

**FIGURE 3:**
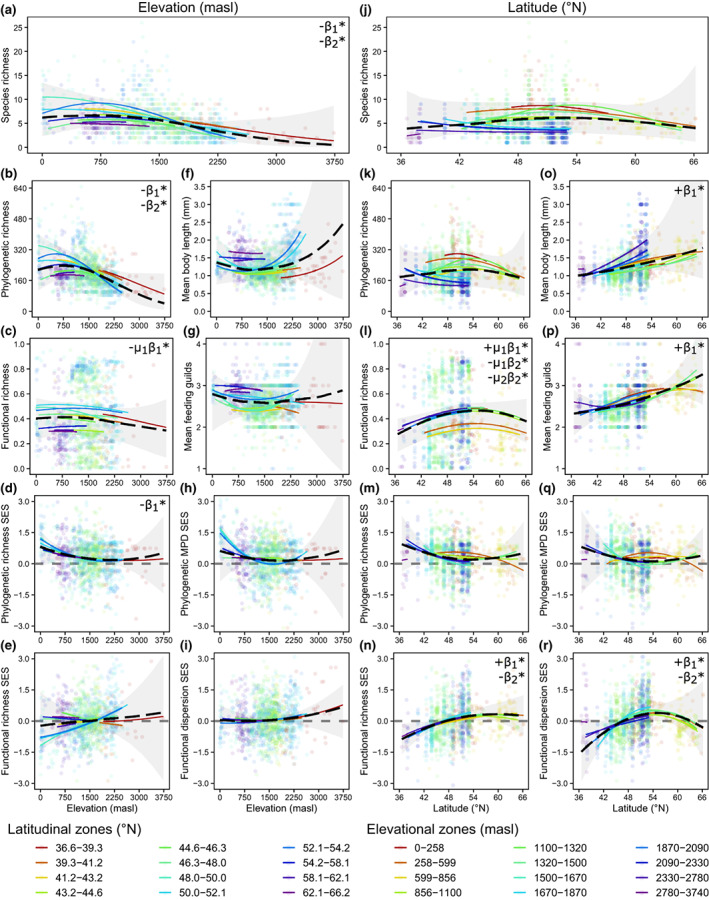
Population‐ and group‐level predictions (representing overall and zone‐specific trends presented as black dashed and colored lines, respectively) estimated from posterior draws of the linear predictor for generalized linear multilevel/mixed effects models of each biodiversity metric regressed on first‐ and second‐degree orthogonal polynomials of elevation (a–i) and latitude (j–r). Geographic predictors were centered and scaled (as *z*‐scores). Random effects were specified as latitudinal zones in elevation models and elevational zones in latitude models. Mean population‐level slope coefficients with 95% probability of being either positive or negative are noted in the top right corners of each plot for steepness (*β*_1_) and curvature (*β*_2_). Parameters for functional richness are from mixture models and assessed for each component separately (*μ*_1_ and *μ*_2_). Group‐level predictions are shown only for their distributional bounds. Shaded regions are population‐level 95% prediction intervals and points are individual sampling locations. Grey dashed horizontal lines on plots for standardized metrics indicate no difference from the null expectation. SES denotes standardized effect sizes and MPD denotes mean pairwise distance. See Table S5 for detailed results. Samples sizes are *n* = 1241 for species richness and community‐weighted means, *n* = 1157 for phylogenetic richness, phylogenetic richness SES, phylogenetic MPD SES, and functional dispersion SES, and *n* = 1024 for functional richness and functional richness SES.

**FIGURE 4:**
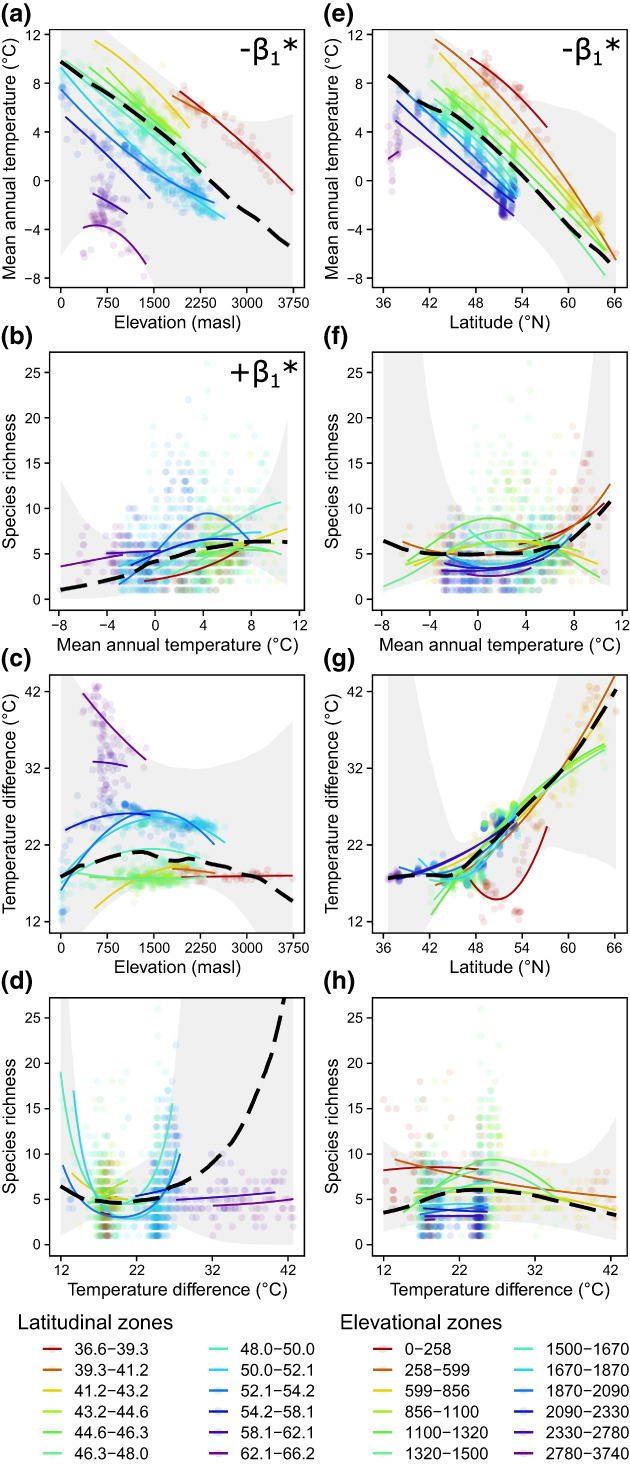
Population‐ and group‐level predictions (presented as black dashed and colored lines, respectively) estimated from posterior draws of the linear predictor for generalized linear multilevel/mixed effects models of species richness, elevation, and latitude regressed on first‐ and second‐degree orthogonal polynomials of mean annual temperature and temperature difference between the means of the warmest and coldest months. Both climate predictors and geographic variables were centered and scaled (as *z*‐scores). Random effects were specified as latitudinal zones (a–d) emphasizing elevation‐driven differences or elevational zones (e–h) emphasizing latitude‐driven differences. Mean population‐level slope coefficients with 95% probability of being either positive or negative are noted in the top right corners of each plot for steepness (*β*_1_) and curvature (*β*_2_). Group‐level predictions are shown only for their distributional bounds. Shaded regions are population‐level 95% prediction intervals and points are individual sampling locations. See Table S6 for detailed results and Figures S68 and S69 for relationships with other biodiversity metrics.

Zooplankton species ranged from widely dispersed (e.g. *Bosmina longirostris* and *Macrocyclops albidus*) to relatively localized across a small number of geographic zones (e.g. *Acanthocyclops capillatus* and *Chydorus ovalis*; Figure 2). Sampling locations were right skewed with respect to both latitude (median = 49.1°N) and elevation (median = 1538 m a.s.l.); however, the distributions of species varied. Overall, we saw that biodiversity patterns across elevations frequently differed from those across latitudes (population‐level effects shown as black dashed lines; Figure 3). Many of these variations resulted from interactions that emerged between latitude and elevation (group‐level effects shown as colored lines; Figure 3) linked to different underlying drivers (Figure 4). While our modelling approach precluded us from testing differences in elevational and latitudinal trends directly, we inferred differences based on qualitatively opposing trends across zones and interactions revealed through random effects. We explain these findings below and provide full details of model results in Tables S4–S6 and Figures S62–S69.

### 3.1 Trends across elevational and latitudinal gradients

Elevational declines in richness were similar across different dimensions of biodiversity (Figure 3a–c); however, population‐level slopes were steeper for species (*β*_1_ = −9.10) and phylogenetic (*β*_1_ = −5.93) than functional richness (*β*_1_ = −3.84 for *μ*_1_ and 1.72 for *μ*_2_; where *μ*_1_ and *μ*_2_ correspond to parameter estimates for each of two components of a mixture model; Table S5). Both the steepness (*β*_1_ coefficients for first‐degree orthogonal polynomial) and curvature (*β*_2_ coefficients for second‐degree orthogonal polynomials) of elevational effects were negative (CI <0) for species and phylogenetic richness, generating a low plateau relationship that peaked around 750 m a.s.l. Effects of latitude were similarly congruent across richness metrics but generated a mid‐peak pattern with maximum diversity at around 50°N (Figure 3j–l). Here, increases with latitude were greater for functional (*β*_1_ = 4.38 for *μ*_1_ and 2.27 for *μ*_2_) than species (*β*_1_ = 0.68) or phylogenetic richness (*β*_1_ = 0.15). Despite the hump‐shaped appearance of population‐level latitudinal richness gradients, the 95% credible intervals for the curvature of phylogenetic and species richness trends included zero, whereas the relationship was strongly negative (CI <0) for functional richness (*β*_2_ = −3.94 for *μ*_1_ and − 5.82 for *μ*_2_). Notably, the curvature of the species richness relationship was negative (CI <0) when modelled assuming naïveté of the different trends among elevational zones (i.e. group‐level effects; Figure S61).

Elevation gradients were comparatively flat and straight for functional dispersion SES (*β*_1_ = 1.78, *β*_2_ = 1.48; Figure 3i), and the same was true for functional richness SES at the population‐level despite steeper slopes in some mid‐latitude zones (*β*_1_ = 2.41, *β*_2_ = 0.08; Figure 3e). The slopes of phylogenetic richness SES and mean pairwise distance SES were both negative (*β*_1_ = −3.64 and − 2.05, respectively) and higher at low elevations, though only the former was consistently negative (CI <0; Figure 3d,h). Together, these results provide partial support for communities being overdispersed at low elevations, showing excess phylogenetic diversity in terminal branches (where more related species are less likely to co‐occur in montane lakes) but no consistent difference in the clustering of measured traits.

In contrast to negligible elevational effects, functional dispersion SES (*β*_1_ = 8.38, CI >0) and functional richness SES (*β*_1_ = 7.19, CI >0) responded positively with latitude up to around 50° N, revealing clustering (more similar body sizes and/or feeding strategies) at southern sites (Figure 3n,r). While population‐level latitudinal trends in phylogenetic richness SES (*β*_1_ = −2.65) and mean pairwise distance SES (*β*_1_ = −2.06) were qualitatively similar to those along elevational gradients suggesting possible overdispersion of relatedness at low latitudes, relationships were inverted in certain lower elevation zones and both credible intervals overlapped zero (Figure 3m,q and Figures S63 and S64). Body sizes increased (and became more variable) with elevation (*β*_1_ = 1.81) and latitude (*β*_1_ = 4.20), but only the latitudinal gradient was uniformly positive (CI >0; Figure 3f,o). Latitudinal relationships in mean feeding guilds were also positive (*β*_1_ = 2.20, CI >0) while elevational relationships were comparatively flat (*β*_1_ = 0.10; Figure 3g,p).

Species richness increased with habitat size, but despite the largest lakes occurring at lower elevations (with some bias towards greater depths at higher latitudes), interpretations of elevational and latitudinal gradients were unaffected (Table S7; Figure S70). We also discovered that geographic trends were robust to variations in sampling effort, as even though richness was generally higher at locations where more samples were collected, most sites (*n* = 813) were sampled only once and including sampling effort (either number of sampling events or years sampled) as a model covariate did not alter geographic predictions (Table S8; Figure S71).

### 3.2 Interactions between elevational and latitudinal gradients

Significant differences among geographic zones indicate differences in how latitudinal biodiversity gradients respond to differences in elevation, and that elevational patterns are moderated by latitude (Figure 3; Tables S4 and S5). The standard deviations of group‐level slope coefficients (and many intercepts) for biodiversity metrics included only positive values (CI > 0; Table S5), and information criteria showed poorer fits for models lacking random effects (generally increasing by at least 5 and in some cases more than 100; Table S4). For instance, intercepts for elevational richness gradients decreased at higher latitudes while intercepts for latitudinal gradients were generally lower at high elevations (Figure 3 and Figure S62). The effects of elevation on species richness were consistently negative but varied among groups and were steepest at mid‐latitudes (Figure 3a and Figure S63), possibly because the higher latitudinal zones had relatively short gradient lengths.

While biodiversity patterns generally varied among zones, the slopes and curvatures of richness relationships were more consistent across elevational than latitudinal gradients (Figure 3a–c vs 3j–l). Notably, population‐level estimates of latitudinal differences in species and phylogenetic richness overlapped zero (indicating no change). These trends arose because of contrasting relationships among elevational zones (a cross‐level interaction), producing a flat and weakly hump‐shaped overall effect (Figure 3j,k and Figures S63, S64). A positive slope (*μ*_1_ CI > 0) with greater curvature (*μ*_1_ and *μ*_2_ CI < 0) was observed for functional richness as a result of relatively more parallel shifts in function with increasing latitude (Figure 3l).

### 3.3 Climatic drivers of geographic diversity gradients

Differences between latitudinal and elevational diversity gradients were further illustrated by their associations to climate variables (Table S6; Figure 4 and Figures S65–S67), presenting temperature relationships to diversity when climate differences are either elevation‐driven (latitudinal zone random effect; Figure 4b,d) or along latitudinal gradients at different altitudes (elevational zone random effect; Figure 4f,h). Species richness increased with mean annual temperature when differences were driven by elevation (*β*_1_ = 9.20, CI > 0; Figure 4a,b). However, when mean temperatures corresponded to differences in latitude (Figure 4e,f), we found no clear population‐level trend because richness patterns depended on the elevations of the lakes (*β*_1_ = 1.69). This key distinction emerged despite strikingly similar elevational and latitudinal correlations to mean temperatures (*β*_1_ = −19.90 and − 17.41, respectively; CI < 0). Given these correlations, relationships to mean annual temperatures across other diversity metrics largely matched those with elevation and latitude (Figure S68). Some differences included functional richness and phylogenetic richness SES not responding as clearly to elevation‐driven temperature differences as the purely spatial elevation gradient (Figure 3c,d and Figure S68c,d), while body lengths decreased more consistently at warmer sites (Figure 3f and Figure S68f). Conversely, phylogenetic dispersion metrics increased more consistently with latitude‐driven mean temperature differences than simple latitude (Figure 3m,q and Figure S68m,q). Geographic gradients in seasonal temperature difference were weaker, showing variable associations to elevation (Figure 4c) while latitudinal correlations were generally positive (*β*_1_ = 10.73) but with credible intervals still crossing zero (Figure 4g). The effects of seasonality on species richness were inconsistent with both elevation and latitude (Figure 4d,h), though latitude‐driven differences were linked to body size, feeding guilds, and dispersion metrics (Figure S69).

## 4 DISCUSSION

We synthesized zooplankton community composition in over 1200 lakes in western North America to evaluate variation both within, and between, elevational and latitudinal gradients of freshwater biodiversity. We discovered that taxonomic, functional, and phylogenetic richness relationships were largely congruent within geographic gradients, while richness‐controlled metrics provided strong evidence of functional underdispersion (body size and trophic structure) at lower latitudes and phylogenetic overdispersion (across multiple niche dimensions) at lower elevations (Figure 3). However, richness consistently decreased at mid and high elevations, forming low plateaus, whereas it showed weakly positive and nonmonotonic trends that formed mid‐gradient humps with latitude (partially supporting H1a). In addition to illustrating how the effects of latitude depend on elevation, these findings suggest that commonly observed hump‐shaped diversity gradients can emerge from aggregating disparate responses across elevational zones (i.e. geographic interactions), rather than any strictly latitudinal phenomena. The lack of a consistent latitudinal diversity gradient was reflected in the lack of consistent associations between species richness and either mean temperature or seasonality linked to differences in latitude (Figure 4). In contrast, richness did increase when differences in mean temperature were driven by elevation. Our findings support a growing body of evidence showing how temperature limits the diversity of freshwater ectotherms along elevational gradients (e.g. Brucet et al., 2013; Lyons & Vinebrooke, 2016; Pinel‐Alloul et al., 2013; Stomp et al., 2011) but suggest different processes are at play with latitude, indicating limited equivalence in their capacity to provide climate‐change refuge to migrating species.

### 4.1 Trends across elevational and latitudinal gradients

Geographic patterns in biodiversity remain contentious despite their rich history of study (Brown, 2014; Kinlock et al., 2018; Rahbek et al., 2019). Lakes and ponds are useful model systems as they provide discrete sampling locations that integrate biotic interactions, dispersal limitation, and other important processes enabling local coexistence. Lake size is also relatively independent of geographic gradients (though the largest lakes occur at lower elevation; Hessen et al., 2007), meaning that lacustrine ecosystems avoid the common conflation of elevation and area along terrestrial gradients (Quintero & Jetz, 2018; Rahbek, 1995). Yet, despite increasing synthesis of broadscale data and the importance of freshwater ecosystems to global biodiversity and human well‐being, our understanding of macroecological patterns and processes in freshwaters lags that of other realms (Heino, 2011; Kinlock et al., 2018). Past studies have shown declining phytoplankton richness with increasing elevation linked to local productivity of lakes across the continental USA (Stomp et al., 2011), while stream diatoms either increased, decreased, or showed no significant relationship to elevation across different regions in Europe and China (Wang et al., 2017). Declining zooplankton richness with increasing elevation has also been observed in multiple regions (e.g. Hessen et al., 2006; Lyons & Vinebrooke, 2016; Shurin et al., 2007), but as with other taxa, interactions between gradients and across multiple dimensions of biodiversity are largely unexplored.

Comparative studies across multiple dimensions of biodiversity offer insights into community assembly (e.g. Cai et al., 2018; Jarzyna et al., 2021; Montaño‐Centellas et al., 2020; Qian et al., 2020). We found that richness metrics were generally congruent within gradients, but consistent with hypothesis H1b, declines in taxonomic richness with altitude were steeper than those for function or phylogeny (Figure 3a–c). These results indicate some potential for communities to maintain trait and phylogenetic diversity even as species are lost. Zooplankton communities also displayed increasing functional richness with latitude but no clear difference in species or phylogenetic richness (Figure 3j–l), indicative of past meta‐analyses that found weaker, nonsignificant, latitudinal diversity gradients in freshwaters than terrestrial or marine habitats (among a relative paucity of freshwater studies; Hillebrand, 2004; Kinlock et al., 2018). Here, functional differences likely reflect greater and more variable body lengths and top‐heavy food chains at higher latitudes (whereas elevational patterns were flatter and less consistent; Figure 3f,g,o,p). These findings provided partial support for hypothesis H1c (larger species in northern lakes) but opposed hypothesis H1d. Here, greater average feeding guild positions at higher latitudes indicate that cooler temperatures limit diversity more at lower trophic levels. For example, smaller, specialized grazers might be replaced by larger, generalist omnivores in unproductive, northern lakes, possibly due to stronger thermal constraints on feeding rates or digestion of lower quality herbivorous diets (Behrens & Lafferty, 2007). Alternatively, northern lakes may have more specialized zooplankton predators. Body sizes are also impacted by fish predation (Loewen et al., 2020); however, the propensity for fish absence in alpine lakes would be expected to strengthen, not weaken, elevational relationships relative to those with latitude.

Standardized metrics showed phylogenetic overdispersion of terminal branches at lower elevations, providing partial support for hypothesis H1e (Figure 3d). Treating phylogeny as a proxy for multiple conserved and unmeasured traits (e.g. stoichiometry, motility, and pigmentation), these findings suggest that richer communities in more productive montane lakes are comparatively more structured by competitive exclusion of closely related species (e.g. Bryant et al., 2008; Graham et al., 2009; Lesser et al., 2020; Violle et al., 2011). Similar trends of phylogenetically inferred reductions in niche size and overlap were found with increasing latitude, though these relationships were weaker (credible intervals included zero; Figure 3m). Despite these results, relationships between phylogenetic dispersion and elevation‐driven temperature gradients were less consistent (CI overlapped zero; Figures S68d,h and S69d,h), indicating potential roles for other factors mediating niche differentiation of co‐occurring species (e.g. nutrient and ion chemistry). In contrast, phylogenetic dispersion increased with warming and reduced seasonality when climatic differences were driven by latitude (Figures S68m,q and S69m,q), even though purely spatial trends with latitude were not consistently negative across elevational zones. These findings highlight the relatively greater role of temperature differences along latitudinal than elevational gradients.

Function showed the opposite trend with latitude, where communities were underdispersed at more southern sites (Figure 3n,r), and no trend with elevation across latitudinal zones (Figure 3e,i). Although counter to our expectation of environmental filters driving greater trait clustering (selecting for tolerant species) in the north, our measure of functional diversity was based on only two traits (body size and feeding guild), both of which could be expected to influence fitness differences among species. The relative narrowing of these key functional traits in warmer and less seasonal lakes at lower latitudes may thus reflect differences in competitive dominance over limiting resources (Mayfield & Levine, 2010), enhanced, size‐selective predation evasion (Loewen et al., 2020), or differences in evolutionary history (Qian et al., 2020). Alternatively, higher temperatures in the south might filter out larger species more so than harsh climates exclude small species in northern regions. While our observational approach cannot separate these competing processes, we found that phylogenetic measures provided a valuable complement to functional metrics constructed from low‐dimensional trait information. Whatever the cause of functional and phylogenetic dispersion, different elevational and latitudinal patterns point to differences in how communities are assembled along these gradients and their sensitivities to climate.

### 4.2 Interactions between elevational and latitudinal gradients

Integrating data from several adjacent transects, we found greater difference in communities along elevational than latitudinal gradients. This was expected as climate factors drive greater vertical than horizontal heterogeneity in mountain regions (Rahbek et al., 2019) and environmental differences are further mediated by the downward flow of water (Kratz et al., 1997). However, we also found considerable variation, or nonstationarity, across zones. For instance, latitudinal diversity gradients differed between lakes at lower and higher elevations (H2). We found similar differences among elevation gradients with greater rates of change at mid‐latitudes, though gradient lengths differ, and scale effects may bias group‐level estimates for incomplete gradients (i.e. producing flatter slopes for lakes at higher latitudes with limited elevational distributions; Nogués‐Bravo et al., 2008). Contrasting positive and negative relationships across elevational zones led to an overall hump‐shaped latitudinal richness gradient. While often observed at broader spatial scales, we are not aware of any prior studies that have shown how mid‐gradient diversity peaks can emerge from aggregating transects with opposing trends (i.e. a geographic interaction).

### 4.3 Climatic drivers of geographic diversity gradients

Over 30 hypotheses have been proposed to explain latitudinal diversity gradients (Brown, 2014), many of which have been also applied to elevation, and freshwater biodiversity trends are often linked to climate (Dodds et al., 2019). For example, the richness‐energy hypothesis has previously been invoked to explain zooplankton diversity in relation to patterns of solar radiation across Canada (Pinel‐Alloul et al., 2013). However, rigorous tests comparing mean temperature and seasonality constraints on biodiversity are limited. Counter to our predictions (H3), we found greater support for the energy‐richness hypothesis (inferred from response to mean air temperatures) than that of physiological tolerance to the breadth of temperature conditions experienced, suggesting that reduced productivity (from environmental harshness) limits zooplankton diversity along elevational gradients. Given the generally narrow thermal‐safety margins of ectotherms (Sunday et al., 2014), our findings point to behavioral plasticity limiting exposure to harmful temperatures. For example, species may use different habitats within a lake or produce resting eggs to diapause during unfavorable conditions (Holm et al., 2018).

Although air and water temperatures are closely related, aquatic communities are also physically buffered from climate variations. For instance, deeper lakes may thermally stratify during the summer, offering refuge from warming to mobile species at greater depths, and communities under ice cover are protected from below‐freezing temperatures above (Dodds et al., 2019). Temperature fluctuations are further limited by glacial inputs in many mountain lakes, though annual ice is receding rapidly (Clarke et al., 2015). Greater refuge from climatic variability and extreme events may have contributed to tighter thermal‐safety margins for aquatic ectotherms than their terrestrial counterparts, paradoxically increasing their vulnerability to future climate changes (Pinsky et al., 2019). While communities facing greater seasonality at higher latitudes might be expected to possess greater thermal tolerance than those restricted to a narrower range of conditions (Janzen, 1967; Pinsky et al., 2019; Rahbek et al., 2019), cooler environments also decrease developmental rates and select for species with greater size at maturity (Atkinson, 1994; Walters & Hassall, 2006). Here, the larger average sizes of zooplankton in colder environments indicate heightened, yet potentially differing sensitivities of northern and alpine communities to future warming (Daufresne et al., 2009).

## 5 CONCLUSIONS

The different gradients revealed by our analyses have implications for substituting latitude for elevation (and vice versa) in modelling climate change adaptation. Our findings support past observations of increasing seasonality with latitude but not elevation (Rahbek, 1995), while mean annual temperatures were closely related to both. However, species richness was only linked to mean temperature, and only when mean temperature was driven by a difference in elevation. Thus, while some species may adapt to climate change by tracking shifting conditions with elevation, differences with latitude may be less consistent, complicating ecological forecasts. Different underlying processes confound latitudinal patterns and their capacity to offer climate refuge. For instance, latitudinal gradients may be linked to geological setting or differences in land use, though freshwater diversity is usually linked to natural factors at broader, continental scales (e.g. Brucet et al., 2013; Loewen et al., 2019; Stomp et al., 2011). Latitudinal gradients are also influenced by historical glaciation (Henriques‐Silva et al., 2016), where some communities may have escaped ice coverage while others experienced varying degrees of post‐glacial isolation (e.g. Millette et al., 2011). Differences in upward and poleward connectivity may constrain recolonization patterns, as zooplankton are readily dispersed by hydrological connections as well as wind and animal vectors (e.g. Loewen et al., 2019; Vanschoenwinkel et al., 2008) but communities may experience a lag while organisms track changing conditions (Alexander et al., 2018). Whatever additional factors are driving latitudinal patterns, we discovered that communities tracked climate differences with elevation more consistently than latitude, indicating potentially greater barriers to poleward migration for organisms under increasing warming. However, despite their closer associations to climate, elevational gradients can only offer so much relief as mountains are only so tall, and communities in alpine regions may struggle to adapt.

The types, sizes, and functional attributes of zooplankton have broad consequences for lakes and other aquatic ecosystems; however, our inferences have several limitations. In addition to issues of varying habitat size and sampling effort (see Appendix S3), our study relies on surrogate phylogenies, uses limited trait information, and does not account for temporal change in zooplankton communities or climate. Missing branch lengths represent a loss of information about phylogenetic structure; however, the strong morphological basis for zooplankton taxonomy supports our use of taxonomic surrogates, which have been successfully applied in similar contexts (e.g. Cai et al., 2018; Crozier et al., 2005) and shown to correlate strongly with complete, time‐calibrated phylogenies (Ricotta et al., 2012). Still, future studies will benefit from more detailed molecular information to construct evolutionary histories, as well as measurement of additional traits across a broad range of taxa. Where available, integration of time‐series data would help to understand turnover along elevational and latitudinal gradients (including changes driven by climate). For instance, seasonal sampling may capture additional species with different thermal optima, and interannual sampling may capture biological responses to warming trends. Paleolimnological records offer an alternative historical perspective, especially where long‐term monitoring is unavailable (Smol et al., 1991).

While ecogeographic rules do not necessarily apply across taxa (Hof et al., 2008) or continents (Alahuhta et al., 2020) as groups may have distinct evolutionary histories and underlying spatial or environmental drivers (Qian et al., 2020; Wang et al., 2017), our model system shows how biodiversity may be differentially structured along geographic temperature gradients. Important questions remain about which spatial or environmental factors are driving differences among observed gradients and how individual species are most likely to respond to changing climate given their varying traits for dispersal, competition, and thermal niche. For example, zooplankton may vary in their ability to overcome physical or climatic dispersal barriers and track shifting conditions (e.g. Loewen et al., 2019). Our findings have significant consequence for climate change adaptation and should be considered for other temperature‐sensitive taxa and regions.

## AUTHOR CONTRIBUTIONS

All authors contributed to study design and manuscript revision. Charlie J. G. Loewen conceived of the study, conducted the analyses, and composed the initial draft manuscript.

## CONFLICT OF INTEREST

The authors declare that they have no competing interests.

## Supporting information


Appendix S1.
Appendix S2.
Appendix S3.
Table S1.
Table S2.
Table S3.
Table S4.
Table S5.
Table S6.
Table S7.
Table S8.
Figure S1.
Figure S2.
Figures S3–S60.
Figure S61.
Figure S62.
Figure S63.
Figure S64.
Figure S65.
Figure S66.
Figure S67.
Figure S68.
Figure S69.
Figure S70.
Figure S71.

## Data Availability

The data and code that support the findings of this study are openly available in Dryad at https://doi.org/10.5061/dryad.905qfttpb and GitHub at https://github.com/loewenecology/Geographic‐gradients‐of‐zooplankton‐diversity.
