# Supplementary material for: Biodiversity patterns diverge along geographic temperature gradients
Source: Glob Chang Biol. 2022 Nov 16;29(3):603–17. doi: 10.1111/gcb.16457 (PMC10100522; doi:10.1111/gcb.16457)
Supplement: Supplementary file 1 — Appendix S1. Appendix S2. Appendix S3. Table S1. Table S2. Table S3. Table S4. Table S5. Table S6. Table S7. Table S8. Figure S1. Figure S2. Figures S3–S60. Figure S61. Figure S62. Figure S63. Figure S64. Figure S65. Figure S66. Figure S67. Figure S68. Figure S69. Figure S70. Figure S71. [file GCB-29-603-s001.docx]

**Supporting Information.** Charlie J.G. Loewen et al., Biodiversity patterns diverge along geographic temperature gradients

**Supporting Appendices**

**Appendix S1. Details for calculating biodiversity metrics**

**Appendix S2. Details for model comparisons and checks**

**Appendix S3. Habitat size and sampling effort sensitivity analyses**

**Supporting Tables**

**Table S1.** Species counts and trait assignments

**Table S2.** Generalized linear multilevel/mixed effect model specifications

**Table S3.** Results for traditional interaction models of biodiversity metrics regressed on elevation, latitude, and their multiplicative interaction

**Table S4.** Summary of information criteria comparing models with and without random effects for simple linear and second-degree geographic orthogonal polynomial regression models

**Table S5.** Detailed results for generalized linear multilevel/mixed effect models of each biodiversity metric regressed on first- and second-degree orthogonal polynomials of elevation (a) and latitude (b)

**Table S6.** Detailed results for generalized linear multilevel/mixed effect models of biodiversity, elevation, and latitude regressed on first- and second-degree orthogonal polynomials of mean annual temperature (a) and temperature difference between the means of the warmest and coldest months (b)

**Table S7.** Results for generalized linear multilevel/mixed effect models evaluating relationships between waterbody area, depth, elevation, latitude, and species richness

**Table S8.** Results for generalized linear multilevel/mixed effect models evaluating relationships between sampling effort, sampling year, elevation, latitude, and species richness

**Supporting Figures**

**Figure S1.** Fan plot of taxonomic rankings and feeding traits (substrate-grazing taxa = green; seston-filtering taxa = blue; stationary suspension-feeding taxa = purple; and raptorial-feeding taxa = red).

**Figure S2.** Predictions estimated from posterior draws of the linear predictor for generalized linear interaction models of each biodiversity metric regressed on latitude, elevation, and their multiplicative interaction.

**Figure S3–S60.** Posterior predictive checks for generalized linear multilevel/mixed effect models showing kernel density estimates for each of 100 draws from the posterior distribution (*y*_rep_) overlain with that of the response variable (*y*).

**Figure S61.**  Predictions estimated from posterior draws of the linear predictor for generalized linear (negative binomial) fixed effect models of species richness regressed on first- and second-degree orthogonal polynomials of elevation (a), latitude (b), mean annual temperature (c), and temperature difference between the means of the warmest and coldest months (d).

**Figure S62.** Mean group-level intercepts for generalized linear multilevel/mixed effect models of each biodiversity metric regressed on first- and second-degree orthogonal polynomials of elevation and latitude.

**Figure S63.** Mean group-level β_1_ coefficients for generalized linear multilevel/mixed effect models of each biodiversity metric regressed on first- and second-degree orthogonal polynomials of elevation and latitude.

**Figure S64.** Mean group-level β_2_ coefficients for generalized linear multilevel/mixed effect models of each biodiversity metric regressed on first- and second-degree orthogonal polynomials of elevation and latitude.

**Figure S65.** Mean group-level intercepts for generalized linear multilevel/mixed effect models of species richness, elevation, and latitude regressed on first- and second-degree orthogonal polynomials of (a) mean annual temperature and (b) temperature difference between the means of the warmest and coldest months.

**Figure S66.** Mean group-level β_1_ coefficients for generalized linear multilevel/mixed effect models of species richness, elevation, and latitude regressed on first- and second-degree orthogonal polynomials of (a) mean annual temperature and (b) temperature difference between the means of the warmest and coldest months.

**Figure S67.** Mean group-level β_2_ coefficients for generalized linear multilevel/mixed effect models of species richness, elevation, and latitude regressed on first- and second-degree orthogonal polynomials of (a) mean annual temperature and (b) temperature difference between the means of the warmest and coldest months.

**Figure S68.** Population- and group-level predictions (representing overall and zone-specific trends presented as black dashed and coloured lines, respectively) estimated from posterior draws of the linear predictor for generalized linear multilevel/mixed effects models of each biodiversity metric regressed on first- and second-degree orthogonal polynomials of mean annual temperature.

**Figure S69.** Population- and group-level predictions (representing overall and zone-specific trends presented as black dashed and coloured lines, respectively) estimated from posterior draws of the linear predictor for generalized linear multilevel/mixed effects models of each biodiversity metric regressed on first- and second-degree orthogonal polynomials of mean temperature difference.

**Figure S70.** Population-level predictions (overall trends presented as black dashed lines) estimated from posterior draws of the linear predictor for generalized linear multilevel/mixed effects models evaluating relationships with waterbody area (a–e) and depth (f–j).

**Figure S71.** Histograms presenting number of sampling events (a) and years sampled (e) at each site and population-level predictions (overall trends presented as black dashed lines) estimated from posterior draws of the linear predictor for generalized linear multilevel/mixed effects models (negative binomial regression) evaluating of species richness regressed on elevation, latitude, number of sampling events (b–d) and years sampled (f–h).

**Appendix S1. Details for calculating biodiversity metrics**

For functional richness, a reduced trait space was obtained from principal coordinates analysis (PCoA) of square root-transformed dissimilarities (to avoid negative eigenvalues) and used to calculate the convex hulls of local communities (values standardized between zero and one). Due to requirements for the number of species to exceed the number of traits, convex hulls were based on two PCoA axes (quality of representation = 0.731) and defined for communities with at least three species. For our incidence-based measure of functional dispersion, a full trait space was estimated from PCoA applied to raw Gower’s dissimilarities and used to calculate the mean distances of species to community centroids (a minimum of two species were required).

Standardized effect sizes were calculated as the probit-transformation of quantile p-values comparing the diversity of a given community to that estimated for a series of random communities (rank of observed value / total number of random communities + 1), where random communities were generated using an independent swap algorithm maintaining species occurrence frequency (column sums) and sample richness (row sums; Gotelli 2000). Null communities were estimated separately for each elevational and latitudinal zone (separate site and species pools), using the *randomizeMatrix* function from the ‘picante’ package (Kembel et al., 2010) to obtain 1,000 replicates (each generated by 10,000 random swaps). Effect size estimates were highly correlated with the standardized differences between observed values and the mean of the random communities but avoid the implicit assumption that the distribution of null values is symmetric around the mean (Lhotsky et al., 2016). While the use of literature measurements prevented us from capturing intraspecific variations in size, species body lengths ranged from 0.22–8.00 mm.

REFERENCES

Gotelli, N.J. (2000). Null model analysis of species co-occurrence patterns. *Ecology*, 81, 2606–2621.

Kembel, S.W., Cowan, P.D., Helmus, M.R., Cornwell, W.K., Morlon, H., Ackerly, D.D., Blomberg, S.P., & Webb, C.O. (2010). Picante: R tools for integrating phylogenies and ecology. *Bioinformatics*, 26, 1463–1464.

Lhotsky, B., Kovács, B., Ónodi, G., Csecserits, A., Rédei, T., Lengyel, A., Kertész, M., & Botta-Dukát, Z. (2016). Changes in assembly rules along a stress gradient from open dry grasslands to wetlands. *Journal of Ecology*, 104, 507–517.

**Appendix S2. Details for model comparisons and checks**

Both the widely applicable (WAIC) and leave-one-out cross-validation information criteria (LOOIC) showed equal or better performance for geographic biodiversity models fitted with elevational or latitudinal random effects than fixed effects alone (Table S3). Similarly, information criteria indicated better fit for polynomial than simple linear models for each response except functional richness (across latitudes), functional richness SES (across latitudes), and functional dispersion SES (across elevations), indicating that linear predictors would have sufficed for these models. Markov chains showed strong convergence for all models except functional richness (assessed by potential scale reduction factors, $\hat{R}$, being less than or equal to 1.01). Functional richness was bimodally distributed between zero and one and fit by a (beta, beta) mixture model, with priors for centered population-level intercepts set to follow a student’s t-distribution with different locations (μ_1_ = -0.5 and μ_2_ = 0.5) and reduced scale (σ = 0.2) to improve convergence (and avoid samplers jumping between modes). Models for functional richness were also run for an extended number of iterations (9,000) but returned elevated $\hat{R}$ values for several parameters (Tables S5 and S6), indicating potentially unreliable estimates. Posterior predictive checks, including density overlay plots, showed generally good fit to observed data (Figures S3–S60).

**Appendix S3. Habitat size and sampling effort sensitivity analyses**

Despite there being a much weaker link between habitat size and elevation in lakes than terrestrial systems, we found that the largest and deepest sampling locations were at lower elevations, with a positive association between depth and latitude (Table S7, Fig S70). In addition to potentially offering more room and diversity of habitats to more species (species-area relationship; Preston 1962), the limnetic communities of larger lakes may differ from smaller ponds in their connection to the surrounding benthic, littoral, and terrestrial environments. For instance, smaller waterbodies lacking fish often experience greater invertebrate predation and may be more isolated on the landscape (Søndergaard et al., 2005). While species richness generally increased with both area and depth, our sensitivity analyses showed that elevational and latitudinal diversity gradients were unaffected by variation in habitat size.

Another limitation of our study was that sampling effort varied among sites. While providing better estimates of ‘true’ species diversity than the common approach of compiling communities from overlapping species’ range maps without any direct evidence of co-occurrence, our data synthesis presents a trade-off between generality and specificity. Here, we accepted variation in sampling effort (number of individuals counted, samples collected, and years sampled) as a source of heterogeneity in order to capture trends across a larger number of communities spanning broad geographic gradients. Rarefaction is often used to obtain estimates of species density accounting for biases in the detection of rare species at sites with greater sampling, but this approach introduces a different source of error by throwing out information about species known to occur in more heavily sampled locations. Another issue is that sampling across multiple years may capture turnover (i.e. species arriving after the initial sampling event). While species richness generally increased with greater sampling effort (both number of events and number of years; Table S8, Fig S71), we were unable to rarify our data because the number of individuals counted was rarely reported and results from multiple sampling events/years were often presented in aggregate (i.e. without specifying which species were found in which samples). However, despite concerns about undersampling possibly biasing certain species richness estimates, any errors should be abated by our large sample size, use of occurrence (rather than abundance) data analyzed with zonal random effects (capturing minor disparities in regional sampling protocols with unique slope and intercept terms), and the relatively simple communities with limited seasonal variation in isolated mountain lakes (mean richness = 5.3). For example, Shurin et al., (2007) found generally low rates of turnover in mountain zooplankton communities and strong correlations between richness estimates from different time scales (i.e. single or multiple samples). Similarly, Loewen et al., (2019) found that zooplankton composition patterns were largely robust to differences in sampling effort and year. Our sensitivity analyses corroborate these findings, as our interpretations of elevational and latitudinal diversity gradients were robust to variation in sampling effort.

REFERENCES

Loewen, C.J.G., Strecker, A.L., Larson, G.L., Vogel, A., Fischer, J.M., & Vinebrooke, R.D. (2019). Macroecological drivers of zooplankton communities across the mountains of western North America. *Ecography*, 42, 791–803.

Preston, F.W. (1962). The canonical distribution of commonness and rarity: part 1. *Ecology*, 43, 185–215.

Shurin, J.B., Arnott, S.E., Hillebrand, H., Longmuir, A., Pinel-Alloul, B., Winder, M., & Yan, N.D. (2007). Diversity-stability relationship varies with latitude in zooplankton. *Ecology Letters*, 10, 127–134.

Søndergaard, M., Jeppesen, E., & Jensen, J.P. (2005). Pond or lake: does it make any difference? *Archiv für Hydrobiologie*, 162, 143–165.

**Table S1.** Species counts and trait assignments

| **Study taxa** | **Site count** | **Length (mm)** | **Length**  **reference** | **Reference taxa** | **Feeding**  **guild** |
| --- | --- | --- | --- | --- | --- |
| Acanthocyclops capillatus | 8 | 2.00 | Edmondson.1959 | Cyclops capillatus | 4 |
| Acanthocyclops vernalis | 308 | 0.82 | Loewen.2016 | Acanthocyclops vernalis | 4 |
| Acanthodiaptomus denticornis | 23 | 1.65 | MacLennan.2015 | Acanthodiaptomus denticornis | 3 |
| Acroperus harpae | 17 | 0.95 | Hébert.2016; CIEE.2012 | Acroperus harpae | 1 |
| Aglaodiaptomus forbesi | 14 | 1.50 | Edmondson.1959 | Pseudodiaptomus forbesi | 3 |
| Aglaodiaptomus leptopus | 123 | 1.81 | Loewen.2016 | Aglaodiaptomus leptopus | 3 |
| Aglaodiaptomus lintoni | 10 | 2.00 | Edmondson.1959 | Diaptomus lintoni | 3 |
| Alona | 17 | 0.43 | Genus mean | Genus mean | 1 |
| Alona affinis | 48 | 0.60 | Hébert.2016; CIEE.2012 | Alona affinis | 1 |
| Alona circumfimbriata | 3 | 0.40 | Balcer.1984 | Alona circumfimbriata | 1 |
| Alona costata | 44 | 0.45 | MacLennan.2015 | Alona costata | 1 |
| Alona guttata | 73 | 0.33 | MacLennan.2015 | Alona guttata | 1 |
| Alona intermedia | 5 | 0.36 | MacLennan.2015 | Alona intermedia | 1 |
| Alona quadrangularis | 11 | 0.43 | Hébert.2016; CIEE.2012 | Alona quadrangularis | 1 |
| Alonella excisa | 15 | 0.30 | MacLennan.2015 | Alonella excisa | 1 |
| Alonella exigua | 1 | 0.35 | Edmondson.1959 | Alonella exigua | 1 |
| Alonella nana | 38 | 0.28 | Edmondson.1959 | Anonella nana | 1 |
| Arctodiaptomus arapahoensis | 18 | 1.25 | Loewen.2016 | Arctodiaptomus arapahoensis | 3 |
| Bosmina coregoni | 88 | 0.44 | Hébert.2016; Barnett.2007 | Bosmina longispina | 2 |
| Bosmina hagmanni | 39 | 0.35 | Loewen.2016 | Bosmina longirostris | 2 |
| Bosmina longirostris | 374 | 0.35 | Loewen.2016 | Bosmina longirostris | 2 |
| Camptocercus rectirostris | 15 | 1.00 | Edmondson.1959 | Camptocercus rectirostris | 1 |
| Ceriodaphnia | 13 | 0.63 | Genus mean | Genus mean | 2 |
| Ceriodaphnia acanthina | 6 | 0.54 | MacLennan.2015 | Ceriodaphnia acanthina | 2 |
| Ceriodaphnia lacustris | 17 | 0.85 | Hébert.2016; Barnett.2007 | Ceriodaphnia lacustris | 2 |
| Ceriodaphnia pulchella | 31 | 0.55 | Hébert.2016; CIEE.2012 | Ceriodaphnia pulchella | 2 |
| Ceriodaphnia quadrangula | 64 | 0.55 | Loewen.2016 | Ceriodaphnia quadrangula | 2 |
| Ceriodaphnia reticulata | 106 | 0.68 | MacLennan.2016 | Ceriodaphnia reticulata | 2 |
| Chydorus | 24 | 0.44 | Genus mean | Genus mean | 1 |
| Chydorus ovalis | 3 | 0.50 | Edmondson.1959 | Chydorus ovalis | 1 |
| Chydorus sphaericus | 451 | 0.37 | MacLennen.2015 | Chydorus sphaericus | 1 |
| Coronatella rectangula | 27 | 0.22 | MacLennan.2015 | Alona rectangula | 1 |
| Cyclops | 29 | 1.59 | Genus mean | Genus mean | 4 |
| Cyclops scutifer | 114 | 1.50 | Hébert.2016; CIEE.2012 | Cyclops scutifer | 4 |
| Daphnia | 33 | 1.44 | Genus mean | Genus mean | 2 |
| Daphnia ambigua | 59 | 0.88 | Hébert.2016; CIEE.2012 | Daphnia ambigua | 2 |
| Daphnia catawba | 15 | 1.30 | MacLennen.2015 | Daphnia catawba | 2 |
| Daphnia dentifera | 458 | 1.17 | MacLennen.2015 | Daphnia dentifera | 2 |
| Daphnia galeata | 90 | 1.50 | Hébert.2016; CIEE.2012 | Daphnia galeata-mendotae | 2 |
| Daphnia laevis | 1 | 1.60 | Hébert.2016; CIEE.2012 | Daphnia laevis | 2 |
| Daphnia longiremis | 88 | 0.84 | Hébert.2016; CIEE.2012 | Daphnia longiremis | 2 |
| Daphnia middendorffiana | 126 | 2.45 | Hébert.2016; CIEE.2012 | Daphnia middendorffiana | 2 |
| Daphnia parvula | 2 | 1.10 | Hébert.2016; CIEE.2012 | Daphnia parvula | 2 |
| Daphnia pulex | 285 | 2.00 | MacLennen.2015 | Daphnia pulex | 2 |
| Daphnia retrocurva | 1 | 0.87 | Hébert.2016; CIEE.2012 | Daphnia retrocurva | 2 |
| Daphnia schoedleri | 75 | 1.93 | MacLennen.2015 | Daphnia schodleri | 2 |
| Daphnia similis | 5 | 1.60 | MacLennen.2016 | Daphnia similis | 2 |
| Diacyclops navus | 21 | 0.72 | Loewen.2016 | Diacyclops navus | 4 |
| Diacyclops thomasi | 224 | 0.92 | Hébert.2016; CIEE.2012 | Diacyclops bicuspidatus-thomasi | 4 |
| Diaphanosoma birgei | 193 | 0.97 | Loewen.2016 | Diaphanosoma birgei | 2 |
| Diaptomus | 6 | 0.60 | Hébert.2016; CIEE.2012 | Diaptomids (genus) | 3 |
| Disparalona hamata | 1 | 0.63 | Neretina.2018; Birge.1879 | Disparalona hamata | 1 |
| Disparalona leei | 2 | 0.46 | Neretina.2018; Shing-Ming.1970 | Disparalona leei | 1 |
| Disparalona rostrata | 2 | 0.52 | Neretina.2018; Koch.1841 | Disparalona rostrata | 1 |
| Drepanothrix dentata | 1 | 0.70 | Edmondson.1959 | Drepanothrix dentata | 1 |
| Ectocyclops | 4 | 1.08 | Edmondson.1959 | Ectocyclops phaleratus | 4 |
| Epischura nevadensis | 157 | 1.75 | Hébert.2016; CIEE.2012 | Epischura nevadensis | 4 |
| Eucyclops agilis | 126 | 0.86 | Loewen.2016 | Eucyclops agilis | 4 |
| Eucyclops elegans | 18 | 0.92 | Hébert.2016; CIEE.2012 | Eucyclops elegans | 4 |
| Eurycercus lamellatus | 24 | 2.70 | Hébert.2016; Lynch.1980 | Eurycercus lamellatus | 1 |
| Graptoleberis testudinaria | 7 | 0.60 | Edmondson.1959 | Graptoleberis testudinaria | 1 |
| Hesperodiaptomus arcticus | 201 | 3.00 | Edmondson.1959 | Diaptomus arcticus | 3 |
| Hesperodiaptomus caducus | 1 | 2.85 | Edmondson.1959 | Diaptomus caducus | 3 |
| Hesperodiaptomus eiseni | 3 | 3.30 | Edmondson.1959 | Diaptomus eiseni | 3 |
| Hesperodiaptomus franciscanus | 76 | 1.55 | Edmondson.1959 | Diaptomus franciscanus | 3 |
| Hesperodiaptomus hirsutus | 2 | 1.84 | Edmondson.1959 | Diaptomus hirsutus | 3 |
| Hesperodiaptomus kenai | 182 | 2.40 | Edmondson.1959 | Diaptomus kenai | 3 |
| Hesperodiaptomus nevadensis | 2 | 3.78 | Edmondson.1959 | Diaptomus nevadensis | 3 |
| Hesperodiaptomus novemdecimus | 2 | 3.78 | Edmondson.1959 | Diaptomus novemdecimus | 3 |
| Hesperodiaptomus shoshone | 64 | 3.30 | Edmondson.1959 | Diaptomus shoshone | 3 |
| Hesperodiaptomus victoriaensis | 1 | 2.70 | Edmondson.1959 | Diaptomus victoriaensis | 3 |
| Heterocope septentrionalis | 59 | 2.50 | Luecke & O’Brien.1981 | Heterocope septentrionalis | 3 |
| Holopedium gibberum | 375 | 1.04 | Hébert.2016; Barnett.2007 | Holopedium gibberum | 2 |
| Homocyclops ater | 2 | 2.90 | Marten & Reid.2007 | Homocyclops Ater | 4 |
| Ilyocryptus spinifer | 1 | 0.80 | Edmondson.1959 | Ilyocryptus spinifer | 1 |
| Kurzia latissima | 3 | 0.50 | Edmondson.1959 | Kurzia latissima | 1 |
| Latona setifera | 5 | 2.25 | Edmondson.1959 | Latona setifera | 2 |
| Leptodiaptomus | 1 | 1.21 | Genus mean | Genus mean | 3 |
| Leptodiaptomus angustilobus | 86 | 1.40 | Edmondson.1959 | Diaptomus pribilofensis | 3 |
| Leptodiaptomus ashlandi | 16 | 1.15 | Edmondson.1959 | Diaptomus ashlandi | 3 |
| Leptodiaptomus connexus | 9 | 1.20 | Edmondson.1959 | Diaptomus connexus | 3 |
| Leptodiaptomus cuauhtemoci | 3 | 1.15 | Edmondson.1959 | Diaptomus cuauhtemoci | 3 |
| Leptodiaptomus novamexicanus | 32 | 1.49 | Edmondson.1959 | Diaptomus novamexicanus | 3 |
| Leptodiaptomus nudus | 36 | 1.16 | Loewen.2016 | Leptodiaptomus nudus | 3 |
| Leptodiaptomus sicilis | 58 | 1.05 | Loewen.2016 | Leptodiaptomus sicilis | 3 |
| Leptodiaptomus siciloides | 18 | 1.15 | Edmondson.1959 | Diaptomus siciloides | 3 |
| Leptodiaptomus signicauda | 90 | 1.15 | Edmondson.1959 | Diaptomus signicauda | 3 |
| Leptodiaptomus tyrrelli | 221 | 1.24 | Loewen.2016 | Leptodiaptomus tyrrelli | 3 |
| Leptodora kindtii | 47 | 8.00 | Vijverberg.2005 | Leptodora kindtii | 4 |
| Leydigia leydigi | 3 | 0.75 | Hébert.2016; CIEE.2012 | Leydigia leydigi | 1 |
| Macrocyclops | 4 | 2.05 | Genus mean | Genus mean | 4 |
| Macrocyclops albidus | 142 | 1.50 | MacLennen.2015 | Macrocyclops albidus | 4 |
| Macrocyclops fuscus | 11 | 2.60 | Edmondson.1959 | Macrocyclops fuscus | 4 |
| Macrothrix hirsuticornis | 6 | 0.55 | Edmondson.1959 | Macrothrix hirsuticornis | 1 |
| Macrothrix laticornis | 9 | 0.47 | Hébert.2016; CIEE.2012 | Macrothrix laticornis | 1 |
| Macrothrix rosea | 2 | 0.63 | Hébert.2016; CIEE.2012 | Macrothrix rosea | 1 |
| Megacyclops viridis | 1 | 1.83 | Hébert.2016; CIEE.2012 | Megacyclops viridis | 4 |
| Microcyclops varicans | 150 | 0.59 | Reid.1992 | Microcyclops varicans | 4 |
| Moina hutchinsoni | 2 | 1.60 | Edmondson.1959 | Moina hutchinsoni | 2 |
| Onychodiaptomus sanguineus | 4 | 1.95 | MacLennan.2016 | Onychodiaptomus sanguineus | 3 |
| Orthocyclops modestus | 55 | 0.89 | MacLennan.2015 | Orthocyclops modestus | 4 |
| Paracyclops poppei | 10 | 0.80 | Edmondson.1959 | Paracyclops poppei | 4 |
| Picripleuroxus denticulatus | 11 | 0.55 | Edmondson.1959 | Picripleuroxus denticulatus | 1 |
| Picripleuroxus striatus | 2 | 0.80 | Edmondson.1959 | Picripleuroxus striatus | 1 |
| Pleuroxus | 4 | 0.625 | Genus mean | Genus mean | 1 |
| Pleuroxus aduncus | 1 | 0.60 | Edmondson.1959 | Pleuroxus aduncus | 1 |
| Pleuroxus procurvus | 2 | 0.50 | Edmondson.1959 | Pleuroxus procurvus | 1 |
| Polyphemus pediculus | 160 | 0.83 | Hébert.2016; CIEE.2012 | Polyphemus pediculus | 4 |
| Pseudosida bidentata | 2 | 1.90 | Edmondson.1959 | Pseudosida bidentata | 2 |
| Scapholeberis kingi | 73 | 0.56 | Loewen.2016 | Scapholeberis kingi | 2 |
| Senecella calanoides | 12 | 2.68 | Edmondson.1959 | Senecella calanoides | 3 |
| Sida crystallina | 30 | 2.55 | Hébert.2016; Lynch.1980 | Sida crystallina | 2 |
| Simocephalus exspinosus | 1 | 2.75 | Hébert.2016; Green.1956 | Simocephalus exspinosus | 2 |
| Simocephalus serrulatus | 4 | 1.75 | Edmondson.1959 | Simocephalus serrulatus | 2 |
| Simocephalus vetulus | 47 | 1.85 | Hébert.2016; Lynch.1980 | Simocephalus vetulus | 2 |
| Skistodiaptomus oregonensis | 28 | 1.20 | Hébert.2016; CIEE.2012 | Skistodiaptomus oregonensis | 3 |
| Skistodiaptomus pallidus | 1 | 1.20 | Hébert.2016; CIEE.2012 | Skistodiaptomus pallidus | 3 |
| Streblocerus serricaudatus | 6 | 0.50 | Edmondson.1959 | Streblocerus serricaudatus | 1 |
| Tropocyclops prasinus | 18 | 0.67 | Hébert.2016; CIEE.2012 | Tropocyclops prasinus | 4 |
| Onychodiaptomus sanguineus | 4 | 1.95 | MacLennan.2016 | Onychodiaptomus sanguineus | 3 |
| Orthocyclops modestus | 55 | 0.89 | MacLennan.2015 | Orthocyclops modestus | 4 |
| Paracyclops poppei | 10 | 0.80 | Edmondson.1959 | Paracyclops poppei | 4 |
| Picripleuroxus denticulatus | 11 | 0.55 | Edmondson.1959 | Picripleuroxus denticulatus | 1 |
| Picripleuroxus striatus | 2 | 0.80 | Edmondson.1959 | Picripleuroxus striatus | 1 |
| Pleuroxus | 4 | 0.625 | Genus mean | Genus mean | 1 |
| Pleuroxus aduncus | 1 | 0.60 | Edmondson.1959 | Pleuroxus aduncus | 1 |
| Pleuroxus procurvus | 2 | 0.50 | Edmondson.1959 | Pleuroxus procurvus | 1 |
| Polyphemus pediculus | 160 | 0.83 | Hébert.2016; CIEE.2012 | Polyphemus pediculus | 4 |
| Pseudosida bidentata | 2 | 1.90 | Edmondson.1959 | Pseudosida bidentata | 2 |
| Scapholeberis kingi | 73 | 0.56 | Loewen.2016 | Scapholeberis kingi | 2 |
| Senecella calanoides | 12 | 2.68 | Edmondson.1959 | Senecella calanoides | 3 |
| Sida crystallina | 30 | 2.55 | Hébert.2016; Lynch.1980 | Sida crystallina | 2 |
| Simocephalus exspinosus | 1 | 2.75 | Hébert.2016; Green.1956 | Simocephalus exspinosus | 2 |
| Simocephalus serrulatus | 4 | 1.75 | Edmondson.1959 | Simocephalus serrulatus | 2 |
| Simocephalus vetulus | 47 | 1.85 | Hébert.2016; Lynch.1980 | Simocephalus vetulus | 2 |
| Skistodiaptomus oregonensis | 28 | 1.20 | Hébert.2016; CIEE.2012 | Skistodiaptomus oregonensis | 3 |
| Skistodiaptomus pallidus | 1 | 1.20 | Hébert.2016; CIEE.2012 | Skistodiaptomus pallidus | 3 |
| Streblocerus serricaudatus | 6 | 0.50 | Edmondson.1959 | Streblocerus serricaudatus | 1 |
| Tropocyclops prasinus | 18 | 0.67 | Hébert.2016; CIEE.2012 | Tropocyclops prasinus | 4 |

*Note*: Site count is the number of sites where each taxon was observed; reference taxa are those for which length references refer; full references include Balcer et al. (1984) *Zooplankton of the Great Lakes: A Guide to the Identification and Ecology of the Common Crustacean Species*. Madison: University of Wisconsin Press; Barnett et al. (2007) *Freshwater Biology*, 52, 769-813; Birge. (1879) *Transactions of the Wisconsin Academy of Sciences, Arts, and Letters*, 4, 77-109; Canadian Institute of Ecology and Evolution (CIEE). (2012) *Freshwater zooplankton database*. Unpublished data; Edmondson. (1959) *Fresh-Water Biology*, New York: John Wiley & Sons, Inc.; Green. (1956) *Journal of the Zoological society of London*, 126, 173-204; Hébert et al. (2016) *Ecology*, 97, 1081-1081; Koch. (1841) *Deutschlands Crustaceen, Myriapoden und Arachniden, ein Beitrag zur deutschen Fauna*, Regensburg: F. Bubset; Loewen & Vinebrooke. (2016) *Ecology*, 97, 2740-2749; Luecke & O’Brien (1981) *Limnology and Oceanography*, 26, 454-460; Lynch. (1980) *Quarterly Review of Biology*, 55, 23-42; MacLennan et al. (2015) *Freshwater Biology*, 60, 1502-1513; MacLennan & Vinebrooke. (2016) *Hydrobiologia*, 770, 193-208; Marten & Reid. (2007) *Journal of the American Mosquito Control Association*, 23, 65-92; Neretina et al. (2018) *Journal of Natural History*, 52, 155-205; Reid. (1992) *Transactions of the American Microscopical Society*, 111, 229-254; Shing-Ming. (1970) *Transactions of the American Microscopical Society*, 89, 532-538; and Vijverberg et al. (2005) *Limnology and Oceanography*, 50, 455-464.

**Table S2.** Generalized linear multilevel/mixed effect model specifications

| **Response** | **Sample size** | **Fixed**  **effect** | **Random**  **effect** | **Formula syntax** | **Family**  **(error distribution)** | **Links** | |
| --- | --- | --- | --- | --- | --- | --- | --- |
| Species richness | 1,241 | Elevation (polynomial) | Latitudinal zones | value ~ poly(Elevation.scale, 2, raw = FALSE) +  (1 + poly(Elevation.scale, 2, raw = FALSE) \| lat_class) | negbinomial | mu = log; shape = identity | |
|  |  | Latitude (polynomial) | Elevational zones | value ~ poly(Latitude.scale, 2, raw = FALSE) +  (1 + poly(Latitude.scale, 2, raw = FALSE) \| elev_class) | negbinomial | mu = log; shape = identity | |
|  |  |  |  |  |  |  | |
| Phylogenetic richness | 1,157 | Elevation (polynomial) | Latitudinal zones | value ~ poly(Elevation.scale, 2, raw = FALSE) +  (1 + poly(Elevation.scale, 2, raw = FALSE) \| lat_class) | gamma | mu = log; shape = identity | |
|  |  | Latitude (polynomial) | Elevational zones | value ~ poly(Latitude.scale, 2, raw = FALSE) +  (1 + poly(Latitude.scale, 2, raw = FALSE) \| elev_class) | gamma | mu = log; shape = identity | |
|  |  |  |  |  |  |  | |
| Functional richness | 1,024 | Elevation (polynomial) | Latitudinal zones | value ~ poly(Elevation.scale, 2, raw = FALSE) +  (1 + poly(Elevation.scale, 2, raw = FALSE) \| lat_class) | mixture(beta, beta) | mu = logit; phi = identity;  theta = identity | |
|  |  | Latitude (polynomial) | Elevational zones | value ~ poly(Latitude.scale, 2, raw = FALSE) +  (1 + poly(Latitude.scale, 2, raw = FALSE) \| elev_class) | mixture(beta, beta) | mu = logit; phi = identity;  theta = identity | |
|  |  |  |  |  |  |  | |
| Community mean body length | 1,241 | Elevation (polynomial) | Latitudinal zones | value ~ poly(Elevation.scale, 2, raw = FALSE) +  (1 + poly(Elevation.scale, 2, raw = FALSE) \| lat_class) | gamma | mu = log; shape = identity | |
|  |  | Latitude (polynomial) | Elevational zones | value ~ poly(Latitude.scale, 2, raw = FALSE) +  (1 + poly(Latitude.scale, 2, raw = FALSE) \| elev_class) | gamma | mu = log; shape = identity | |
|  |  |  |  |  |  |  | |
| Community mean feeding guilds | 1,241 | Elevation (polynomial) | Latitudinal zones | value ~ poly(Elevation.scale, 2, raw = FALSE) +  (1 + poly(Elevation.scale, 2, raw = FALSE) \| lat_class) | gamma | mu = log; shape = identity | |
|  |  | Latitude (polynomial) | Elevational zones | value ~ poly(Latitude.scale, 2, raw = FALSE) +  (1 + poly(Latitude.scale, 2, raw = FALSE) \| elev_class) | gamma | mu = log; shape = identity | |
|  |  |  |  |  |  |  | |
| Phylogenetic richness SES | 1,157 | Elevation (polynomial) | Latitudinal zones | value ~ poly(Elevation.scale, 2, raw = FALSE) +  (1 + poly(Elevation.scale, 2, raw = FALSE) \| lat_class) | skew_normal | mu = identity; sigma = identity; alpha = identity | |
|  |  | Latitude (polynomial) | Elevational zones | value ~ poly(Latitude.scale, 2, raw = FALSE) +  (1 + poly(Latitude.scale, 2, raw = FALSE) \| elev_class) | skew_normal | mu = identity; sigma = identity; alpha = identity | |
|  |  |  |  |  |  |  | |
| Phylogenetic mean pairwise distance SES | 1,157 | Elevation (polynomial) | Latitudinal zones | value ~ poly(Elevation.scale, 2, raw = FALSE) +  (1 + poly(Elevation.scale, 2, raw = FALSE) \| lat_class) | skew_normal | mu = identity; sigma = identity; alpha = identity | |
|  |  | Latitude (polynomial) | Elevational zones | value ~ poly(Latitude.scale, 2, raw = FALSE) +  (1 + poly(Latitude.scale, 2, raw = FALSE) \| elev_class) | skew_normal | mu = identity; sigma = identity; alpha = identity | |
|  |  |  |  |  |  |  | |
| Functional richness SES | 1,024 | Elevation (polynomial) | Latitudinal zones | value ~ poly(Elevation.scale, 2, raw = FALSE) +  (1 + poly(Elevation.scale, 2, raw = FALSE) \| lat_class) | skew_normal | mu = identity; sigma = identity; alpha = identity | |
|  |  | Latitude (polynomial) | Elevational zones | value ~ poly(Latitude.scale, 2, raw = FALSE) +  (1 + poly(Latitude.scale, 2, raw = FALSE) \| elev_class) | skew_normal | mu = identity; sigma = identity; alpha = identity | |
|  |  |  |  |  |  |  | |
| Functional dispersion SES | 1,157 | Elevation (polynomial) | Latitudinal zones | value ~ poly(Elevation.scale, 2, raw = FALSE) +  (1 + poly(Elevation.scale, 2, raw = FALSE) \| lat_class) | skew_normal | mu = identity; sigma = identity; alpha = identity | |
|  |  | Latitude (polynomial) | Elevational zones | value ~ poly(Latitude.scale, 2, raw = FALSE) +  (1 + poly(Latitude.scale, 2, raw = FALSE) \| elev_class) | skew_normal | mu = identity; sigma = identity; alpha = identity | |
| Species richness | 1,241 | Mean annual temperature (polynomial) | Latitudinal zones | value ~ poly(MAT.scale, 2, raw = FALSE) +  (1 + poly(MAT.scale, 2, raw = FALSE) \| lat_class) | negbinomial | mu = log; shape = identity |  |
|  |  | Mean annual temperature (polynomial) | Elevational zones | value ~ poly(MAT.scale, 2, raw = FALSE) +  (1 + poly(MAT.scale, 2, raw = FALSE) \| elev_class) | negbinomial | mu = log; shape = identity | |
|  |  |  |  |  |  |  | |
|  | 1,241 | Mean temperature difference (polynomial) | Latitudinal zones | value ~ poly(TD.scale, 2, raw = FALSE) +  (1 + poly(TD.scale, 2, raw = FALSE) \| lat_class) | negbinomial | mu = log; shape = identity | |
|  |  | Mean temperature difference (polynomial) | Elevational zones | value ~ poly(TD.scale, 2, raw = FALSE) +  (1 + poly(TD.scale, 2, raw = FALSE) \| elev_class) | negbinomial | mu = log; shape = identity | |
|  |  |  |  |  |  |  | |
| Phylogenetic richness | 1,157 | Mean annual temperature (polynomial) | Latitudinal zones | value ~ poly(MAT.scale, 2, raw = FALSE) +  (1 + poly(MAT.scale, 2, raw = FALSE) \| lat_class) | gamma | mu = log; shape = identity |  |
|  |  | Mean annual temperature (polynomial) | Elevational zones | value ~ poly(MAT.scale, 2, raw = FALSE) +  (1 + poly(MAT.scale, 2, raw = FALSE) \| elev_class) | gamma | mu = log; shape = identity | |
|  |  |  |  |  |  |  | |
|  | 1,157 | Mean temperature difference (polynomial) | Latitudinal zones | value ~ poly(TD.scale, 2, raw = FALSE) +  (1 + poly(TD.scale, 2, raw = FALSE) \| lat_class) | gamma | mu = log; shape = identity | |
|  |  | Mean temperature difference (polynomial) | Elevational zones | value ~ poly(TD.scale, 2, raw = FALSE) +  (1 + poly(TD.scale, 2, raw = FALSE) \| elev_class) | gamma | mu = log; shape = identity | |
|  |  |  |  |  |  |  | |
| Functional richness | 1,024 | Mean annual temperature (polynomial) | Latitudinal zones | value ~ poly(MAT.scale, 2, raw = FALSE) +  (1 + poly(MAT.scale, 2, raw = FALSE) \| lat_class) | mixture(beta, beta) | mu = logit; phi = identity;  theta = identity |  |
|  |  | Mean annual temperature (polynomial) | Elevational zones | value ~ poly(MAT.scale, 2, raw = FALSE) +  (1 + poly(MAT.scale, 2, raw = FALSE) \| elev_class) | mixture(beta, beta) | mu = logit; phi = identity;  theta = identity | |
|  |  |  |  |  |  |  | |
|  | 1,024 | Mean temperature difference (polynomial) | Latitudinal zones | value ~ poly(TD.scale, 2, raw = FALSE) +  (1 + poly(TD.scale, 2, raw = FALSE) \| lat_class) | mixture(beta, beta) | mu = logit; phi = identity;  theta = identity | |
|  |  | Mean temperature difference (polynomial) | Elevational zones | value ~ poly(TD.scale, 2, raw = FALSE) +  (1 + poly(TD.scale, 2, raw = FALSE) \| elev_class) | mixture(beta, beta) | mu = logit; phi = identity;  theta = identity | |
|  |  |  |  |  |  |  | |
| Community mean body length | 1,241 | Mean annual temperature (polynomial) | Latitudinal zones | value ~ poly(MAT.scale, 2, raw = FALSE) +  (1 + poly(MAT.scale, 2, raw = FALSE) \| lat_class) | gamma | mu = log; shape = identity |  |
|  |  | Mean annual temperature (polynomial) | Elevational zones | value ~ poly(MAT.scale, 2, raw = FALSE) +  (1 + poly(MAT.scale, 2, raw = FALSE) \| elev_class) | gamma | mu = log; shape = identity | |
|  |  |  |  |  |  |  | |
|  | 1,241 | Mean temperature difference (polynomial) | Latitudinal zones | value ~ poly(TD.scale, 2, raw = FALSE) +  (1 + poly(TD.scale, 2, raw = FALSE) \| lat_class) | gamma | mu = log; shape = identity | |
|  |  | Mean temperature difference (polynomial) | Elevational zones | value ~ poly(TD.scale, 2, raw = FALSE) +  (1 + poly(TD.scale, 2, raw = FALSE) \| elev_class) | gamma | mu = log; shape = identity | |
|  |  |  |  |  |  |  | |
| Community mean feeding guilds | 1,241 | Mean annual temperature (polynomial) | Latitudinal zones | value ~ poly(MAT.scale, 2, raw = FALSE) +  (1 + poly(MAT.scale, 2, raw = FALSE) \| lat_class) | gamma | mu = log; shape = identity |  |
|  |  | Mean annual temperature (polynomial) | Elevational zones | value ~ poly(MAT.scale, 2, raw = FALSE) +  (1 + poly(MAT.scale, 2, raw = FALSE) \| elev_class) | gamma | mu = log; shape = identity | |
|  |  |  |  |  |  |  | |
|  | 1,241 | Mean temperature difference (polynomial) | Latitudinal zones | value ~ poly(TD.scale, 2, raw = FALSE) +  (1 + poly(TD.scale, 2, raw = FALSE) \| lat_class) | gamma | mu = log; shape = identity | |
|  |  | Mean temperature difference (polynomial) | Elevational zones | value ~ poly(TD.scale, 2, raw = FALSE) +  (1 + poly(TD.scale, 2, raw = FALSE) \| elev_class) | gamma | mu = log; shape = identity | |
|  |  |  |  |  |  |  | |
| Phylogenetic richness SES | 1,157 | Mean annual temperature (polynomial) | Latitudinal zones | value ~ poly(MAT.scale, 2, raw = FALSE) +  (1 + poly(MAT.scale, 2, raw = FALSE) \| lat_class) | skew_normal | mu = identity; sigma = identity; alpha = identity |  |
|  |  | Mean annual temperature (polynomial) | Elevational zones | value ~ poly(MAT.scale, 2, raw = FALSE) +  (1 + poly(MAT.scale, 2, raw = FALSE) \| elev_class) | skew_normal | mu = identity; sigma = identity; alpha = identity | |
|  |  |  |  |  |  |  | |
|  | 1,157 | Mean temperature difference (polynomial) | Latitudinal zones | value ~ poly(TD.scale, 2, raw = FALSE) +  (1 + poly(TD.scale, 2, raw = FALSE) \| lat_class) | skew_normal | mu = identity; sigma = identity; alpha = identity | |
|  |  | Mean temperature difference (polynomial) | Elevational zones | value ~ poly(TD.scale, 2, raw = FALSE) +  (1 + poly(TD.scale, 2, raw = FALSE) \| elev_class) | skew_normal | mu = identity; sigma = identity; alpha = identity | |
|  |  |  |  |  |  |  | |
| Phylogenetic mean pairwise distance SES | 1,157 | Mean annual temperature (polynomial) | Latitudinal zones | value ~ poly(MAT.scale, 2, raw = FALSE) +  (1 + poly(MAT.scale, 2, raw = FALSE) \| lat_class) | skew_normal | mu = identity; sigma = identity; alpha = identity |  |
|  |  | Mean annual temperature (polynomial) | Elevational zones | value ~ poly(MAT.scale, 2, raw = FALSE) +  (1 + poly(MAT.scale, 2, raw = FALSE) \| elev_class) | skew_normal | mu = identity; sigma = identity; alpha = identity | |
|  |  |  |  |  |  |  | |
|  | 1,157 | Mean temperature difference (polynomial) | Latitudinal zones | value ~ poly(TD.scale, 2, raw = FALSE) +  (1 + poly(TD.scale, 2, raw = FALSE) \| lat_class) | skew_normal | mu = identity; sigma = identity; alpha = identity | |
|  |  | Mean temperature difference (polynomial) | Elevational zones | value ~ poly(TD.scale, 2, raw = FALSE) +  (1 + poly(TD.scale, 2, raw = FALSE) \| elev_class) | skew_normal | mu = identity; sigma = identity; alpha = identity | |
|  |  |  |  |  |  |  | |
| Functional richness SES | 1,024 | Mean annual temperature (polynomial) | Latitudinal zones | value ~ poly(MAT.scale, 2, raw = FALSE) +  (1 + poly(MAT.scale, 2, raw = FALSE) \| lat_class) | skew_normal | mu = identity; sigma = identity; alpha = identity |  |
|  |  | Mean annual temperature (polynomial) | Elevational zones | value ~ poly(MAT.scale, 2, raw = FALSE) +  (1 + poly(MAT.scale, 2, raw = FALSE) \| elev_class) | skew_normal | mu = identity; sigma = identity; alpha = identity | |
|  |  |  |  |  |  |  | |
|  | 1,024 | Mean temperature difference (polynomial) | Latitudinal zones | value ~ poly(TD.scale, 2, raw = FALSE) +  (1 + poly(TD.scale, 2, raw = FALSE) \| lat_class) | skew_normal | mu = identity; sigma = identity; alpha = identity | |
|  |  | Mean temperature difference (polynomial) | Elevational zones | value ~ poly(TD.scale, 2, raw = FALSE) +  (1 + poly(TD.scale, 2, raw = FALSE) \| elev_class) | skew_normal | mu = identity; sigma = identity; alpha = identity | |
|  |  |  |  |  |  |  | |
| Functional dispersion SES | 1,157 | Mean annual temperature (polynomial) | Latitudinal zones | value ~ poly(MAT.scale, 2, raw = FALSE) +  (1 + poly(MAT.scale, 2, raw = FALSE) \| lat_class) | skew_normal | mu = identity; sigma = identity; alpha = identity |  |
|  |  | Mean annual temperature (polynomial) | Elevational zones | value ~ poly(MAT.scale, 2, raw = FALSE) +  (1 + poly(MAT.scale, 2, raw = FALSE) \| elev_class) | skew_normal | mu = identity; sigma = identity; alpha = identity | |
|  |  |  |  |  |  |  | |
|  | 1,157 | Mean temperature difference (polynomial) | Latitudinal zones | value ~ poly(TD.scale, 2, raw = FALSE) +  (1 + poly(TD.scale, 2, raw = FALSE) \| lat_class) | skew_normal | mu = identity; sigma = identity; alpha = identity | |
|  |  | Mean temperature difference (polynomial) | Elevational zones | value ~ poly(TD.scale, 2, raw = FALSE) +  (1 + poly(TD.scale, 2, raw = FALSE) \| elev_class) | skew_normal | mu = identity; sigma = identity; alpha = identity | |
|  |  |  |  |  |  |  | |
| Mean annual temperature (*z*-scores) | 1,241 | Elevation (polynomial) | Latitudinal zones | value ~ poly(Elevation.scale, 2, raw = FALSE) +  (1 + poly(Elevation.scale, 2, raw = FALSE) \| lat_class) | gaussian | mu = identity; sigma = identity | |
|  |  |  |  |  |  |  | |
|  | 1,241 | Latitude (polynomial) | Elevational zones | value ~ poly(Latitude.scale, 2, raw = FALSE) +  (1 + poly(Latitude.scale, 2, raw = FALSE) \| elev _class) | gaussian | mu = identity; sigma = identity | |
|  |  |  |  |  |  |  | |
| Mean temperature difference (*z*-scores) | 1,241 | Elevation (polynomial) | Latitudinal zones | value ~ poly(Elevation.scale, 2, raw = FALSE) +  (1 + poly(Elevation.scale, 2, raw = FALSE) \| lat_class) | gaussian | mu = identity; sigma = identity | |
|  |  |  |  |  |  |  | |
|  | 1,241 | Latitude (polynomial) | Elevational zones | value ~ poly(Latitude.scale, 2, raw = FALSE) +  (1 + poly(Latitude.scale, 2, raw = FALSE) \| elev_class) | gaussian | mu = identity; sigma = identity | |

*Note*: Bayesian models were fit using the Stan computational framework (Stan Development Team; https://mc-stan.org/) implemented with the ‘brms’ package (Bürkner 2017), applying weakly informative default priors (except population-level effects of class “b” were set to follow a normal distribution with mean = 0 and standard deviation = 5; and for the functional richness mixture model, centered population-level intercepts were set to follow a student’s t-distribution with different locations (μ_1_ = -0.5 and μ_2_ = 0.5) and reduced scale (σ = 0.2) to improve convergence) where error distribution and link specifications were chosen based on distributional characteristics of the response metrics.

**Table S3.** Results for traditional interaction models of biodiversity metrics regressed on elevation, latitude, and their multiplicative interaction (denoted by :).

| **Parameter** | **Estimate** | **Est.**  **Error** | **L-95%**  **Cl** | **U-95%**  **CI** | **Rhat** | **Bulk**  **ESS** | **Tail**  **ESS** |
| --- | --- | --- | --- | --- | --- | --- | --- |
| **Species richness** |  |  |  |  |  |  |  |
| Population-level effects |  |  |  |  |  |  |  |
| Intercept | 1.66* | 0.02 | 1.63 | 1.70 | 1.00 | 7067 | 5273 |
| Latitude.scale | -0.08* | 0.02 | -0.11 | -0.04 | 1.00 | 6318 | 5338 |
| Elevation.scale | -0.27* | 0.02 | -0.31 | -0.24 | 1.00 | 7149 | 5776 |
| Latitude.scale:Elevation.scale | 0.04* | 0.01 | 0.01 | 0.07 | 1.00 | 7058 | 5751 |
| Family specific parameters |  |  |  |  |  |  |  |
| Shape | 8.73* | 0.90 | 7.16 | 10.64 | 1.00 | 6353 | 4403 |
| **Phylogenetic richness** | | |  |  |  |  |  |
| Population-level effects |  |  |  |  |  |  |  |
| Intercept | 5.29* | 0.01 | 5.27 | 5.31 | 1.00 | 8578 | 4879 |
| Latitude.scale | -0.08* | 0.01 | -0.10 | -0.05 | 1.00 | 6535 | 5651 |
| Elevation.scale | -0.20* | 0.01 | -0.22 | -0.17 | 1.00 | 6618 | 5767 |
| Latitude.scale:Elevation.scale | 0.01 | 0.01 | 0.00 | 0.03 | 1.00 | 7979 | 4946 |
| Family specific parameters |  |  |  |  |  |  |  |
| Shape | 6.87* | 0.27 | 6.33 | 7.42 | 1.00 | 6991 | 5453 |
| **Functional richness** | | |  |  |  |  |  |
| Population-level effects |  |  |  |  |  |  |  |
| μ_1__Intercept | -0.71* | 0.06 | -0.83 | -0.6 | 1.00 | 12259 | 15152 |
| μ_2__Intercept | 1.32* | 0.03 | 1.27 | 1.38 | 1.00 | 24427 | 24596 |
| μ_1__Latitude.scale | 0.10* | 0.04 | 0.01 | 0.19 | 1.00 | 14156 | 18687 |
| μ_1__Elevation.scale | -0.07 | 0.04 | -0.15 | 0.02 | 1.00 | 17411 | 20118 |
| μ_1__Latitude.scale:Elevation.scale | 0.11* | 0.04 | 0.04 | 0.18 | 1.00 | 11734 | 16865 |
| μ_2__Latitude.scale | 0.25* | 0.05 | 0.17 | 0.35 | 1.00 | 7416 | 9659 |
| μ_2__Elevation.scale | 0.16* | 0.05 | 0.05 | 0.24 | 1.00 | 5858 | 5640 |
| μ_2__Latitude.scale:Elevation.scale | 0.02 | 0.07 | -0.06 | 0.2 | 1.00 | 4392 | 4804 |
| Family specific parameters |  |  |  |  |  |  |  |
| μ_1__Phi1 | 3.06* | 0.19 | 2.71 | 3.47 | 1.00 | 12350 | 16494 |
| μ_2__Phi2 | 72.34* | 15.11 | 47.62 | 106.68 | 1.00 | 13898 | 18613 |
| μ_1__Theta1 | 0.79* | 0.02 | 0.75 | 0.83 | 1.00 | 13248 | 17675 |
| μ_2__Theta2 | 0.21* | 0.02 | 0.17 | 0.25 | 1.00 | 13248 | 17675 |
| **Community mean body length** | | |  |  |  |  |  |
| Population-level effects |  |  |  |  |  |  |  |
| Intercept | 0.26* | 0.01 | 0.24 | 0.28 | 1.00 | 7907 | 5699 |
| Latitude.scale | 0.16* | 0.01 | 0.14 | 0.18 | 1.00 | 7140 | 5474 |
| Elevation.scale | 0.10* | 0.01 | 0.08 | 0.11 | 1.00 | 6757 | 5473 |
| Latitude.scale:Elevation.scale | 0.01 | 0.01 | -0.01 | 0.02 | 1.00 | 8554 | 5136 |
| Family specific parameters |  |  |  |  |  |  |  |
| Shape | 9.61* | 0.38 | 8.86 | 10.38 | 1.00 | 7432 | 5338 |
| **Community mean feeding guilds** | | |  |  |  |  |  |
| Population-level effects |  |  |  |  |  |  |  |
| Intercept | 0.95* | 0.00 | 0.94 | 0.96 | 1.00 | 7625 | 5379 |
| Latitude.scale | 0.05* | 0.01 | 0.04 | 0.06 | 1.00 | 5507 | 5295 |
| Elevation.scale | 0.01 | 0.01 | 0.00 | 0.02 | 1.00 | 6204 | 5944 |
| Latitude.scale:Elevation.scale | -0.01 | 0.00 | -0.02 | 0.00 | 1.00 | 8308 | 6080 |
| Family specific parameters |  |  |  |  |  |  |  |
| Shape | 36.73* | 1.49 | 33.83 | 39.63 | 1.00 | 5266 | 4738 |
| **Phylogenetic richness SES** | | |  |  |  |  |  |
| Population-level effects |  |  |  |  |  |  |  |
| Intercept | 0.32* | 0.03 | 0.27 | 0.37 | 1.00 | 6789 | 5041 |
| Latitude.scale | -0.18* | 0.03 | -0.24 | -0.13 | 1.00 | 5570 | 4961 |
| Elevation.scale | -0.18* | 0.03 | -0.24 | -0.13 | 1.00 | 6165 | 5815 |
| Latitude.scale:Elevation.scale | -0.05* | 0.02 | -0.09 | -0.01 | 1.00 | 6497 | 5517 |
| Family specific parameters |  |  |  |  |  |  |  |
| Sigma | 0.83* | 0.02 | 0.80 | 0.87 | 1.00 | 6952 | 5929 |
| Alpha | -0.77 | 0.51 | -1.41 | 0.53 | 1.00 | 3654 | 4765 |
| **Phylogenetic mean pairwise distance SES** | | |  |  |  |  |  |
| Population-level effects |  |  |  |  |  |  |  |
| Intercept | 0.24* | 0.03 | 0.18 | 0.29 | 1.00 | 7227 | 5509 |
| Latitude.scale | -0.15* | 0.03 | -0.21 | -0.10 | 1.00 | 6411 | 5980 |
| Elevation.scale | -0.18* | 0.03 | -0.24 | -0.12 | 1.00 | 6188 | 5742 |
| Latitude.scale:Elevation.scale | -0.07* | 0.02 | -0.11 | -0.03 | 1.00 | 7547 | 5728 |
| Family specific parameters |  |  |  |  |  |  |  |
| Sigma | 0.89* | 0.02 | 0.85 | 0.93 | 1.00 | 7620 | 5661 |
| Alpha | -1.29* | 0.25 | -1.72 | -0.76 | 1.00 | 5310 | 2261 |
| **Functional richness SES** | | |  |  |  |  |  |
| Population-level effects |  |  |  |  |  |  |  |
| Intercept | 0.11* | 0.03 | 0.05 | 0.17 | 1.00 | 7749 | 5325 |
| Latitude.scale | 0.27* | 0.03 | 0.20 | 0.33 | 1.00 | 6549 | 5548 |
| Elevation.scale | 0.38* | 0.03 | 0.31 | 0.44 | 1.00 | 7266 | 5718 |
| Latitude.scale:Elevation.scale | 0.12* | 0.03 | 0.07 | 0.18 | 1.00 | 7229 | 5571 |
| Family specific parameters |  |  |  |  |  |  |  |
| Sigma | 0.93* | 0.02 | 0.89 | 0.98 | 1.00 | 6885 | 5340 |
| Alpha | 2.27* | 0.23 | 1.84 | 2.73 | 1.00 | 7033 | 5636 |
| **Functional dispersion SES** | | |  |  |  |  |  |
| Population-level effects |  |  |  |  |  |  |  |
| Intercept | 0.12* | 0.03 | 0.06 | 0.17 | 1.00 | 7405 | 5768 |
| Latitude.scale | 0.21* | 0.03 | 0.15 | 0.27 | 1.00 | 6382 | 5390 |
| Elevation.scale | 0.20* | 0.03 | 0.14 | 0.26 | 1.00 | 6279 | 5304 |
| Latitude.scale:Elevation.scale | 0.08* | 0.02 | 0.04 | 0.13 | 1.00 | 6661 | 5719 |
| Family specific parameters |  |  |  |  |  |  |  |
| Sigma | 0.88* | 0.02 | 0.84 | 0.92 | 1.00 | 6804 | 5084 |
| Alpha | 1.25* | 0.34 | 0.38 | 1.78 | 1.00 | 4377 | 1905 |

*Note*: Family and link distributions for response metrics as per Table S2; parameters for functional richness are from mixture models and assessed for each component separately (μ_1_ and μ_2_); parameter uncertainties (est. error) are presented as standard deviations; L- and U-95% CI denote lower and upper 95% credible intervals; Bulk EES denotes bulk effective sample size produced using rank normalized draws; Tail EES denotes tail effective sample size produced by computing the minimum of effective sample sizes for 5% and 95% quantiles; and * denotes parameters with 95% credible intervals that do not include zero.**Table S4.** Summary of information criteria comparing models with and without random effects for simple linear and second-degree geographic orthogonal polynomial regression models

| **Metrics** | **Fixed effects** | **Random effects** | **WAIC (SE)** | **LOOIC (SE)** |
| --- | --- | --- | --- | --- |
| Species richness | Elevation (linear) | -- | 5,978.2 (63.0) | 5,978.2 (63.0) |
|  | Elevation (linear) | Latitudinal zones | 5,901.3 (61.5) | 5,901.3 (61.5) |
|  |  | ΔIC | 76.9 | 76.9 |
|  | Elevation (polynomial) | -- | 5,959.7 (61.9) | 5,959.7 (61.9) |
|  | Elevation (polynomial) | Latitudinal zones | **5,823.1 (59.7)** | **5,823.3 (59.7)** |
|  |  | ΔIC | 136.6 | 136.4 |
|  | Latitude (linear) | -- | 6,168.7 (62.5) | 6,168.7 (62.5) |
|  | Latitude (linear) | Elevational zones | 5,875.7 (58.9) | 5,876.0 (58.9) |
|  |  | ΔIC | 293.0 | 292.7 |
|  | Latitude (polynomial) | -- | 6,156.9 (60.6) | 6,156.9 (60.6) |
|  | Latitude (polynomial) | Elevational zones | **5,847.2 (58.1)** | **5,847.5 (58.2)** |
|  |  | ΔIC | 309.7 | 309.4 |
| Phylogenetic richness | Elevation (linear) | -- | 13,240.3 (57.6) | 13,240.3 (57.6) |
|  | Elevation (linear) | Latitudinal zones | 13,156.3 (58.2) | 13,156.4 (58.2) |
|  |  | ΔIC | 84.0 | 83.9 |
|  | Elevation (polynomial) | -- | 13,231.6 (57.8) | 13,231.6 (57.8) |
|  | Elevation (polynomial) | Latitudinal zones | **13,103.7 (57.7)** | **13,103.9 (57.7)** |
|  |  | ΔIC | 127.9 | 127.7 |
|  | Latitude (linear) | -- | 13,413.8 (57.3) | 13,413.8 (57.3) |
|  | Latitude (linear) | Elevational zones | 13,148.8 (56.9) | 13,149.0 (57.0) |
|  |  | ΔIC | 265.0 | 264.8 |
|  | Latitude (polynomial) | -- | 13,409.3 (56.6) | 13,409.3 (56.6) |
|  | Latitude (polynomial) | Elevational zones | **13,131.0 (56.8)** | **13,131.4 (56.8)** |
|  |  | ΔIC | 278.3 | 277.9 |
| Functional richness | Elevation (linear) | -- | -278.2 (25.2) | -278.2 (25.2) |
|  | Elevation (linear) | Latitudinal zones | -392.1 (33.5) | -395.0 (33.4) |
|  |  | ΔIC | 113.9 | 116.8 |
|  | Elevation (polynomial) | -- | -283.3 (24.8) | -283.1 (24.8) |
|  | Elevation (polynomial) | Latitudinal zones | **-394.9 (34.1)** | **-395.4 (34.1)** |
|  |  | ΔIC | 111.6 | 112.3 |
|  | Latitude (linear) | -- | -298.4 (27.3) | -298.4 (27.3) |
|  | Latitude (linear) | Elevational zones | **-380.5 (33.8)** | **-381.5 (33.7)** |
|  |  | ΔIC | 82.1 | 83.1 |
|  | Latitude (polynomial) | -- | -318.9 (28.5) | -318.9 (28.5) |
|  | Latitude (polynomial) | Elevational zones | -357.3 (32.5) | -359.9 (32.3) |
|  |  | ΔIC | 38.4 | 41.0 |
| Community mean body length | Elevation (linear) | -- | 1,484.4 (62.9) | 1,484.4 (62.9) |
|  | Elevation (linear) | Latitudinal zones | 1,246.1 (64.8) | 1,246.2 (64.8) |
|  |  | ΔIC | 238.3 | 238.2 |
|  | Elevation (polynomial) | -- | 1,441.4 (64.4) | 1,441.4 (64.4) |
|  | Elevation (polynomial) | Latitudinal zones | **1,183.6 (67.3)** | **1,183.9 (67.3)** |
|  |  | ΔIC | 257.8 | 257.5 |
|  | Latitude (linear) | -- | 1,356.4 (67.2) | 1,356.4 (67.2) |
|  | Latitude (linear) | Elevational zones | 1,196.1 (66.4) | 1,196.3 (66.4) |
|  |  | ΔIC | 160.3 | 160.1 |
|  | Latitude (polynomial) | -- | 1,356.8 (66.8) | 1,356.9 (66.8) |
|  | Latitude (polynomial) | Elevational zones | **1,194.8 (66.5)** | **1,195.0 (66.5)** |
|  |  | ΔIC | 162.0 | 161.9 |
| Community mean feeding | Elevation (linear) | -- | 1,516.9 (74.2) | 1,516.9 (74.2) |
| guilds | Elevation (linear) | Latitudinal zones | 1,392.6 (80.3) | 1,392.7 (80.3) |
|  |  | ΔIC | 124.3 | 124.2 |
|  | Elevation (polynomial) | -- | 1,493.0 (75.5) | 1,493.0 (75.5) |
|  | Elevation (polynomial) | Latitudinal zones | **1,365.1 (80.4)** | **1,365.4 (80.4)** |
|  |  | ΔIC | 127.9 | 127.6 |
|  | Latitude (linear) | -- | 1,415.8 (80.6) | 1,415.8 (80.6) |
|  | Latitude (linear) | Elevational zones | 1,380.6 (81.4) | 1,380.7 (81.4) |
|  |  | ΔIC | 35.2 | 35.1 |
|  | Latitude (polynomial) | -- | 1,415.4 (79.9) | 1,415.4 (79.9) |
|  | Latitude (polynomial) | Elevational zones | **1,371.4 (81.4)** | **1,371.8 (81.5)** |
|  |  | ΔIC | 44 | 43.6 |
| Phylogenetic richness SES | Elevation (linear) | -- | 2,976.6 (71.6) | 2,975.8 (71.0) |
|  | Elevation (linear) | Latitudinal zones | 2,972.4 (72.5) | 2,971.7 (71.9) |
|  |  | ΔIC | 4.2 | 4.1 |
|  | Elevation (polynomial) | -- | 2,967.5 (74.3) | 2,966.0 73.2 |
|  | Elevation (polynomial) | Latitudinal zones | **2,960.5 (74.2)** | **2,959.9 (73.7)** |
|  |  | ΔIC | 7 | 6.1 |
|  | Latitude (linear) | -- | 2,897.6 (53.0) | 2,897.6 (53.0) |
|  | Latitude (linear) | Elevational zones | 2,860.4 (52.3) | 2,860.5 (52.3) |
|  |  | ΔIC | 37.2 | 37.1 |
|  | Latitude (polynomial) | -- | 2,887.1 (53.1) | 2,887.1 (53.1) |
|  | Latitude (polynomial) | Elevational zones | **2,852.6 (52.0)** | **2,852.8 (52.0)** |
|  |  | ΔIC | 34.5 | 34.3 |
| Phylogenetic mean pairwise | Elevation (linear) | -- | 3,165.9 (75.5) | 3,166.3 (75.7) |
| Distance SES | Elevation (linear) | Latitudinal zones | 3,160.9 (76.7) | 3,163.3 (77.8) |
|  |  | ΔIC | 5 | 3 |
|  | Elevation (polynomial) | -- | 3,137.9 (76.5) | 3,138.6 (76.8) |
|  | Elevation (polynomial) | Latitudinal zones | **3,106.6 (78.9)** | **3,106.6 (78.7)** |
|  |  | ΔIC | 31.3 | 32 |
|  | Latitude (linear) | -- | 3,004.4 (51.8) | 3,004.4 (51.8) |
|  | Latitude (linear) | Elevational zones | 2,973.0 (51.5) | 2,973.2 (51.5) |
|  |  | ΔIC | 31.4 | 31.2 |
|  | Latitude (polynomial) | -- | 2,992.3 (52.2) | 2,992.3 (52.2) |
|  | Latitude (polynomial) | Elevational zones | **2,954.7 (51.5)** | **2,954.9 (51.5)** |
|  |  | ΔIC | 37.6 | 37.4 |
| Functional richness SES | Elevation (linear) | -- | 2,808.7 (54.5) | 2,808.8 (54.6) |
|  | Elevation (linear) | Latitudinal zones | 2,756.0 (55.8) | 2,756.2 (55.8) |
|  |  | ΔIC | 52.7 | 52.6 |
|  | Elevation (polynomial) | -- | 2,808.6 (54.3) | 2,808.6 (54.3) |
|  | Elevation (polynomial) | Latitudinal zones | **2,753.8 (55.7)** | **2,754.2 (55.8)** |
|  |  | ΔIC | 54.8 | 54.4 |
|  | Latitude (linear) | -- | 2,758.1 (62.5) | 2,758.1 (62.5) |
|  | Latitude (linear) | Elevational zones | **2,739.2 (64.8)** | **2,739.5 (64.9)** |
|  |  | ΔIC | 18.9 | 18.6 |
|  | Latitude (polynomial) | -- | 2,744.6 (64.9) | 2,744.6 (65.0) |
|  | Latitude (polynomial) | Elevational zones | 2,742.3 (65.2) | 2,742.5 (65.2) |
|  |  | ΔIC | 2.3 | 2.1 |
| Functional dispersion SES | Elevation (linear) | -- | 3,029.3 (47.9) | 3,029.3 (47.9) |
|  | Elevation (linear) | Latitudinal zones | **3,028.9 (48.0)** | **3,028.9 (48.0)** |
|  |  | ΔIC | 0.4 | 0.4 |
|  | Elevation (polynomial) | -- | 3,029.5 (47.5) | 3,029.5 (47.5) |
|  | Elevation (polynomial) | Latitudinal zones | 3,029.4 (47.6) | 3,029.4 (47.6) |
|  |  | ΔIC | 0.1 | 0.1 |
|  | Latitude (linear) | -- | 3,202.7 (54.1) | 3,202.7 (54.1) |
|  | Latitude (linear) | Elevational zones | 3,110.0 (57.5) | 3,110.3 (57.5) |
|  |  | ΔIC | 92.7 | 92.4 |
|  | Latitude (polynomial) | -- | 3,137.4 (57.5) | 3,137.4 (57.5) |
|  | Latitude (polynomial) | Elevational zones | **3,088.2 (57.5)** | **3,088.5 (57.5)** |
|  |  | ΔIC | 49.2 | 48.9 |

*Note*: Sets of models were compared using common response variables and seeds based on the widely applicable information criterion (WAIC) and leave-one-out cross-validation information criterion (LOOIC); ΔIC are differences in information criteria between fixed and random effect models with either simple or polynomial predictors; and bold text indicates the best fitting models overall (with or without polynomial predictors and with or without random effects).**Table S5.** Detailed results for generalized linear multilevel/mixed effect models of each biodiversity metric regressed on first- and second-degree orthogonal polynomials of elevation (a) and latitude (b)

| **Parameter** | **Estimate** | **Est.**  **Error** | **L-95%**  **Cl** | **U-95%**  **CI** | **Rhat** | **Bulk**  **ESS** | **Tail**  **ESS** |
| --- | --- | --- | --- | --- | --- | --- | --- |
| **Species richness** |  |  |  |  |  |  |  |
| (a) Elevation |  |  |  |  |  |  |  |
| Group-level effects |  |  |  |  |  |  |  |
| sd(Intercept) | 0.21* | 0.08 | 0.09 | 0.42 | 1.00 | 2332 | 4250 |
| sd(Elevation.scale β_1_) | 5.04* | 1.69 | 2.26 | 8.90 | 1.00 | 3329 | 4968 |
| sd(Elevation.scale β_2_) | 4.16* | 1.62 | 1.64 | 7.83 | 1.00 | 4126 | 4887 |
| cor(Intercept, Elevation.scale β_1_) | -0.05 | 0.33 | -0.67 | 0.59 | 1.00 | 5470 | 5797 |
| cor(Intercept, Elevation.scale β_2_) | 0.24 | 0.37 | -0.51 | 0.84 | 1.00 | 6156 | 4933 |
| cor(Elevation.scale β_1_, Elevation.scale β_2_) | 0.46 | 0.29 | -0.21 | 0.90 | 1.00 | 5755 | 6404 |
| Population-level effects |  |  |  |  |  |  |  |
| Intercept | 1.61* | 0.07 | 1.46 | 1.76 | 1.00 | 3088 | 4109 |
| Elevation.scale β_1_ | -9.10* | 2.40 | -13.53 | -4.16 | 1.00 | 2882 | 3932 |
| Elevation.scale β_2_ | -4.85* | 1.92 | -8.55 | -0.75 | 1.00 | 5095 | 4690 |
| Family specific parameters |  |  |  |  |  |  |  |
| Shape | 12.01* | 1.51 | 9.46 | 15.38 | 1.00 | 14379 | 5731 |
| (b) Latitude |  |  |  |  |  |  |  |
| Group-level effects |  |  |  |  |  |  |  |
| sd(Intercept) | 0.32* | 0.09 | 0.19 | 0.54 | 1.00 | 2291 | 4011 |
| sd(Latitude.scale β_1_) | 3.72* | 1.61 | 0.93 | 7.46 | 1.00 | 2242 | 1894 |
| sd(Latitude.scale β_2_) | 5.55* | 1.93 | 2.42 | 10.1 | 1.00 | 2500 | 3833 |
| cor(Intercept, Latitude.scale β_1_) | 0.07 | 0.39 | -0.66 | 0.77 | 1.00 | 5429 | 4415 |
| cor(Intercept, Latitude.scale β_2_) | -0.52 | 0.27 | -0.91 | 0.10 | 1.00 | 3345 | 4371 |
| cor(Latitude.scale β_1_, Latitude.scale β_2_) | -0.31 | 0.39 | -0.92 | 0.50 | 1.00 | 2226 | 3536 |
| Population-level effects |  |  |  |  |  |  |  |
| Intercept | 1.66* | 0.10 | 1.46 | 1.85 | 1.00 | 1684 | 3170 |
| Latitude.scale β_1_ | 0.68 | 1.63 | -2.60 | 3.76 | 1.00 | 3652 | 4653 |
| Latitude.scale β_2_ | -2.27 | 1.86 | -5.75 | 1.64 | 1.00 | 3654 | 5040 |
| Family specific parameters |  |  |  |  |  |  |  |
| Shape | 11.50* | 1.43 | 9.09 | 14.62 | 1.00 | 11747 | 5773 |
| **Phylogenetic richness** |  |  |  |  |  |  |  |
| (a) Elevation |  |  |  |  |  |  |  |
| Group-level effects |  |  |  |  |  |  |  |
| sd(Intercept) | 0.13* | 0.06 | 0.04 | 0.29 | 1.00 | 1758 | 3882 |
| sd(Elevation.scale β_1_) | 4.04* | 1.30 | 1.90 | 6.98 | 1.00 | 2888 | 4334 |
| sd(Elevation.scale β_2_) | 2.24* | 1.12 | 0.38 | 4.83 | 1.00 | 3094 | 2551 |
| cor(Intercept, Elevation.scale β_1_) | -0.08 | 0.34 | -0.71 | 0.57 | 1.00 | 3821 | 4479 |
| cor(Intercept, Elevation.scale β_2_) | 0.23 | 0.43 | -0.68 | 0.89 | 1.00 | 5510 | 5099 |
| cor(Elevation.scale β_1_, Elevation.scale β_2_) | 0.24 | 0.37 | -0.51 | 0.85 | 1.00 | 5456 | 5825 |
| Population-level effects |  |  |  |  |  |  |  |
| Intercept | 5.28* | 0.05 | 5.18 | 5.38 | 1.00 | 2682 | 3453 |
| Elevation.scale β_1_ | -5.93* | 1.77 | -9.28 | -2.28 | 1.00 | 2451 | 4344 |
| Elevation.scale β_2_ | -3.67* | 1.20 | -6.07 | -1.26 | 1.00 | 4552 | 4966 |
| Family specific parameters |  |  |  |  |  |  |  |
| Shape | 7.54* | 0.32 | 6.93 | 8.18 | 1.00 | 12620 | 5491 |
| (b) Latitude |  |  |  |  |  |  |  |
| Group-level effects |  |  |  |  |  |  |  |
| sd(Intercept) | 0.24* | 0.07 | 0.15 | 0.40 | 1.00 | 2804 | 4531 |
| sd(Latitude.scale β_1_) | 2.74* | 1.13 | 0.78 | 5.22 | 1.00 | 3142 | 3438 |
| sd(Latitude.scale β_2_) | 3.69* | 1.40 | 1.40 | 6.96 | 1.00 | 3122 | 3474 |
| cor(Intercept, Latitude.scale β_1_) | 0.07 | 0.40 | -0.68 | 0.79 | 1.00 | 5783 | 4668 |
| cor(Intercept, Latitude.scale β_2_) | -0.54 | 0.28 | -0.92 | 0.13 | 1.00 | 3989 | 5212 |
| cor(Latitude.scale β_1_, Latitude.scale β_2_) | -0.37 | 0.38 | -0.93 | 0.44 | 1.00 | 3360 | 4185 |
| Population-level effects |  |  |  |  |  |  |  |
| Intercept | 5.31* | 0.08 | 5.16 | 5.46 | 1.00 | 1869 | 3073 |
| Latitude.scale β_1_ | 0.15 | 1.23 | -2.50 | 2.49 | 1.00 | 4419 | 4863 |
| Latitude.scale β_2_ | -1.37 | 1.31 | -3.84 | 1.33 | 1.00 | 3854 | 4826 |
| Family specific parameters |  |  |  |  |  |  |  |
| Shape | 7.39* | 0.30 | 6.81 | 8.00 | 1.00 | 12822 | 5539 |
| **Functional richness** |  |  |  |  |  |  |  |
| (a) Elevation |  |  |  |  |  |  |  |
| Group-level effects |  |  |  |  |  |  |  |
| sd(μ_1__Intercept) | 0.21* | 0.11 | 0.06 | 0.46 | 1.30 | 10 | 42 |
| sd(μ_1__Elevation.scale β_1_) | 1.41* | 1.16 | 0.05 | 4.30 | 1.00 | 22940 | 17573 |
| sd(μ_1__Elevation.scale β_2_) | 1.71* | 1.40 | 0.06 | 5.25 | 1.00 | 18951 | 17073 |
| sd(μ_2__Intercept) | 1.44* | 0.47 | 0.79 | 2.56 | 1.28 | 11 | 45 |
| sd(μ_2__Elevation.scale β_1_) | 9.74* | 5.18 | 1.12 | 20.07 | 1.42 | 8 | 32 |
| sd(μ_2__Elevation.scale β_2_) | 3.13* | 2.22 | 0.17 | 8.52 | 1.00 | 5942 | 3343 |
| cor(μ_1__Intercept, Elevation.scale β_1_) | 0.06 | 0.50 | -0.86 | 0.90 | 1.01 | 849 | 23069 |
| cor(μ_1__Intercept, Elevation.scale β_2_) | -0.15 | 0.49 | -0.92 | 0.82 | 1.01 | 432 | 20954 |
| cor(μ_1__Elevation.scale β_1_, Elevation.scale β_2_) | 0.02 | 0.50 | -0.88 | 0.89 | 1.00 | 31105 | 26277 |
| cor(μ_2__Intercept, Elevation.scale β_1_) | -0.16 | 0.34 | -0.74 | 0.56 | 1.02 | 934 | 131 |
| cor(μ_2__Intercept, Elevation.scale β_2_) | 0.11 | 0.46 | -0.78 | 0.88 | 1.00 | 6611 | 22393 |
| cor(μ_2__Elevation.scale β_1_, Elevation.scale β_2_) | 0.01 | 0.47 | -0.87 | 0.82 | 1.07 | 35 | 209 |
| Population-level effects |  |  |  |  |  |  |  |
| μ_1__Intercept | -0.70* | 0.09 | -0.88 | -0.50 | 1.19 | 14 | 45 |
| μ_2__Intercept | 0.44 | 0.23 | -0.06 | 0.86 | 1.01 | 306 | 1068 |
| μ_1__Elevation.scale β_1_ | -3.84* | 1.81 | -7.44 | -0.30 | 1.02 | 123 | 7386 |
| μ_1__Elevation.scale β_2_ | -2.30 | 2.24 | -6.87 | 1.93 | 1.19 | 14 | 40 |
| μ_2__Elevation.scale β_1_ | 1.72 | 3.52 | -5.54 | 8.08 | 1.10 | 24 | 443 |
| μ_2__Elevation.scale β_2_ | 2.54 | 2.38 | -2.79 | 6.74 | 1.01 | 384 | 3539 |
| Family specific parameters |  |  |  |  |  |  |  |
| μ_1__Phi | 2.88* | 0.18 | 2.54 | 3.23 | 1.17 | 15 | 40 |
| μ_2__Phi | 84.22* | 21.24 | 54.37 | 135.82 | 1.12 | 21 | 123 |
| μ_1__Theta | 0.74* | 0.03 | 0.69 | 0.80 | 1.39 | 8 | 29 |
| μ_2__Theta | 0.26* | 0.03 | 0.20 | 0.31 | 1.39 | 8 | 29 |
| (b) Latitude |  |  |  |  |  |  |  |
| Group-level effects |  |  |  |  |  |  |  |
| sd(μ_1__Intercept) | 0.09 | 0.06 | 0.00 | 0.24 | 1.03 | 112 | 364 |
| sd(μ_1__Latitude.scale β_1_) | 1.59* | 1.30 | 0.06 | 4.84 | 1.01 | 10256 | 15652 |
| sd(μ_1__Latitude.scale β_2_) | 1.98* | 1.61 | 0.07 | 5.97 | 1.00 | 16266 | 15219 |
| sd(μ_2__Intercept) | 1.14* | 0.29 | 0.72 | 1.86 | 1.01 | 4621 | 11353 |
| sd(μ_2__Latitude.scale β_1_) | 3.52* | 2.39 | 0.17 | 8.95 | 1.07 | 33 | 136 |
| sd(μ_2__Latitude.scale β_2_) | 3.73* | 3.36 | 0.15 | 12.65 | 1.04 | 66 | 69 |
| cor(μ_1__Intercept, Latitude.scale β_1_) | 0.02 | 0.50 | -0.87 | 0.89 | 1.00 | 31012 | 23713 |
| cor(μ_1__Intercept, Latitude.scale β_2_) | -0.07 | 0.49 | -0.89 | 0.85 | 1.00 | 29959 | 22561 |
| cor(μ_1__Latitude.scale β_1_, Latitude.scale β_2_) | -0.04 | 0.50 | -0.89 | 0.87 | 1.00 | 27142 | 26957 |
| cor(μ_2__Intercept, Latitude.scale β_1_) | 0.29 | 0.44 | -0.68 | 0.92 | 1.05 | 50 | 1177 |
| cor(μ_2__Intercept, Latitude.scale β_2_) | -0.04 | 0.44 | -0.83 | 0.82 | 1.04 | 55 | 1137 |
| cor(μ_2__Latitude.scale β_1_, Latitude.scale β_2_) | -0.14 | 0.48 | -0.91 | 0.81 | 1.02 | 117 | 16787 |
| Population-level effects |  |  |  |  |  |  |  |
| μ_1__Intercept | -0.71* | 0.13 | -0.89 | -0.45 | 1.52 | 7 | 25 |
| μ_2__Intercept | 0.59* | 0.26 | 0.03 | 1.10 | 1.20 | 14 | 29 |
| μ_1__Elevation.scale β_1_ | 4.38* | 1.63 | 1.25 | 7.67 | 1.00 | 27137 | 17781 |
| μ_1__Elevation.scale β_2_ | -3.94* | 1.87 | -7.78 | -0.40 | 1.10 | 25 | 63 |
| μ_2__Elevation.scale β_1_ | 2.27 | 2.79 | -4.14 | 6.87 | 1.25 | 11 | 30 |
| μ_2__Elevation.scale β_2_ | -5.82* | 2.56 | -11.11 | -0.89 | 1.01 | 12561 | 12987 |
| Family specific parameters |  |  |  |  |  |  |  |
| μ_1__Phi | 2.96* | 0.38 | 2.24 | 3.53 | 1.53 | 7 | 23 |
| μ_2__Phi | 84.32* | 36.87 | 48.50 | 188.52 | 1.46 | 8 | 23 |
| μ_1__Theta | 0.74* | 0.03 | 0.69 | 0.81 | 1.41 | 8 | 24 |
| μ_2__Theta | 0.26* | 0.03 | 0.19 | 0.31 | 1.41 | 8 | 24 |
| **Community mean body length** |  |  |  |  |  |  |  |
| (a) Elevation |  |  |  |  |  |  |  |
| Group-level effects |  |  |  |  |  |  |  |
| sd(Intercept) | 0.20* | 0.08 | 0.08 | 0.38 | 1.00 | 1789 | 2712 |
| sd(Elevation.scale β_1_) | 3.20* | 1.11 | 1.57 | 5.79 | 1.00 | 2765 | 4363 |
| sd(Elevation.scale β_2_) | 2.53* | 1.11 | 0.76 | 5.08 | 1.00 | 2703 | 4324 |
| cor(Intercept, Elevation.scale β_1_) | 0.19 | 0.34 | -0.49 | 0.79 | 1.00 | 5354 | 4761 |
| cor(Intercept, Elevation.scale β_2_) | 0.03 | 0.38 | -0.66 | 0.76 | 1.00 | 7410 | 5668 |
| cor(Elevation.scale β_1_, Elevation.scale β_2_) | 0.50 | 0.31 | -0.21 | 0.93 | 1.00 | 4201 | 5354 |
| Population-level effects |  |  |  |  |  |  |  |
| Intercept | 0.23* | 0.07 | 0.10 | 0.37 | 1.00 | 2511 | 3745 |
| Elevation.scale β_1_ | 1.81 | 1.52 | -1.53 | 4.46 | 1.00 | 2131 | 3439 |
| Elevation.scale β_2_ | 2.00 | 1.26 | -0.83 | 4.15 | 1.00 | 3439 | 5016 |
| Family specific parameters |  |  |  |  |  |  |  |
| Shape | 10.35* | 0.42 | 9.57 | 11.19 | 1.00 | 14503 | 5590 |
| (b) Latitude |  |  |  |  |  |  |  |
| Group-level effects |  |  |  |  |  |  |  |
| sd(Intercept) | 0.14* | 0.04 | 0.09 | 0.24 | 1.00 | 2091 | 3509 |
| sd(Latitude.scale β_1_) | 2.73* | 1.31 | 0.41 | 5.59 | 1.00 | 1451 | 1868 |
| sd(Latitude.scale β_2_) | 1.58* | 0.99 | 0.09 | 3.85 | 1.00 | 1728 | 2489 |
| cor(Intercept, Latitude.scale β_1_) | 0.55 | 0.31 | -0.22 | 0.96 | 1.00 | 3112 | 4319 |
| cor(Intercept, Latitude.scale β_2_) | -0.12 | 0.46 | -0.90 | 0.74 | 1.00 | 3029 | 4193 |
| cor(Latitude.scale β_1_, Latitude.scale β_2_) | 0.15 | 0.49 | -0.83 | 0.89 | 1.00 | 2561 | 4465 |
| Population-level effects |  |  |  |  |  |  |  |
| Intercept | 0.27* | 0.05 | 0.18 | 0.37 | 1.00 | 1690 | 2921 |
| Latitude.scale β_1_ | 4.20* | 1.09 | 2.17 | 6.57 | 1.00 | 2608 | 3153 |
| Latitude.scale β_2_ | 0.19 | 0.86 | -1.45 | 2.02 | 1.00 | 2908 | 3310 |
| Family specific parameters |  |  |  |  |  |  |  |
| Shape | 10.27* | 0.41 | 9.48 | 11.08 | 1.00 | 10473 | 5924 |
| **Community mean feeding guilds** |  |  |  |  |  |  |  |
| (a) Elevation |  |  |  |  |  |  |  |
| Group-level effects |  |  |  |  |  |  |  |
| sd(Intercept) | 0.07* | 0.03 | 0.03 | 0.14 | 1.00 | 1945 | 3662 |
| sd(Elevation.scale β_1_) | 1.55* | 0.61 | 0.62 | 2.99 | 1.00 | 2905 | 4585 |
| sd(Elevation.scale β_2_) | 1.75* | 0.67 | 0.76 | 3.30 | 1.00 | 2873 | 4712 |
| cor(Intercept, Elevation.scale β_1_) | -0.29 | 0.36 | -0.85 | 0.49 | 1.00 | 3966 | 4506 |
| cor(Intercept, Elevation.scale β_2_) | -0.16 | 0.39 | -0.85 | 0.60 | 1.00 | 3549 | 4150 |
| cor(Elevation.scale β_1_, Elevation.scale β_2_) | 0.64 | 0.30 | -0.13 | 0.98 | 1.00 | 3215 | 4295 |
| Population-level effects |  |  |  |  |  |  |  |
| Intercept | 0.96* | 0.03 | 0.91 | 1.01 | 1.00 | 2159 | 3233 |
| Elevation.scale β_1_ | 0.10 | 0.65 | -1.26 | 1.34 | 1.00 | 2475 | 3642 |
| Elevation.scale β_2_ | 0.43 | 0.73 | -1.14 | 1.73 | 1.00 | 3228 | 4110 |
| Family specific parameters |  |  |  |  |  |  |  |
| Shape | 38.54* | 1.57 | 35.55 | 41.71 | 1.00 | 12594 | 5731 |
| (b) Latitude |  |  |  |  |  |  |  |
| Group-level effects |  |  |  |  |  |  |  |
| sd(Intercept) | 0.03* | 0.01 | 0.01 | 0.07 | 1.00 | 2603 | 4082 |
| sd(Latitude.scale β_1_) | 0.39* | 0.33 | 0.01 | 1.23 | 1.00 | 3765 | 4176 |
| sd(Latitude.scale β_2_) | 1.31* | 0.51 | 0.54 | 2.51 | 1.00 | 2550 | 3538 |
| cor(Intercept, Latitude.scale β_1_) | -0.20 | 0.48 | -0.92 | 0.78 | 1.00 | 6497 | 5462 |
| cor(Intercept, Latitude.scale β_2_) | -0.29 | 0.37 | -0.89 | 0.50 | 1.00 | 2743 | 4378 |
| cor(Latitude.scale β_1_, Latitude.scale β_2_) | 0.08 | 0.50 | -0.85 | 0.90 | 1.00 | 1403 | 3537 |
| Population-level effects |  |  |  |  |  |  |  |
| Intercept | 0.96* | 0.01 | 0.94 | 0.99 | 1.00 | 2383 | 3498 |
| Latitude.scale β_1_ | 2.20* | 0.37 | 1.48 | 2.95 | 1.00 | 3507 | 4753 |
| Latitude.scale β_2_ | 0.26 | 0.48 | -0.69 | 1.24 | 1.00 | 3601 | 4137 |
| Family specific parameters |  |  |  |  |  |  |  |
| Shape | 38.23* | 1.54 | 35.31 | 41.27 | 1.00 | 9150 | 5377 |
| **Phylogenetic richness SES** |  |  |  |  |  |  |  |
| (a) Elevation |  |  |  |  |  |  |  |
| Group-level effects |  |  |  |  |  |  |  |
| sd(Intercept) | 0.05 | 0.04 | 0.00 | 0.16 | 1.00 | 2983 | 4019 |
| sd(Elevation.scale β_1_) | 2.27* | 1.51 | 0.13 | 5.79 | 1.00 | 2678 | 2986 |
| sd(Elevation.scale β_2_) | 3.18* | 1.69 | 0.33 | 6.93 | 1.00 | 2644 | 2112 |
| cor(Intercept, Elevation.scale β_1_) | -0.04 | 0.48 | -0.88 | 0.84 | 1.00 | 3336 | 5022 |
| cor(Intercept, Elevation.scale β_2_) | -0.04 | 0.48 | -0.87 | 0.85 | 1.00 | 3470 | 4434 |
| cor(Elevation.scale β_1_, Elevation.scale β_2_) | -0.22 | 0.45 | -0.92 | 0.72 | 1.00 | 2819 | 4188 |
| Population-level effects |  |  |  |  |  |  |  |
| Intercept | 0.28* | 0.04 | 0.21 | 0.35 | 1.00 | 4660 | 4073 |
| Elevation.scale β_1_ | -3.64* | 1.46 | -6.32 | -0.56 | 1.00 | 4547 | 5075 |
| Elevation.scale β_2_ | 2.39 | 1.75 | -1.39 | 5.62 | 1.00 | 3842 | 4445 |
| Family specific parameters |  |  |  |  |  |  |  |
| Sigma | 0.86* | 0.02 | 0.83 | 0.90 | 1.00 | 11300 | 5423 |
| Alpha | -0.54 | 0.56 | -1.28 | 0.66 | 1.00 | 3599 | 6934 |
| (b) Latitude |  |  |  |  |  |  |  |
| Group-level effects |  |  |  |  |  |  |  |
| sd(Intercept) | 0.11* | 0.06 | 0.01 | 0.24 | 1.00 | 1682 | 1704 |
| sd(Latitude.scale β_1_) | 2.26* | 1.58 | 0.11 | 5.83 | 1.00 | 1533 | 3008 |
| sd(Latitude.scale β_2_) | 5.72* | 2.03 | 2.06 | 10.21 | 1.00 | 1288 | 825 |
| cor(Intercept, Latitude.scale β_1_) | 0.02 | 0.48 | -0.84 | 0.88 | 1.00 | 3981 | 4700 |
| cor(Intercept, Latitude.scale β_2_) | -0.47 | 0.37 | -0.95 | 0.42 | 1.00 | 1720 | 2107 |
| cor(Latitude.scale β_1_, Latitude.scale β_2_) | -0.23 | 0.43 | -0.90 | 0.70 | 1.00 | 2223 | 2939 |
| Population-level effects |  |  |  |  |  |  |  |
| Intercept | 0.33* | 0.05 | 0.23 | 0.43 | 1.00 | 2273 | 3063 |
| Latitude.scale β_1_ | -2.65 | 1.60 | -5.82 | 0.45 | 1.00 | 3411 | 2826 |
| Latitude.scale β_2_ | 3.18 | 1.96 | -0.56 | 7.12 | 1.00 | 3752 | 4528 |
| Family specific parameters |  |  |  |  |  |  |  |
| Sigma | 0.83* | 0.02 | 0.79 | 0.86 | 1.00 | 7717 | 5762 |
| Alpha | -1.42* | 0.24 | -1.88 | -0.94 | 1.00 | 7514 | 5346 |
| **Phylogenetic MPD SES** |  |  |  |  |  |  |  |
| (a) Elevation |  |  |  |  |  |  |  |
| Group-level effects |  |  |  |  |  |  |  |
| sd(Intercept) | 0.05 | 0.04 | 0.00 | 0.15 | 1.00 | 3346 | 3601 |
| sd(Elevation.scale β_1_) | 2.07* | 1.40 | 0.12 | 5.46 | 1.00 | 2930 | 3381 |
| sd(Elevation.scale β_2_) | 7.05* | 2.21 | 3.59 | 12.19 | 1.00 | 3696 | 5219 |
| cor(Intercept, Elevation.scale β_1_) | -0.01 | 0.50 | -0.88 | 0.88 | 1.00 | 4204 | 4747 |
| cor(Intercept, Elevation.scale β_2_) | -0.03 | 0.47 | -0.85 | 0.84 | 1.01 | 1353 | 2779 |
| cor(Elevation.scale β_1_, Elevation.scale β_2_) | -0.32 | 0.43 | -0.94 | 0.65 | 1.00 | 1944 | 2846 |
| Population-level effects |  |  |  |  |  |  |  |
| Intercept | 0.21* | 0.04 | 0.13 | 0.29 | 1.00 | 3964 | 4756 |
| Elevation.scale β_1_ | -2.05 | 1.60 | -5.04 | 1.36 | 1.00 | 4343 | 5191 |
| Elevation.scale β_2_ | 2.78 | 2.56 | -2.66 | 7.35 | 1.00 | 4302 | 4989 |
| Family specific parameters |  |  |  |  |  |  |  |
| Sigma | 0.92* | 0.02 | 0.88 | 0.96 | 1.00 | 11349 | 6582 |
| Alpha | -0.33 | 0.62 | -1.23 | 0.78 | 1.00 | 3817 | 8031 |
| (b) Latitude |  |  |  |  |  |  |  |
| Group-level effects |  |  |  |  |  |  |  |
| sd(Intercept) | 0.08 | 0.06 | 0.00 | 0.21 | 1.00 | 1907 | 2723 |
| sd(Latitude.scale β_1_) | 4.33* | 1.74 | 0.90 | 8.05 | 1.00 | 2417 | 1725 |
| sd(Latitude.scale β_2_) | 6.18* | 1.89 | 3.05 | 10.46 | 1.00 | 3767 | 4365 |
| cor(Intercept, Latitude.scale β_1_) | 0.15 | 0.45 | -0.75 | 0.90 | 1.00 | 1929 | 3454 |
| cor(Intercept, Latitude.scale β_2_) | -0.26 | 0.42 | -0.91 | 0.67 | 1.00 | 1559 | 2232 |
| cor(Latitude.scale β_1_, Latitude.scale β_2_) | -0.61 | 0.30 | -0.97 | 0.15 | 1.00 | 2797 | 2554 |
| Population-level effects |  |  |  |  |  |  |  |
| Intercept | 0.23* | 0.05 | 0.15 | 0.33 | 1.00 | 3260 | 4018 |
| Latitude.scale β_1_ | -2.06 | 1.86 | -5.63 | 1.63 | 1.00 | 3470 | 5077 |
| Latitude.scale β_2_ | 2.81 | 2.09 | -1.24 | 6.91 | 1.00 | 4518 | 5732 |
| Family specific parameters |  |  |  |  |  |  |  |
| Sigma | 0.87* | 0.02 | 0.83 | 0.91 | 1.00 | 9193 | 6256 |
| Alpha | -1.79* | 0.23 | -2.26 | -1.35 | 1.00 | 8395 | 6032 |
| **Functional richness SES** |  |  |  |  |  |  |  |
| (a) Elevation |  |  |  |  |  |  |  |
| Group-level effects |  |  |  |  |  |  |  |
| sd(Intercept) | 0.14* | 0.08 | 0.02 | 0.33 | 1.00 | 1798 | 1874 |
| sd(Elevation.scale β_1_) | 8.36* | 2.64 | 4.21 | 14.46 | 1.00 | 3103 | 4248 |
| sd(Elevation.scale β_2_) | 3.50* | 2.12 | 0.18 | 8.13 | 1.00 | 2354 | 2658 |
| cor(Intercept, Elevation.scale β_1_) | -0.25 | 0.38 | -0.89 | 0.53 | 1.00 | 1678 | 2953 |
| cor(Intercept, Elevation.scale β_2_) | 0.15 | 0.45 | -0.76 | 0.88 | 1.00 | 4279 | 4399 |
| cor(Elevation.scale β_1_, Elevation.scale β_2_) | 0.10 | 0.40 | -0.73 | 0.81 | 1.00 | 5510 | 5768 |
| Population-level effects |  |  |  |  |  |  |  |
| Intercept | 0.01 | 0.07 | -0.13 | 0.14 | 1.00 | 2972 | 3980 |
| Elevation.scale β_1_ | 2.41 | 2.82 | -3.33 | 7.76 | 1.00 | 3630 | 4655 |
| Elevation.scale β_2_ | 0.08 | 2.25 | -4.6 | 4.28 | 1.00 | 3748 | 5017 |
| Family specific parameters |  |  |  |  |  |  |  |
| Sigma | 0.93* | 0.02 | 0.88 | 0.97 | 1.00 | 8770 | 6240 |
| Alpha | 1.45* | 0.22 | 1.02 | 1.89 | 1.00 | 8525 | 6267 |
| (b) Latitude |  |  |  |  |  |  |  |
| Group-level effects |  |  |  |  |  |  |  |
| sd(Intercept) | 0.08 | 0.06 | 0.00 | 0.23 | 1.00 | 1713 | 3058 |
| sd(Latitude.scale β_1_) | 2.52* | 1.78 | 0.13 | 6.65 | 1.00 | 2181 | 3144 |
| sd(Latitude.scale β_2_) | 2.27* | 1.64 | 0.09 | 6.11 | 1.00 | 2799 | 2990 |
| cor(Intercept, Latitude.scale β_1_) | 0.09 | 0.47 | -0.82 | 0.88 | 1.00 | 4600 | 5082 |
| cor(Intercept, Latitude.scale β_2_) | -0.23 | 0.48 | -0.94 | 0.76 | 1.00 | 4066 | 5218 |
| cor(Latitude.scale β_1_, Latitude.scale β_2_) | -0.06 | 0.49 | -0.88 | 0.85 | 1.00 | 5581 | 6103 |
| Population-level effects |  |  |  |  |  |  |  |
| Intercept | 0.05 | 0.05 | -0.04 | 0.14 | 1.00 | 4253 | 4807 |
| Latitude.scale β_1_ | 7.19* | 1.56 | 4.07 | 10.27 | 1.00 | 4834 | 5192 |
| Latitude.scale β_2_ | -3.63* | 1.61 | -6.82 | -0.39 | 1.00 | 4544 | 5304 |
| Family specific parameters |  |  |  |  |  |  |  |
| Sigma | 0.94* | 0.02 | 0.90 | 0.98 | 1.00 | 7993 | 6008 |
| Alpha | 2.24* | 0.25 | 1.77 | 2.73 | 1.00 | 9720 | 5988 |
| **Functional dispersion SES** |  |  |  |  |  |  |  |
| (a) Elevation |  |  |  |  |  |  |  |
| Group-level effects |  |  |  |  |  |  |  |
| sd(Intercept) | 0.05 | 0.04 | 0.00 | 0.16 | 1.00 | 3324 | 3964 |
| sd(Elevation.scale β_1_) | 2.80* | 1.71 | 0.22 | 6.67 | 1.00 | 2618 | 3500 |
| sd(Elevation.scale β_2_) | 1.45* | 1.18 | 0.06 | 4.34 | 1.00 | 4241 | 4261 |
| cor(Intercept, Elevation.scale β_1_) | -0.05 | 0.49 | -0.90 | 0.85 | 1.00 | 4658 | 5435 |
| cor(Intercept, Elevation.scale β_2_) | -0.04 | 0.50 | -0.89 | 0.87 | 1.00 | 8248 | 5513 |
| cor(Elevation.scale β_1_, Elevation.scale β_2_) | 0.01 | 0.50 | -0.87 | 0.88 | 1.00 | 7824 | 5553 |
| Population-level effects |  |  |  |  |  |  |  |
| Intercept | 0.07 | 0.04 | -0.01 | 0.14 | 1.00 | 6793 | 5365 |
| Elevation.scale β_1_ | 1.78 | 1.59 | -1.64 | 4.58 | 1.00 | 4884 | 4811 |
| Elevation.scale β_2_ | 1.48 | 1.33 | -1.20 | 3.95 | 1.00 | 6920 | 5616 |
| Family specific parameters |  |  |  |  |  |  |  |
| Sigma | 0.90* | 0.02 | 0.86 | 0.93 | 1.00 | 13492 | 6028 |
| Alpha | 1.25* | 0.33 | 0.42 | 1.75 | 1.00 | 5699 | 2024 |
| (b) Latitude |  |  |  |  |  |  |  |
| Group-level effects |  |  |  |  |  |  |  |
| sd(Intercept) | 0.10* | 0.06 | 0.01 | 0.24 | 1.00 | 2010 | 2322 |
| sd(Latitude.scale β_1_) | 3.44* | 2.34 | 0.19 | 8.79 | 1.00 | 2152 | 3913 |
| sd(Latitude.scale β_2_) | 6.63* | 2.62 | 1.80 | 12.35 | 1.00 | 2140 | 2754 |
| cor(Intercept, Latitude.scale β_1_) | 0.25 | 0.46 | -0.75 | 0.93 | 1.00 | 3738 | 4575 |
| cor(Intercept, Latitude.scale β_2_) | -0.23 | 0.42 | -0.92 | 0.60 | 1.00 | 2013 | 3528 |
| cor(Latitude.scale β_1_, Latitude.scale β_2_) | -0.19 | 0.46 | -0.92 | 0.76 | 1.00 | 1780 | 3128 |
| Population-level effects |  |  |  |  |  |  |  |
| Intercept | 0.06 | 0.05 | -0.05 | 0.17 | 1.00 | 3340 | 4265 |
| Latitude.scale β_1_ | 8.38* | 2.19 | 4.06 | 12.59 | 1.00 | 2574 | 4545 |
| Latitude.scale β_2_ | -7.14* | 2.32 | -11.53 | -2.30 | 1.00 | 4784 | 4883 |
| Family specific parameters |  |  |  |  |  |  |  |
| Sigma | 0.92* | 0.02 | 0.88 | 0.96 | 1.00 | 10797 | 5697 |
| Alpha | 1.65* | 0.21 | 1.25 | 2.07 | 1.00 | 10469 | 5727 |

*Note*: Parameter uncertainties (est. error) are presented as standard deviations; L- and U-95% CI denote lower and upper 95% credible intervals; Bulk EES denotes bulk effective sample size produced using rank normalized draws; Tail EES denotes tail effective sample size produced by computing the minimum of effective sample sizes for 5% and 95% quantiles; random effects correspond to latitudinal zones in elevation models and elevational zones in latitude models; β_1_ and β_2_ denote slope coefficients for first- and second-degree orthogonal polynomial predictors, respectively; population-level effects are summarized by means of the posterior distributions; group-level effects are summarized by standard deviations; parameters for functional richness are from mixture models and presented for each component separately (μ_1_ and μ_2_); MPD denotes mean pairwise distance; and * denotes parameters with 95% credible intervals that do not include zero.**Table S6.** Detailed results for generalized linear multilevel/mixed effect models of biodiversity, elevation, and latitude regressed on first- and second-degree orthogonal polynomials of mean annual temperature (a) and temperature difference between the means of the warmest and coldest months (b)

| **Parameter** | **Estimate** | **Est.**  **Error** | **L-95%**  **Cl** | **U-95%**  **CI** | **Rhat** | **Bulk**  **ESS** | **Tail**  **ESS** |
| --- | --- | --- | --- | --- | --- | --- | --- |
| **(a) Mean annual temperature** |  |  |  |  |  |  |  |
| Species richness |  |  |  |  |  |  |  |
| Latitudinal zone random effects |  |  |  |  |  |  |  |
| Group-level effects |  |  |  |  |  |  |  |
| sd(Intercept) | 0.33* | 0.10 | 0.17 | 0.56 | 1.00 | 2646 | 4208 |
| sd(MAT.scale β_1_) | 8.14* | 2.82 | 3.57 | 14.68 | 1.00 | 2529 | 3341 |
| sd(MAT.scale β_2_) | 5.34* | 1.59 | 2.85 | 9.08 | 1.00 | 4314 | 5082 |
| cor(Intercept, MAT.scale β_1_) | -0.49 | 0.27 | -0.88 | 0.16 | 1.00 | 3402 | 4477 |
| cor(Intercept, MAT.scale β_2_) | -0.07 | 0.33 | -0.67 | 0.56 | 1.00 | 4402 | 4846 |
| cor(MAT.scale β_1_, MAT.scale β_2_) | -0.44 | 0.26 | -0.84 | 0.14 | 1.00 | 5129 | 5667 |
| Population-level effects |  |  |  |  |  |  |  |
| Intercept | 1.55* | 0.11 | 1.35 | 1.76 | 1.00 | 2202 | 3633 |
| MAT.scale β_1_ | 9.20* | 2.99 | 3.15 | 14.94 | 1.00 | 3569 | 4864 |
| MAT.scale β_2_ | -2.70 | 1.98 | -6.50 | 1.30 | 1.00 | 4552 | 4938 |
| Family specific parameters |  |  |  |  |  |  |  |
| Shape | 10.03* | 1.14 | 8.09 | 12.59 | 1.00 | 11809 | 5839 |
| Species richness |  |  |  |  |  |  |  |
| Elevational zone random effects |  |  |  |  |  |  |  |
| Group-level effects |  |  |  |  |  |  |  |
| sd(Intercept) | 0.26* | 0.08 | 0.15 | 0.44 | 1.00 | 2721 | 3951 |
| sd(MAT.scale β_1_) | 5.20* | 1.51 | 2.80 | 8.62 | 1.00 | 3759 | 5487 |
| sd(MAT.scale β_2_) | 6.97* | 1.83 | 4.20 | 11.24 | 1.00 | 4478 | 5756 |
| cor(Intercept, MAT.scale β_1_) | -0.57 | 0.25 | -0.92 | 0.03 | 1.00 | 3567 | 5096 |
| cor(Intercept, MAT.scale β_2_) | -0.43 | 0.27 | -0.86 | 0.15 | 1.00 | 3697 | 4206 |
| cor(MAT.scale β_1_, MAT.scale β_2_) | 0.17 | 0.29 | -0.40 | 0.69 | 1.00 | 4943 | 4786 |
| Population-level effects |  |  |  |  |  |  |  |
| Intercept | 1.66* | 0.08 | 1.50 | 1.82 | 1.00 | 2474 | 3690 |
| MAT.scale β_1_ | 1.69 | 1.73 | -1.63 | 5.24 | 1.00 | 3404 | 4376 |
| MAT.scale β_2_ | 1.95 | 2.06 | -2.03 | 6.06 | 1.00 | 3826 | 4709 |
| Family specific parameters |  |  |  |  |  |  |  |
| Shape | 12.43* | 1.62 | 9.70 | 16.15 | 1.00 | 12125 | 5437 |
| Phylogenetic richness |  |  |  |  |  |  |  |
| Latitudinal zone random effects |  |  |  |  |  |  |  |
| Group-level effects |  |  |  |  |  |  |  |
| sd(Intercept) | 0.19* | 0.07 | 0.08 | 0.34 | 1.00 | 2111 | 2509 |
| sd(MAT.scale β_1_) | 4.58* | 1.60 | 1.91 | 8.23 | 1.00 | 3360 | 3571 |
| sd(MAT.scale β_2_) | 3.51* | 1.12 | 1.72 | 6.00 | 1.00 | 4422 | 5652 |
| cor(Intercept, MAT.scale β_1_) | -0.32 | 0.34 | -0.85 | 0.43 | 1.00 | 3473 | 3797 |
| cor(Intercept, MAT.scale β_2_) | -0.09 | 0.38 | -0.81 | 0.60 | 1.00 | 2886 | 2791 |
| cor(MAT.scale β_1_, MAT.scale β_2_) | -0.39 | 0.29 | -0.84 | 0.25 | 1.00 | 4098 | 5312 |
| Population-level effects |  |  |  |  |  |  |  |
| Intercept | 5.23* | 0.06 | 5.10 | 5.36 | 1.00 | 3264 | 4377 |
| MAT.scale β_1_ | 6.86* | 2.04 | 2.71 | 10.81 | 1.00 | 2774 | 4681 |
| MAT.scale β_2_ | -2.02 | 1.37 | -4.74 | 0.69 | 1.00 | 4473 | 5096 |
| Family specific parameters |  |  |  |  |  |  |  |
| Shape | 7.04* | 0.29 | 6.49 | 7.62 | 1.00 | 12270 | 5063 |
| Phylogenetic richness |  |  |  |  |  |  |  |
| Elevational zone random effects |  |  |  |  |  |  |  |
| Group-level effects |  |  |  |  |  |  |  |
| sd(Intercept) | 0.18* | 0.06 | 0.10 | 0.34 | 1.00 | 2264 | 3792 |
| sd(MAT.scale β_1_) | 3.29* | 1.04 | 1.64 | 5.65 | 1.00 | 3335 | 4801 |
| sd(MAT.scale β_2_) | 4.39* | 1.23 | 2.49 | 7.35 | 1.00 | 3462 | 4989 |
| cor(Intercept, MAT.scale β_1_) | -0.51 | 0.29 | -0.93 | 0.18 | 1.00 | 3592 | 3751 |
| cor(Intercept, MAT.scale β_2_) | -0.29 | 0.30 | -0.80 | 0.32 | 1.00 | 3909 | 4269 |
| cor(MAT.scale β_1_, MAT.scale β_2_) | 0.32 | 0.31 | -0.32 | 0.86 | 1.00 | 3288 | 3990 |
| Population-level effects |  |  |  |  |  |  |  |
| Intercept | 5.30* | 0.06 | 5.18 | 5.42 | 1.00 | 1900 | 2843 |
| MAT.scale β_1_ | 1.54 | 1.16 | -0.67 | 3.88 | 1.00 | 3347 | 3906 |
| MAT.scale β_2_ | 1.00 | 1.44 | -1.75 | 3.96 | 1.00 | 3996 | 4691 |
| Family specific parameters |  |  |  |  |  |  |  |
| Shape | 7.51* | 0.31 | 6.92 | 8.13 | 1.00 | 11348 | 5811 |
| Functional richness |  |  |  |  |  |  |  |
| Latitudinal zone random effects |  |  |  |  |  |  |  |
| Group-level effects |  |  |  |  |  |  |  |
| sd(μ_1__Intercept) | 0.64* | 0.85 | 0.03 | 2.67 | 1.57 | 7 | 27 |
| sd(μ_1__MAT.scale β_1_) | 2.46* | 2.17 | 0.09 | 7.97 | 1.11 | 22 | 67 |
| sd(μ_1__MAT.scale β_2_) | 2.93* | 2.39 | 0.12 | 8.85 | 1.19 | 14 | 85 |
| sd(μ_2__Intercept) | 1.00* | 0.73 | 0.18 | 2.40 | 1.73 | 6 | 95 |
| sd(μ_2__MAT.scale β_1_) | 6.86* | 7.11 | 0.14 | 23.76 | 1.44 | 8 | 94 |
| sd(μ_2__MAT.scale β_2_) | 3.41* | 2.67 | 0.13 | 9.96 | 1.09 | 28 | 339 |
| cor(μ_1__Intercept, MAT.scale β_1_) | -0.02 | 0.48 | -0.87 | 0.85 | 1.03 | 89 | 2237 |
| cor(μ_1__Intercept, MAT.scale β_2_) | 0.02 | 0.46 | -0.84 | 0.84 | 1.02 | 153 | 5541 |
| cor(μ_1__MAT.scale β_1_, MAT.scale β_2_) | 0.05 | 0.50 | -0.86 | 0.89 | 1.02 | 154 | 15421 |
| cor(μ_2__Intercept, MAT.scale β_1_) | 0.08 | 0.43 | -0.79 | 0.83 | 1.05 | 101 | 150 |
| cor(μ_2__Intercept, MAT.scale β_2_) | -0.02 | 0.46 | -0.85 | 0.81 | 1.11 | 22 | 196 |
| cor(μ_2__MAT.scale β_1_, MAT.scale β_2_) | -0.05 | 0.49 | -0.88 | 0.86 | 1.02 | 217 | 1875 |
| Population-level effects |  |  |  |  |  |  |  |
| μ_1__Intercept | -0.76* | 0.20 | -1.17 | -0.49 | 1.47 | 8 | 38 |
| μ_2__Intercept | -0.04 | 0.47 | -0.68 | 0.74 | 1.83 | 6 | 35 |
| μ_1__ MAT.scale β_1_ | -0.16 | 4.17 | -10.07 | 5.82 | 1.55 | 7 | 27 |
| μ_1__ MAT.scale β_2_ | 0.57 | 2.40 | -3.64 | 5.86 | 1.17 | 15 | 35 |
| μ_2__ MAT.scale β_1_ | 0.17 | 3.87 | -7.99 | 6.58 | 1.27 | 11 | 90 |
| μ_2__ MAT.scale β_2_ | 0.99 | 4.09 | -5.36 | 8.16 | 1.60 | 7 | 91 |
| Family specific parameters |  |  |  |  |  |  |  |
| μ_1__Phi | 136.61* | 210.58 | 2.55 | 722.01 | 2.09 | 5 | 26 |
| μ_2__Phi | 46.94* | 46.62 | 2.11 | 127.82 | 2.10 | 5 | 25 |
| μ_1__Theta | 0.46* | 0.29 | 0.10 | 0.77 | 2.10 | 5 | 25 |
| μ_2__Theta | 0.54* | 0.29 | 0.23 | 0.9 | 2.10 | 5 | 25 |
| Functional richness |  |  |  |  |  |  |  |
| Elevational zone random effects |  |  |  |  |  |  |  |
| Group-level effects |  |  |  |  |  |  |  |
| sd(μ_1__Intercept) | 0.13* | 0.10 | 0.01 | 0.36 | 1.06 | 46 | 132 |
| sd(μ_1__MAT.scale β_1_) | 2.17* | 1.84 | 0.08 | 6.85 | 1.06 | 45 | 86 |
| sd(μ_1__MAT.scale β_2_) | 6.44* | 3.33 | 0.78 | 13.81 | 1.08 | 33 | 110 |
| sd(μ_2__Intercept) | 1.09* | 0.26 | 0.72 | 1.72 | 1.04 | 82 | 475 |
| sd(μ_2__MAT.scale β_1_) | 6.74* | 2.06 | 3.54 | 11.44 | 1.08 | 31 | 222 |
| sd(μ_2__MAT.scale β_2_) | 3.01* | 2.60 | 0.09 | 9.39 | 1.21 | 13 | 117 |
| cor(μ_1__Intercept, MAT.scale β_1_) | 0.01 | 0.49 | -0.86 | 0.88 | 1.01 | 15884 | 21072 |
| cor(μ_1__Intercept, MAT.scale β_2_) | -0.14 | 0.44 | -0.89 | 0.75 | 1.02 | 138 | 11549 |
| cor(μ_1__MAT.scale β_1_, MAT.scale β_2_) | 0.11 | 0.47 | -0.81 | 0.89 | 1.01 | 10139 | 16953 |
| cor(μ_2__Intercept, MAT.scale β_1_) | -0.67* | 0.24 | -0.97 | -0.05 | 1.06 | 39 | 399 |
| cor(μ_2__Intercept, MAT.scale β_2_) | -0.01 | 0.43 | -0.83 | 0.77 | 1.05 | 48 | 137 |
| cor(μ_2__MAT.scale β_1_, MAT.scale β_2_) | -0.07 | 0.44 | -0.80 | 0.82 | 1.03 | 81 | 626 |
| Population-level effects |  |  |  |  |  |  |  |
| μ_1__Intercept | -0.68* | 0.12 | -0.89 | -0.46 | 1.63 | 6 | 81 |
| μ_2__Intercept | 0.54* | 0.22 | 0.11 | 0.99 | 1.14 | 19 | 143 |
| μ_1__ MAT.scale β_1_ | -3.51* | 1.73 | -6.98 | -0.13 | 1.02 | 128 | 454 |
| μ_1__ MAT.scale β_2_ | -0.71 | 2.46 | -5.50 | 4.27 | 1.02 | 172 | 616 |
| μ_2__ MAT.scale β_1_ | -3.19 | 2.22 | -7.45 | 1.30 | 1.04 | 75 | 4173 |
| μ_2__ MAT.scale β_2_ | -0.64 | 1.90 | -4.15 | 3.51 | 1.08 | 29 | 133 |
| Family specific parameters |  |  |  |  |  |  |  |
| μ_1__Phi | 2.88* | 0.36 | 2.34 | 3.56 | 1.81 | 6 | 38 |
| μ_2__Phi | 103.09* | 46.52 | 49.32 | 211.17 | 1.64 | 6 | 76 |
| μ_1__Theta | 0.75* | 0.03 | 0.69 | 0.81 | 1.55 | 7 | 65 |
| μ_2__Theta | 0.25* | 0.03 | 0.19 | 0.31 | 1.55 | 7 | 65 |
| Community mean body length |  |  |  |  |  |  |  |
| Latitudinal zone random effects |  |  |  |  |  |  |  |
| Group-level effects |  |  |  |  |  |  |  |
| sd(Intercept) | 0.04 | 0.03 | 0.00 | 0.11 | 1.01 | 2060 | 2693 |
| sd(MAT.scale β_1_) | 3.47* | 1.18 | 1.46 | 6.04 | 1.00 | 3401 | 3599 |
| sd(MAT.scale β_2_) | 2.74* | 1.01 | 1.08 | 5.06 | 1.00 | 3122 | 3977 |
| cor(Intercept, MAT.scale β_1_) | 0.15 | 0.44 | -0.75 | 0.88 | 1.00 | 1203 | 1831 |
| cor(Intercept, MAT.scale β_2_) | 0.19 | 0.43 | -0.71 | 0.89 | 1.00 | 1731 | 3033 |
| cor(MAT.scale β_1_, MAT.scale β_2_) | -0.40 | 0.31 | -0.86 | 0.30 | 1.00 | 3877 | 5433 |
| Population-level effects |  |  |  |  |  |  |  |
| Intercept | 0.26* | 0.03 | 0.21 | 0.31 | 1.00 | 3000 | 4206 |
| MAT.scale β_1_ | -4.46* | 1.25 | -6.87 | -1.93 | 1.00 | 3936 | 5119 |
| MAT.scale β_2_ | 1.79 | 1.05 | -0.41 | 3.74 | 1.00 | 3723 | 4906 |
| Family specific parameters |  |  |  |  |  |  |  |
| Shape | 10.02* | 0.40 | 9.26 | 10.83 | 1.00 | 11683 | 6216 |
| Community mean body length |  |  |  |  |  |  |  |
| Elevational zone random effects |  |  |  |  |  |  |  |
| Group-level effects |  |  |  |  |  |  |  |
| sd(Intercept) | 0.08* | 0.03 | 0.04 | 0.15 | 1.00 | 2644 | 3985 |
| sd(MAT.scale β_1_) | 2.92* | 0.95 | 1.41 | 5.11 | 1.00 | 2844 | 4236 |
| sd(MAT.scale β_2_) | 0.78* | 0.58 | 0.03 | 2.18 | 1.00 | 2706 | 3338 |
| cor(Intercept, MAT.scale β_1_) | -0.03 | 0.34 | -0.67 | 0.61 | 1.00 | 2639 | 4191 |
| cor(Intercept, MAT.scale β_2_) | -0.17 | 0.44 | -0.89 | 0.73 | 1.00 | 6204 | 4942 |
| cor(MAT.scale β_1_, MAT.scale β_2_) | 0.16 | 0.48 | -0.79 | 0.91 | 1.00 | 6261 | 5734 |
| Population-level effects |  |  |  |  |  |  |  |
| Intercept | 0.24* | 0.03 | 0.18 | 0.30 | 1.00 | 2767 | 4484 |
| MAT.scale β_1_ | -3.80* | 1.02 | -5.85 | -1.83 | 1.00 | 3006 | 4097 |
| MAT.scale β_2_ | 0.57 | 0.53 | -0.47 | 1.64 | 1.00 | 4983 | 4595 |
| Family specific parameters |  |  |  |  |  |  |  |
| Shape | 10.03* | 0.40 | 9.26 | 10.85 | 1.00 | 10814 | 5862 |
| Community mean feeding guilds |  |  |  |  |  |  |  |
| Latitudinal zone random effects |  |  |  |  |  |  |  |
| Group-level effects |  |  |  |  |  |  |  |
| sd(Intercept) | 0.04 | 0.03 | 0.00 | 0.11 | 1.00 | 1467 | 2012 |
| sd(MAT.scale β_1_) | 2.32* | 0.71 | 1.19 | 3.97 | 1.00 | 3129 | 4823 |
| sd(MAT.scale β_2_) | 1.65* | 0.55 | 0.79 | 2.91 | 1.00 | 3326 | 4619 |
| cor(Intercept, MAT.scale β_1_) | 0.18 | 0.4 | -0.62 | 0.85 | 1.00 | 1800 | 2985 |
| cor(Intercept, MAT.scale β_2_) | 0.05 | 0.4 | -0.76 | 0.74 | 1.00 | 1924 | 2526 |
| cor(MAT.scale β_1_, MAT.scale β_2_) | -0.65* | 0.24 | -0.95 | -0.05 | 1.00 | 4274 | 5566 |
| Population-level effects |  |  |  |  |  |  |  |
| Intercept | 0.98* | 0.02 | 0.93 | 1.02 | 1.00 | 2788 | 4315 |
| MAT.scale β_1_ | -1.56 | 0.92 | -3.41 | 0.29 | 1.00 | 2009 | 3135 |
| MAT.scale β_2_ | 0.53 | 0.63 | -0.76 | 1.73 | 1.00 | 3054 | 4838 |
| Family specific parameters |  |  |  |  |  |  |  |
| Shape | 38.60* | 1.56 | 35.64 | 41.72 | 1.00 | 12176 | 5559 |
| Community mean feeding guilds |  |  |  |  |  |  |  |
| Elevational zone random effects |  |  |  |  |  |  |  |
| Group-level effects |  |  |  |  |  |  |  |
| sd(Intercept) | 0.05* | 0.02 | 0.02 | 0.09 | 1.00 | 2227 | 3755 |
| sd(MAT.scale β_1_) | 1.03* | 0.56 | 0.23 | 2.40 | 1.00 | 1738 | 3318 |
| sd(MAT.scale β_2_) | 1.43* | 0.60 | 0.39 | 2.75 | 1.00 | 2089 | 2082 |
| cor(Intercept, MAT.scale β_1_) | 0.42 | 0.33 | -0.35 | 0.92 | 1.00 | 4149 | 4950 |
| cor(Intercept, MAT.scale β_2_) | 0.03 | 0.34 | -0.65 | 0.66 | 1.00 | 2841 | 4866 |
| cor(MAT.scale β_1_, MAT.scale β_2_) | -0.10 | 0.38 | -0.77 | 0.64 | 1.00 | 2399 | 3568 |
| Population-level effects |  |  |  |  |  |  |  |
| Intercept | 0.97* | 0.02 | 0.94 | 1.01 | 1.00 | 2379 | 3941 |
| MAT.scale β_1_ | -1.51* | 0.46 | -2.28 | -0.44 | 1.00 | 2755 | 3088 |
| MAT.scale β_2_ | -0.49 | 0.52 | -1.57 | 0.50 | 1.00 | 4439 | 4592 |
| Family specific parameters |  |  |  |  |  |  |  |
| Shape | 38.39* | 1.56 | 35.43 | 41.48 | 1.00 | 10923 | 6229 |
| Phylogenetic richness SES |  |  |  |  |  |  |  |
| Latitudinal zone random effects |  |  |  |  |  |  |  |
| Group-level effects |  |  |  |  |  |  |  |
| sd(Intercept) | 0.09* | 0.05 | 0.01 | 0.22 | 1.00 | 2455 | 2406 |
| sd(MAT.scale β_1_) | 3.97* | 1.89 | 0.58 | 8.25 | 1.00 | 2342 | 2254 |
| sd(MAT.scale β_2_) | 4.05* | 1.67 | 1.10 | 7.84 | 1.00 | 3154 | 2402 |
| cor(Intercept, MAT.scale β_1_) | 0.31 | 0.43 | -0.64 | 0.93 | 1.00 | 2749 | 4025 |
| cor(Intercept, MAT.scale β_2_) | 0.02 | 0.44 | -0.80 | 0.81 | 1.00 | 2175 | 3747 |
| cor(MAT.scale β_1_, MAT.scale β_2_) | 0.28 | 0.39 | -0.55 | 0.91 | 1.00 | 3362 | 5028 |
| Population-level effects |  |  |  |  |  |  |  |
| Intercept | 0.31* | 0.05 | 0.20 | 0.42 | 1.00 | 3038 | 4327 |
| MAT.scale β_1_ | 2.33 | 2.02 | -1.76 | 6.29 | 1.00 | 3142 | 4540 |
| MAT.scale β_2_ | 1.03 | 1.79 | -2.65 | 4.37 | 1.00 | 4450 | 4369 |
| Family specific parameters |  |  |  |  |  |  |  |
| Sigma | 0.86* | 0.02 | 0.83 | 0.90 | 1.00 | 10889 | 5421 |
| Alpha | -0.39 | 0.59 | -1.21 | 0.75 | 1.00 | 4374 | 7679 |
| Phylogenetic richness SES |  |  |  |  |  |  |  |
| Elevational zone random effects |  |  |  |  |  |  |  |
| Group-level effects |  |  |  |  |  |  |  |
| sd(Intercept) | 0.04 | 0.04 | 0.00 | 0.13 | 1.00 | 4068 | 4441 |
| sd(MAT.scale β_1_) | 4.59* | 1.42 | 2.27 | 7.89 | 1.00 | 4796 | 5613 |
| sd(MAT.scale β_2_) | 2.24* | 1.34 | 0.14 | 5.18 | 1.00 | 3599 | 4453 |
| cor(Intercept, MAT.scale β_1_) | -0.02 | 0.47 | -0.86 | 0.85 | 1.00 | 1683 | 2966 |
| cor(Intercept, MAT.scale β_2_) | -0.01 | 0.49 | -0.87 | 0.86 | 1.00 | 3749 | 5304 |
| cor(MAT.scale β_1_, MAT.scale β_2_) | 0.38 | 0.40 | -0.58 | 0.93 | 1.00 | 6802 | 6568 |
| Population-level effects |  |  |  |  |  |  |  |
| Intercept | 0.29* | 0.03 | 0.22 | 0.36 | 1.00 | 7732 | 5585 |
| MAT.scale β_1_ | 4.84* | 1.62 | 1.54 | 7.96 | 1.00 | 6117 | 4678 |
| MAT.scale β_2_ | 1.13 | 1.25 | -1.28 | 3.72 | 1.00 | 8017 | 5808 |
| Family specific parameters |  |  |  |  |  |  |  |
| Sigma | 0.83* | 0.02 | 0.80 | 0.87 | 1.00 | 13286 | 5311 |
| Alpha | -1.43* | 0.25 | -1.89 | -0.95 | 1.00 | 7805 | 3204 |
| Phylogenetic MPD SES |  |  |  |  |  |  |  |
| Latitudinal zone random effects |  |  |  |  |  |  |  |
| Group-level effects |  |  |  |  |  |  |  |
| sd(Intercept) | 0.07 | 0.05 | 0.00 | 0.19 | 1.00 | 2348 | 3062 |
| sd(MAT.scale β_1_) | 3.79* | 1.82 | 0.65 | 7.81 | 1.00 | 2933 | 1890 |
| sd(MAT.scale β_2_) | 7.49* | 2.05 | 4.21 | 12.38 | 1.00 | 3837 | 5848 |
| cor(Intercept, MAT.scale β_1_) | 0.27 | 0.44 | -0.70 | 0.93 | 1.00 | 2465 | 3868 |
| cor(Intercept, MAT.scale β_2_) | 0.22 | 0.43 | -0.70 | 0.91 | 1.00 | 1389 | 2329 |
| cor(MAT.scale β_1_, MAT.scale β_2_) | 0.48 | 0.34 | -0.31 | 0.95 | 1.00 | 2443 | 3300 |
| Population-level effects |  |  |  |  |  |  |  |
| Intercept | 0.27* | 0.06 | 0.16 | 0.38 | 1.00 | 2997 | 4641 |
| MAT.scale β_1_ | 2.61 | 2.17 | -1.87 | 6.83 | 1.00 | 2957 | 4321 |
| MAT.scale β_2_ | 0.56 | 2.50 | -4.62 | 5.28 | 1.00 | 4394 | 4644 |
| Family specific parameters |  |  |  |  |  |  |  |
| Sigma | 0.92* | 0.02 | 0.88 | 0.96 | 1.00 | 8641 | 6088 |
| Alpha | -0.21 | 0.62 | -1.18 | 0.83 | 1.00 | 4372 | 7821 |
| Phylogenetic MPD SES |  |  |  |  |  |  |  |
| Elevational zone random effects |  |  |  |  |  |  |  |
| Group-level effects |  |  |  |  |  |  |  |
| sd(Intercept) | 0.05 | 0.04 | 0.00 | 0.16 | 1.00 | 2404 | 3052 |
| sd(MAT.scale β_1_) | 5.17* | 1.56 | 2.68 | 8.82 | 1.00 | 3975 | 5419 |
| sd(MAT.scale β_2_) | 3.73* | 1.5 | 0.89 | 6.98 | 1.00 | 2510 | 1692 |
| cor(Intercept, MAT.scale β_1_) | -0.06 | 0.46 | -0.88 | 0.81 | 1.00 | 1462 | 2427 |
| cor(Intercept, MAT.scale β_2_) | -0.09 | 0.48 | -0.89 | 0.82 | 1.00 | 1854 | 3473 |
| cor(MAT.scale β_1_, MAT.scale β_2_) | 0.40 | 0.33 | -0.35 | 0.90 | 1.00 | 5636 | 5066 |
| Population-level effects |  |  |  |  |  |  |  |
| Intercept | 0.22* | 0.04 | 0.15 | 0.30 | 1.00 | 5417 | 4215 |
| MAT.scale β_1_ | 4.78* | 1.78 | 1.23 | 8.22 | 1.00 | 3875 | 5041 |
| MAT.scale β_2_ | 0.55 | 1.54 | -2.48 | 3.63 | 1.00 | 5689 | 5321 |
| Family specific parameters |  |  |  |  |  |  |  |
| Sigma | 0.88* | 0.02 | 0.84 | 0.92 | 1.00 | 10447 | 5875 |
| Alpha | -1.81* | 0.23 | -2.28 | -1.36 | 1.00 | 12271 | 6523 |
| Functional richness SES |  |  |  |  |  |  |  |
| Latitudinal zone random effects |  |  |  |  |  |  |  |
| Group-level effects |  |  |  |  |  |  |  |
| sd(Intercept) | 0.18* | 0.12 | 0.01 | 0.46 | 1.00 | 1206 | 2006 |
| sd(MAT.scale β_1_) | 8.16* | 2.94 | 2.82 | 14.59 | 1.00 | 1923 | 2305 |
| sd(MAT.scale β_2_) | 7.16* | 2.80 | 2.63 | 13.61 | 1.00 | 2462 | 3196 |
| cor(Intercept, MAT.scale β_1_) | 0.25 | 0.39 | -0.58 | 0.88 | 1.00 | 1653 | 1798 |
| cor(Intercept, MAT.scale β_2_) | -0.24 | 0.40 | -0.89 | 0.57 | 1.00 | 2030 | 2963 |
| cor(MAT.scale β_1_, MAT.scale β_2_) | -0.21 | 0.33 | -0.78 | 0.46 | 1.00 | 3552 | 4122 |
| Population-level effects |  |  |  |  |  |  |  |
| Intercept | -0.07 | 0.10 | -0.25 | 0.14 | 1.00 | 2040 | 3249 |
| MAT.scale β_1_ | -3.17 | 3.41 | -9.81 | 3.47 | 1.00 | 1994 | 2885 |
| MAT.scale β_2_ | -0.74 | 2.50 | -5.71 | 4.10 | 1.00 | 4611 | 5348 |
| Family specific parameters |  |  |  |  |  |  |  |
| Sigma | 0.92* | 0.02 | 0.88 | 0.97 | 1.00 | 10926 | 6272 |
| Alpha | 1.53* | 0.21 | 1.11 | 1.96 | 1.00 | 11473 | 6368 |
| Functional richness SES |  |  |  |  |  |  |  |
| Elevational zone random effects |  |  |  |  |  |  |  |
| Group-level effects |  |  |  |  |  |  |  |
| sd(Intercept) | 0.17* | 0.08 | 0.03 | 0.35 | 1.00 | 1854 | 1477 |
| sd(MAT.scale β_1_) | 6.33* | 2.19 | 2.82 | 11.49 | 1.00 | 2817 | 3202 |
| sd(MAT.scale β_2_) | 1.84* | 1.46 | 0.07 | 5.44 | 1.00 | 3438 | 3579 |
| cor(Intercept, MAT.scale β_1_) | 0.36 | 0.35 | -0.42 | 0.91 | 1.00 | 2013 | 1955 |
| cor(Intercept, MAT.scale β_2_) | -0.27 | 0.47 | -0.94 | 0.75 | 1.00 | 6588 | 5370 |
| cor(MAT.scale β_1_, MAT.scale β_2_) | -0.11 | 0.46 | -0.89 | 0.79 | 1.00 | 7571 | 6470 |
| Population-level effects |  |  |  |  |  |  |  |
| Intercept | 0.05 | 0.07 | -0.08 | 0.19 | 1.00 | 2966 | 3681 |
| MAT.scale β_1_ | -7.80* | 2.17 | -11.89 | -3.21 | 1.00 | 3628 | 4146 |
| MAT.scale β_2_ | -1.61 | 1.40 | -4.29 | 1.21 | 1.00 | 5501 | 5257 |
| Family specific parameters |  |  |  |  |  |  |  |
| Sigma | 0.93* | 0.02 | 0.89 | 0.98 | 1.00 | 9045 | 5840 |
| Alpha | 2.29* | 0.26 | 1.81 | 2.82 | 1.00 | 8472 | 6006 |
| Functional dispersion SES |  |  |  |  |  |  |  |
| Latitudinal zone random effects |  |  |  |  |  |  |  |
| Group-level effects |  |  |  |  |  |  |  |
| sd(Intercept) | 0.06 | 0.05 | 0.00 | 0.20 | 1.00 | 2118 | 2826 |
| sd(MAT.scale β_1_) | 3.69* | 1.80 | 0.61 | 7.78 | 1.00 | 2254 | 1349 |
| sd(MAT.scale β_2_) | 2.83* | 1.92 | 0.15 | 7.37 | 1.00 | 2080 | 2342 |
| cor(Intercept, MAT.scale β_1_) | -0.09 | 0.49 | -0.89 | 0.84 | 1.00 | 2203 | 3987 |
| cor(Intercept, MAT.scale β_2_) | -0.10 | 0.50 | -0.91 | 0.84 | 1.00 | 2562 | 4183 |
| cor(MAT.scale β_1_, MAT.scale β_2_) | -0.14 | 0.45 | -0.89 | 0.76 | 1.00 | 4143 | 4672 |
| Population-level effects |  |  |  |  |  |  |  |
| Intercept | 0.05 | 0.05 | -0.04 | 0.14 | 1.00 | 3480 | 3912 |
| MAT.scale β_1_ | -1.05 | 1.77 | -4.51 | 2.44 | 1.00 | 3344 | 3926 |
| MAT.scale β_2_ | 0.56 | 1.50 | -2.52 | 3.43 | 1.00 | 5719 | 5221 |
| Family specific parameters |  |  |  |  |  |  |  |
| Sigma | 0.90* | 0.02 | 0.86 | 0.93 | 1.00 | 7933 | 5710 |
| Alpha | 1.38* | 0.25 | 0.86 | 1.82 | 1.00 | 9266 | 4677 |
| Functional dispersion SES |  |  |  |  |  |  |  |
| Elevational zone random effects |  |  |  |  |  |  |  |
| Group-level effects |  |  |  |  |  |  |  |
| sd(Intercept) | 0.19* | 0.08 | 0.08 | 0.37 | 1.00 | 2732 | 4584 |
| sd(MAT.scale β_1_) | 10.2* | 2.62 | 6.21 | 16.43 | 1.00 | 3688 | 5010 |
| sd(MAT.scale β_2_) | 5.19* | 2.12 | 1.14 | 9.69 | 1.00 | 2858 | 2244 |
| cor(Intercept, MAT.scale β_1_) | 0.09 | 0.32 | -0.56 | 0.68 | 1.00 | 2000 | 3230 |
| cor(Intercept, MAT.scale β_2_) | -0.45 | 0.34 | -0.93 | 0.33 | 1.00 | 3467 | 4509 |
| cor(MAT.scale β_1_, MAT.scale β_2_) | -0.16 | 0.31 | -0.72 | 0.45 | 1.00 | 7000 | 5905 |
| Population-level effects |  |  |  |  |  |  |  |
| Intercept | 0.06 | 0.07 | -0.09 | 0.20 | 1.00 | 3519 | 4853 |
| MAT.scale β_1_ | -6.16* | 2.85 | -11.4 | -0.31 | 1.00 | 3671 | 4684 |
| MAT.scale β_2_ | -2.31 | 2.03 | -6.20 | 1.92 | 1.00 | 4097 | 5165 |
| Family specific parameters |  |  |  |  |  |  |  |
| Sigma | 0.92* | 0.02 | 0.89 | 0.97 | 1.00 | 9953 | 6036 |
| Alpha | 1.62* | 0.22 | 1.19 | 2.05 | 1.00 | 9567 | 5712 |
| Mean annual temperature (*z*-scores) |  |  |  |  |  |  |  |
| Latitudinal zone random effects |  |  |  |  |  |  |  |
| Group-level effects |  |  |  |  |  |  |  |
| sd(Intercept) | 1.83* | 0.43 | 1.20 | 2.85 | 1.00 | 2629 | 4215 |
| sd(Elevation.scale β_1_) | 12.76* | 7.30 | 2.72 | 28.36 | 1.01 | 651 | 2161 |
| sd(Elevation.scale β_2_) | 9.36* | 4.14 | 2.83 | 18.19 | 1.01 | 688 | 1387 |
| cor(Intercept, Elevation.scale β_1_) | 0.39 | 0.38 | -0.51 | 0.89 | 1.01 | 1173 | 2097 |
| cor(Intercept, Elevation.scale β_2_) | 0.45 | 0.25 | -0.15 | 0.82 | 1.00 | 2437 | 4476 |
| cor(Elevation.scale β_1_, Elevation.scale β_2_) | 0.60 | 0.33 | -0.27 | 0.96 | 1.01 | 1010 | 1650 |
| Population-level effects |  |  |  |  |  |  |  |
| Intercept | 0.47 | 0.70 | -0.81 | 1.89 | 1.00 | 1108 | 2618 |
| Elevation.scale β_1_ | -19.90* | 5.66 | -27.67 | -7.33 | 1.00 | 851 | 3330 |
| Elevation.scale β_2_ | 1.94 | 3.04 | -3.24 | 8.57 | 1.00 | 2172 | 4415 |
| Family specific parameters |  |  |  |  |  |  |  |
| Sigma | 0.20* | 0.00 | 0.19 | 0.21 | 1.00 | 3710 | 4662 |
| Mean annual temperature (*z*-scores) |  |  |  |  |  |  |  |
| Elevational zone random effects |  |  |  |  |  |  |  |
| Group-level effects |  |  |  |  |  |  |  |
| sd(Intercept) | 1.17* | 0.31 | 0.73 | 1.93 | 1.00 | 2832 | 3779 |
| sd(Latitude.scale β_1_) | 17.93* | 8.54 | 3.98 | 35.14 | 1.01 | 695 | 1081 |
| sd(Latitude.scale β_2_) | 6.09* | 1.93 | 3.24 | 10.75 | 1.00 | 3095 | 4911 |
| cor(Intercept, Latitude.scale β_1_) | 0.04 | 0.38 | -0.68 | 0.71 | 1.00 | 1022 | 2395 |
| cor(Intercept, Latitude.scale β_2_) | -0.15 | 0.29 | -0.67 | 0.43 | 1.00 | 3829 | 5188 |
| cor(Latitude.scale β_1_, Latitude.scale β_2_) | 0.17 | 0.33 | -0.50 | 0.75 | 1.00 | 3460 | 3691 |
| Population-level effects |  |  |  |  |  |  |  |
| Intercept | 0.26 | 0.55 | -0.84 | 1.34 | 1.00 | 850 | 1882 |
| Latitude.scale β_1_ | -17.41* | 8.77 | -33.70 | -1.79 | 1.01 | 745 | 1039 |
| Latitude.scale β_2_ | -3.62 | 2.54 | -8.54 | 1.71 | 1.00 | 4286 | 4373 |
| Family specific parameters |  |  |  |  |  |  |  |
| Sigma | 0.22* | 0.00 | 0.22 | 0.23 | 1.00 | 10092 | 6086 |
| **(b) Temperature difference** |  |  |  |  |  |  |  |
| Species richness |  |  |  |  |  |  |  |
| Latitudinal zone random effects |  |  |  |  |  |  |  |
| Group-level effects |  |  |  |  |  |  |  |
| sd(Intercept) | 0.36* | 0.11 | 0.20 | 0.62 | 1.00 | 3067 | 4080 |
| sd(TD.scale β_1_) | 23.03* | 5.06 | 14.94 | 34.71 | 1.00 | 2892 | 4161 |
| sd(TD.scale β_2_) | 19.70* | 4.25 | 12.84 | 29.32 | 1.00 | 3538 | 4914 |
| cor(Intercept, TD.scale β_1_) | 0.60* | 0.20 | 0.10 | 0.88 | 1.00 | 2634 | 3305 |
| cor(Intercept, TD.scale β_2_) | 0.45 | 0.25 | -0.11 | 0.83 | 1.00 | 2735 | 3574 |
| cor(TD.scale β_1_, TD.scale β_2_) | 0.87* | 0.10 | 0.59 | 0.98 | 1.00 | 3804 | 4948 |
| Population-level effects |  |  |  |  |  |  |  |
| Intercept | 1.66* | 0.12 | 1.42 | 1.90 | 1.00 | 3147 | 3543 |
| TD.scale β_1_ | 4.95 | 4.18 | -3.12 | 13.00 | 1.00 | 5685 | 5671 |
| TD.scale β_2_ | 2.37 | 3.65 | -4.77 | 9.55 | 1.00 | 6837 | 6458 |
| Family specific parameters |  |  |  |  |  |  |  |
| Shape | 9.68* | 1.09 | 7.80 | 12.04 | 1.00 | 12501 | 5510 |
| Species richness |  |  |  |  |  |  |  |
| Elevational zone random effects |  |  |  |  |  |  |  |
| Group-level effects |  |  |  |  |  |  |  |
| sd(Intercept) | 0.42* | 0.12 | 0.25 | 0.71 | 1.00 | 2268 | 3842 |
| sd(TD.scale β_1_) | 3.13* | 1.58 | 0.45 | 6.74 | 1.00 | 2908 | 1979 |
| sd(TD.scale β_2_) | 4.78* | 1.87 | 1.90 | 9.05 | 1.00 | 2860 | 3888 |
| cor(Intercept, TD.scale β_1_) | -0.15 | 0.42 | -0.87 | 0.66 | 1.00 | 6119 | 5094 |
| cor(Intercept, TD.scale β_2_) | -0.18 | 0.33 | -0.74 | 0.53 | 1.00 | 6317 | 5760 |
| cor(TD.scale β_1_, TD.scale β_2_) | -0.29 | 0.41 | -0.92 | 0.57 | 1.00 | 2203 | 3206 |
| Population-level effects |  |  |  |  |  |  |  |
| Intercept | 1.62* | 0.13 | 1.36 | 1.88 | 1.00 | 1489 | 2355 |
| TD.scale β_1_ | -0.25 | 1.63 | -3.33 | 3.17 | 1.00 | 4264 | 4266 |
| TD.scale β_2_ | -3.05 | 1.88 | -6.70 | 0.86 | 1.00 | 4235 | 3983 |
| Family specific parameters |  |  |  |  |  |  |  |
| Shape | 12.86* | 1.74 | 9.97 | 16.83 | 1.00 | 12035 | 5325 |
| Phylogenetic richness |  |  |  |  |  |  |  |
| Latitudinal zone random effects |  |  |  |  |  |  |  |
| Group-level effects |  |  |  |  |  |  |  |
| sd(Intercept) | 0.20* | 0.06 | 0.11 | 0.36 | 1.00 | 2861 | 4252 |
| sd(TD.scale β_1_) | 13.09* | 3.20 | 7.99 | 20.4 | 1.00 | 2910 | 4127 |
| sd(TD.scale β_2_) | 12.36* | 2.90 | 7.67 | 18.89 | 1.00 | 3208 | 5210 |
| cor(Intercept, TD.scale β_1_) | 0.60* | 0.20 | 0.12 | 0.89 | 1.00 | 3311 | 4987 |
| cor(Intercept, TD.scale β_2_) | 0.42 | 0.25 | -0.16 | 0.82 | 1.00 | 3305 | 4865 |
| cor(TD.scale β_1_, TD.scale β_2_) | 0.88* | 0.10 | 0.61 | 0.99 | 1.00 | 4438 | 5555 |
| Population-level effects |  |  |  |  |  |  |  |
| Intercept | 5.36* | 0.07 | 5.21 | 5.50 | 1.00 | 3134 | 4603 |
| TD.scale β_1_ | 5.96 | 3.49 | -1.15 | 12.69 | 1.00 | 3812 | 4743 |
| TD.scale β_2_ | 3.60 | 3.10 | -2.70 | 9.50 | 1.00 | 5203 | 5425 |
| Family specific parameters |  |  |  |  |  |  |  |
| Shape | 6.67* | 0.27 | 6.15 | 7.23 | 1.00 | 12282 | 5447 |
| Phylogenetic richness |  |  |  |  |  |  |  |
| Elevational zone random effects |  |  |  |  |  |  |  |
| Group-level effects |  |  |  |  |  |  |  |
| sd(Intercept) | 0.27* | 0.08 | 0.16 | 0.47 | 1.00 | 2278 | 3690 |
| sd(TD.scale β_1_) | 2.72* | 1.18 | 0.58 | 5.43 | 1.00 | 2927 | 2137 |
| sd(TD.scale β_2_) | 3.27* | 1.55 | 0.80 | 6.93 | 1.00 | 2297 | 2508 |
| cor(Intercept, TD.scale β_1_) | -0.13 | 0.42 | -0.86 | 0.67 | 1.00 | 5548 | 5340 |
| cor(Intercept, TD.scale β_2_) | -0.25 | 0.35 | -0.79 | 0.56 | 1.00 | 6007 | 4821 |
| cor(TD.scale β_1_, TD.scale β_2_) | -0.30 | 0.41 | -0.92 | 0.56 | 1.00 | 2730 | 3238 |
| Population-level effects |  |  |  |  |  |  |  |
| Intercept | 5.29* | 0.08 | 5.12 | 5.45 | 1.00 | 1580 | 2865 |
| TD.scale β_1_ | -0.45 | 1.24 | -2.95 | 2.05 | 1.00 | 4370 | 4433 |
| TD.scale β_2_ | -1.38 | 1.36 | -3.91 | 1.47 | 1.00 | 4600 | 4316 |
| Family specific parameters |  |  |  |  |  |  |  |
| Shape | 7.48* | 0.31 | 6.88 | 8.10 | 1.00 | 13301 | 5479 |
| Functional richness |  |  |  |  |  |  |  |
| Latitudinal zone random effects |  |  |  |  |  |  |  |
| Group-level effects |  |  |  |  |  |  |  |
| sd(μ_1__Intercept) | 0.85* | 0.75 | 0.02 | 2.30 | 2.02 | 5 | 30 |
| sd(μ_1__TD.scale β_1_) | 5.16* | 6.63 | 0.12 | 25.44 | 1.14 | 19 | 35 |
| sd(μ_1__TD.scale β_2_) | 6.63* | 7.87 | 0.12 | 26.30 | 1.25 | 12 | 76 |
| sd(μ_2__Intercept) | 0.52* | 0.55 | 0.04 | 1.92 | 1.54 | 7 | 26 |
| sd(μ_2__TD.scale β_1_) | 5.77* | 4.95 | 0.19 | 17.79 | 1.06 | 45 | 1126 |
| sd(μ_2__TD.scale β_2_) | 9.78* | 13.6 | 0.13 | 47.78 | 1.54 | 7 | 35 |
| cor(μ_1__Intercept, TD.scale β_1_) | -0.10 | 0.53 | -0.92 | 0.89 | 1.19 | 14 | 59 |
| cor(μ_1__Intercept, TD.scale β_2_) | -0.15 | 0.45 | -0.89 | 0.79 | 1.04 | 61 | 137 |
| cor(μ_1__TD.scale β_1_, TD.scale β_2_) | 0.07 | 0.48 | -0.85 | 0.88 | 1.02 | 183 | 9735 |
| cor(μ_2__Intercept, TD.scale β_1_) | 0.04 | 0.52 | -0.88 | 0.90 | 1.18 | 14 | 118 |
| cor(μ_2__Intercept, TD.scale β_2_) | 0.09 | 0.44 | -0.80 | 0.89 | 1.05 | 77 | 129 |
| cor(μ_2__TD.scale β_1_, TD.scale β_2_) | -0.02 | 0.49 | -0.88 | 0.86 | 1.03 | 109 | 12102 |
| Population-level effects |  |  |  |  |  |  |  |
| μ_1__Intercept | -0.75* | 0.22 | -1.19 | -0.43 | 1.43 | 8 | 40 |
| μ_2__Intercept | -0.24 | 0.38 | -0.69 | 0.63 | 1.94 | 5 | 26 |
| μ_1__ TD.scale β_1_ | -2.32 | 4.68 | -12.27 | 5.49 | 1.25 | 12 | 93 |
| μ_1__ TD.scale β_2_ | -0.64 | 3.63 | -8.65 | 6.45 | 1.11 | 23 | 46 |
| μ_2__ TD.scale β_1_ | 0.41 | 7.09 | -16.02 | 10.08 | 1.60 | 7 | 25 |
| μ_2__ TD.scale β_2_ | -0.96 | 3.97 | -9.54 | 6.45 | 1.15 | 17 | 34 |
| Family specific parameters |  |  |  |  |  |  |  |
| μ_1__Phi | 209.35* | 298.29 | 2.50 | 1064.16 | 2.64 | 5 | 25 |
| μ_2__Phi | 27.39* | 44.52 | 2.14 | 132.88 | 2.23 | 5 | 26 |
| μ_1__Theta | 0.33* | 0.24 | 0.09 | 0.76 | 2.67 | 5 | 25 |
| μ_2__Theta | 0.67* | 0.24 | 0.24 | 0.91 | 2.67 | 5 | 25 |
| Functional richness |  |  |  |  |  |  |  |
| Elevational zone random effects |  |  |  |  |  |  |  |
| Group-level effects |  |  |  |  |  |  |  |
| sd(μ_1__Intercept) | 0.68* | 0.85 | 0.03 | 2.72 | 1.79 | 6 | 26 |
| sd(μ_1__TD.scale β_1_) | 3.57* | 3.06 | 0.13 | 11.09 | 1.06 | 46 | 106 |
| sd(μ_1__TD.scale β_2_) | 4.81* | 3.13 | 0.25 | 11.90 | 1.04 | 74 | 1018 |
| sd(μ_2__Intercept) | 0.93* | 0.42 | 0.21 | 1.77 | 1.55 | 7 | 26 |
| sd(μ_2__TD.scale β_1_) | 4.96* | 4.06 | 0.27 | 16.39 | 1.07 | 39 | 53 |
| sd(μ_2__TD.scale β_2_) | 3.91* | 3.09 | 0.16 | 11.62 | 1.06 | 46 | 184 |
| cor(μ_1__Intercept, TD.scale β_1_) | 0.04 | 0.51 | -0.87 | 0.92 | 1.13 | 20 | 76 |
| cor(μ_1__Intercept, TD.scale β_2_) | -0.06 | 0.47 | -0.90 | 0.81 | 1.09 | 27 | 65 |
| cor(μ_1__TD.scale β_1_, TD.scale β_2_) | -0.21 | 0.47 | -0.93 | 0.77 | 1.00 | 11857 | 18916 |
| cor(μ_2__Intercept, TD.scale β_1_) | 0.30 | 0.42 | -0.65 | 0.93 | 1.08 | 29 | 116 |
| cor(μ_2__Intercept, TD.scale β_2_) | -0.17 | 0.43 | -0.89 | 0.72 | 1.03 | 88 | 710 |
| cor(μ_2__TD.scale β_1_, TD.scale β_2_) | -0.19 | 0.47 | -0.92 | 0.76 | 1.01 | 334 | 20105 |
| Population-level effects |  |  |  |  |  |  |  |
| μ_1__Intercept | -0.65* | 0.13 | -0.91 | -0.41 | 1.09 | 28 | 121 |
| μ_2__Intercept | 0.31 | 0.46 | -0.57 | 0.94 | 1.58 | 7 | 27 |
| μ_1__TD.scale β_1_ | 2.20 | 3.48 | -5.28 | 8.03 | 1.40 | 8 | 26 |
| μ_1__ TD.scale β_2_ | -4.61 | 2.63 | -9.83 | 0.56 | 1.15 | 17 | 85 |
| μ_2__ TD.scale β_1_ | 1.60 | 3.31 | -4.60 | 8.47 | 1.21 | 13 | 40 |
| μ_2__ TD.scale β_2_ | -4.22 | 2.91 | -9.96 | 1.67 | 1.12 | 22 | 96 |
| Family specific parameters |  |  |  |  |  |  |  |
| μ_1__Phi | 36.43* | 61.15 | 2.48 | 189.1 | 2.24 | 5 | 21 |
| μ_2__Phi | 67.90* | 43.17 | 2.60 | 141.8 | 1.71 | 6 | 24 |
| μ_1__Theta | 0.61* | 0.25 | 0.17 | 0.80 | 1.80 | 6 | 25 |
| μ_2__Theta | 0.39* | 0.25 | 0.20 | 0.83 | 1.80 | 6 | 25 |
| Community mean body length |  |  |  |  |  |  |  |
| Latitudinal zone random effects |  |  |  |  |  |  |  |
| Group-level effects |  |  |  |  |  |  |  |
| sd(Intercept) | 0.06 | 0.05 | 0.00 | 0.18 | 1.00 | 2079 | 2439 |
| sd(TD.scale β_1_) | 8.97* | 1.98 | 5.77 | 13.42 | 1.00 | 3086 | 4546 |
| sd(TD.scale β_2_) | 6.17* | 1.63 | 3.57 | 9.80 | 1.00 | 2998 | 4468 |
| cor(Intercept, TD.scale β_1_) | -0.16 | 0.40 | -0.88 | 0.63 | 1.00 | 1092 | 1610 |
| cor(Intercept, TD.scale β_2_) | -0.29 | 0.40 | -0.92 | 0.57 | 1.00 | 1269 | 1427 |
| cor(TD.scale β_1_, TD.scale β_2_) | 0.77* | 0.16 | 0.35 | 0.97 | 1.00 | 3733 | 5638 |
| Population-level effects |  |  |  |  |  |  |  |
| Intercept | 0.23* | 0.04 | 0.13 | 0.30 | 1.00 | 1929 | 4375 |
| TD.scale β_1_ | 0.06 | 2.68 | -5.11 | 5.32 | 1.00 | 3389 | 4905 |
| TD.scale β_2_ | -2.60 | 2.12 | -6.70 | 1.72 | 1.00 | 4020 | 5509 |
| Family specific parameters |  |  |  |  |  |  |  |
| Shape | 9.74* | 0.39 | 8.99 | 10.54 | 1.00 | 16021 | 5968 |
| Community mean body length |  |  |  |  |  |  |  |
| Elevational zone random effects |  |  |  |  |  |  |  |
| Group-level effects |  |  |  |  |  |  |  |
| sd(Intercept) | 0.08* | 0.02 | 0.04 | 0.13 | 1.00 | 3721 | 5082 |
| sd(TD.scale β_1_) | 0.94* | 0.82 | 0.03 | 3.13 | 1.00 | 2870 | 4178 |
| sd(TD.scale β_2_) | 4.46* | 1.32 | 2.37 | 7.51 | 1.00 | 3191 | 4918 |
| cor(Intercept, TD.scale β_1_) | 0.11 | 0.48 | -0.82 | 0.89 | 1.00 | 6701 | 5767 |
| cor(Intercept, TD.scale β_2_) | -0.67* | 0.24 | -0.97 | -0.06 | 1.00 | 2938 | 4618 |
| cor(TD.scale β_1_, TD.scale β_2_) | -0.07 | 0.5 | -0.90 | 0.85 | 1.00 | 1817 | 4190 |
| Population-level effects |  |  |  |  |  |  |  |
| Intercept | 0.24* | 0.03 | 0.19 | 0.30 | 1.00 | 2793 | 3972 |
| TD.scale β_1_ | 4.06* | 0.72 | 2.55 | 5.43 | 1.00 | 4665 | 4009 |
| TD.scale β_2_ | -0.63 | 1.44 | -3.62 | 2.13 | 1.00 | 3415 | 4705 |
| Family specific parameters |  |  |  |  |  |  |  |
| Shape | 9.99* | 0.40 | 9.20 | 10.79 | 1.00 | 13116 | 5323 |
| Community mean feeding guilds |  |  |  |  |  |  |  |
| Latitudinal zone random effects |  |  |  |  |  |  |  |
| Group-level effects |  |  |  |  |  |  |  |
| sd(Intercept) | 0.07* | 0.03 | 0.02 | 0.15 | 1.00 | 1250 | 1289 |
| sd(TD.scale β_1_) | 1.12* | 0.83 | 0.05 | 3.13 | 1.00 | 1410 | 2238 |
| sd(TD.scale β_2_) | 0.93* | 0.77 | 0.03 | 2.85 | 1.00 | 1506 | 2777 |
| cor(Intercept, TD.scale β_1_) | -0.15 | 0.47 | -0.91 | 0.78 | 1.00 | 4622 | 4265 |
| cor(Intercept, TD.scale β_2_) | -0.22 | 0.46 | -0.91 | 0.75 | 1.00 | 5809 | 5666 |
| cor(TD.scale β_1_, TD.scale β_2_) | 0.08 | 0.50 | -0.85 | 0.90 | 1.00 | 3709 | 5260 |
| Population-level effects |  |  |  |  |  |  |  |
| Intercept | 0.97* | 0.03 | 0.91 | 1.02 | 1.00 | 2085 | 2818 |
| TD.scale β_1_ | 0.70 | 1.01 | -1.01 | 2.91 | 1.00 | 1334 | 1638 |
| TD.scale β_2_ | 0.21 | 0.63 | -0.95 | 1.65 | 1.00 | 3505 | 2532 |
| Family specific parameters |  |  |  |  |  |  |  |
| Shape | 37.37* | 1.52 | 34.53 | 40.33 | 1.00 | 12579 | 5435 |
| Community mean feeding guilds |  |  |  |  |  |  |  |
| Elevational zone random effects |  |  |  |  |  |  |  |
| Group-level effects |  |  |  |  |  |  |  |
| sd(Intercept) | 0.05* | 0.02 | 0.02 | 0.08 | 1.00 | 2664 | 4956 |
| sd(TD.scale β_1_) | 0.76* | 0.43 | 0.08 | 1.83 | 1.00 | 2917 | 2572 |
| sd(TD.scale β_2_) | 0.59* | 0.45 | 0.02 | 1.68 | 1.00 | 2387 | 3718 |
| cor(Intercept, TD.scale β_1_) | -0.54 | 0.36 | -0.97 | 0.38 | 1.00 | 5255 | 5093 |
| cor(Intercept, TD.scale β_2_) | 0.00 | 0.45 | -0.84 | 0.79 | 1.00 | 7089 | 5810 |
| cor(TD.scale β_1_, TD.scale β_2_) | 0.14 | 0.48 | -0.80 | 0.91 | 1.00 | 4630 | 5909 |
| Population-level effects |  |  |  |  |  |  |  |
| Intercept | 0.97* | 0.02 | 0.94 | 1.00 | 1.00 | 3171 | 4391 |
| TD.scale β_1_ | 1.64* | 0.44 | 0.81 | 2.54 | 1.00 | 2890 | 4066 |
| TD.scale β_2_ | -0.07 | 0.41 | -0.73 | 0.89 | 1.00 | 3368 | 3219 |
| Family specific parameters |  |  |  |  |  |  |  |
| Shape | 37.52* | 1.55 | 34.55 | 40.63 | 1.00 | 13630 | 5355 |
| Phylogenetic richness SES |  |  |  |  |  |  |  |
| Latitudinal zone random effects |  |  |  |  |  |  |  |
| Group-level effects |  |  |  |  |  |  |  |
| sd(Intercept) | 0.14* | 0.08 | 0.01 | 0.34 | 1.00 | 1847 | 2854 |
| sd(TD.scale β_1_) | 2.35* | 2.07 | 0.08 | 7.62 | 1.00 | 2787 | 3367 |
| sd(TD.scale β_2_) | 8.89* | 3.36 | 3.18 | 16.49 | 1.00 | 2238 | 2716 |
| cor(Intercept, TD.scale β_1_) | 0.06 | 0.49 | -0.86 | 0.89 | 1.00 | 8230 | 5456 |
| cor(Intercept, TD.scale β_2_) | -0.04 | 0.39 | -0.74 | 0.69 | 1.00 | 3188 | 4106 |
| cor(TD.scale β_1_, TD.scale β_2_) | -0.15 | 0.48 | -0.90 | 0.81 | 1.00 | 1734 | 2897 |
| Population-level effects |  |  |  |  |  |  |  |
| Intercept | 0.35* | 0.07 | 0.21 | 0.47 | 1.00 | 4725 | 4903 |
| TD.scale β_1_ | -0.14 | 2.33 | -4.87 | 4.36 | 1.00 | 4978 | 4480 |
| TD.scale β_2_ | 2.62 | 2.93 | -3.27 | 8.23 | 1.00 | 7004 | 5926 |
| Family specific parameters |  |  |  |  |  |  |  |
| Sigma | 0.86* | 0.02 | 0.82 | 0.89 | 1.00 | 12519 | 5536 |
| Alpha | 0.18 | 0.56 | -0.90 | 0.98 | 1.00 | 4902 | 7366 |
| Phylogenetic richness SES |  |  |  |  |  |  |  |
| Elevational zone random effects |  |  |  |  |  |  |  |
| Group-level effects |  |  |  |  |  |  |  |
| sd(Intercept) | 0.14* | 0.07 | 0.02 | 0.29 | 1.00 | 1691 | 1656 |
| sd(TD.scale β_1_) | 4.24* | 2.00 | 0.62 | 8.73 | 1.00 | 2130 | 2434 |
| sd(TD.scale β_2_) | 2.71* | 1.91 | 0.16 | 7.15 | 1.00 | 2344 | 4323 |
| cor(Intercept, TD.scale β_1_) | -0.12 | 0.43 | -0.86 | 0.72 | 1.00 | 3344 | 4421 |
| cor(Intercept, TD.scale β_2_) | -0.14 | 0.47 | -0.90 | 0.79 | 1.00 | 4043 | 4663 |
| cor(TD.scale β_1_, TD.scale β_2_) | -0.13 | 0.44 | -0.87 | 0.76 | 1.00 | 4998 | 5118 |
| Population-level effects |  |  |  |  |  |  |  |
| Intercept | 0.30* | 0.06 | 0.19 | 0.42 | 1.00 | 3730 | 4861 |
| TD.scale β_1_ | -4.42* | 2.10 | -8.68 | -0.49 | 1.00 | 3533 | 4525 |
| TD.scale β_2_ | 2.79 | 1.82 | -0.46 | 6.77 | 1.00 | 3905 | 4912 |
| Family specific parameters |  |  |  |  |  |  |  |
| Sigma | 0.83* | 0.02 | 0.80 | 0.87 | 1.00 | 10116 | 6100 |
| Alpha | -1.35* | 0.25 | -1.80 | -0.85 | 1.00 | 7314 | 3377 |
| Phylogenetic MPD SES |  |  |  |  |  |  |  |
| Latitudinal zone random effects |  |  |  |  |  |  |  |
| Group-level effects |  |  |  |  |  |  |  |
| sd(Intercept) | 0.21* | 0.10 | 0.04 | 0.44 | 1.00 | 2288 | 1987 |
| sd(TD.scale β_1_) | 2.49* | 2.19 | 0.08 | 8.27 | 1.00 | 4209 | 4028 |
| sd(TD.scale β_2_) | 13.19* | 4.23 | 6.59 | 23.08 | 1.00 | 3006 | 4801 |
| cor(Intercept, TD.scale β_1_) | -0.10 | 0.49 | -0.90 | 0.84 | 1.00 | 9166 | 5899 |
| cor(Intercept, TD.scale β_2_) | 0.05 | 0.35 | -0.62 | 0.71 | 1.00 | 2993 | 4170 |
| cor(TD.scale β1, TD.scale β2) | -0.09 | 0.48 | -0.90 | 0.84 | 1.00 | 1261 | 2460 |
| Population-level effects |  |  |  |  |  |  |  |
| Intercept | 0.30* | 0.08 | 0.13 | 0.47 | 1.00 | 4444 | 4923 |
| TD.scale β_1_ | -1.22 | 2.60 | -6.45 | 3.93 | 1.00 | 7438 | 6477 |
| TD.scale β_2_ | 2.76 | 3.52 | -4.37 | 9.43 | 1.00 | 6395 | 5615 |
| Family specific parameters |  |  |  |  |  |  |  |
| Sigma | 0.91* | 0.02 | 0.87 | 0.94 | 1.00 | 14150 | 5756 |
| Alpha | 0.10 | 0.58 | -0.99 | 0.92 | 1.00 | 4036 | 7650 |
| Phylogenetic MPD SES |  |  |  |  |  |  |  |
| Elevational zone random effects |  |  |  |  |  |  |  |
| Group-level effects |  |  |  |  |  |  |  |
| sd(Intercept) | 0.09* | 0.06 | 0.01 | 0.22 | 1.00 | 1940 | 2505 |
| sd(TD.scale β_1_) | 2.51* | 1.65 | 0.13 | 6.29 | 1.00 | 1913 | 2764 |
| sd(TD.scale β_2_) | 4.93* | 2.03 | 1.13 | 9.36 | 1.00 | 1971 | 1545 |
| cor(Intercept, TD.scale β_1_) | 0.01 | 0.48 | -0.85 | 0.87 | 1.00 | 4022 | 5462 |
| cor(Intercept, TD.scale β_2_) | -0.33 | 0.42 | -0.93 | 0.63 | 1.00 | 1866 | 2351 |
| cor(TD.scale β_1_, TD.scale β_2_) | -0.11 | 0.43 | -0.84 | 0.75 | 1.00 | 2752 | 3973 |
| Population-level effects |  |  |  |  |  |  |  |
| Intercept | 0.25* | 0.05 | 0.16 | 0.35 | 1.00 | 2880 | 4298 |
| TD.scale β_1_ | -2.68 | 2.00 | -6.96 | 0.94 | 1.00 | 2442 | 2869 |
| TD.scale β_2_ | 5.28* | 2.14 | 1.10 | 9.67 | 1.00 | 2746 | 3989 |
| Family specific parameters |  |  |  |  |  |  |  |
| Sigma | 0.87* | 0.02 | 0.83 | 0.90 | 1.00 | 9693 | 5834 |
| Alpha | -1.64* | 0.22 | -2.07 | -1.21 | 1.00 | 9678 | 5708 |
| Functional richness SES |  |  |  |  |  |  |  |
| Latitudinal zone random effects |  |  |  |  |  |  |  |
| Group-level effects |  |  |  |  |  |  |  |
| sd(Intercept) | 0.21* | 0.09 | 0.07 | 0.42 | 1.00 | 2144 | 2827 |
| sd(TD.scale β_1_) | 2.80* | 2.65 | 0.10 | 9.70 | 1.00 | 3045 | 3972 |
| sd(TD.scale β_2_) | 4.74* | 2.99 | 0.30 | 11.8 | 1.00 | 2117 | 2721 |
| cor(Intercept, TD.scale β_1_) | 0.00 | 0.49 | -0.87 | 0.87 | 1.00 | 4652 | 5296 |
| cor(Intercept, TD.scale β_2_) | 0.33 | 0.42 | -0.62 | 0.94 | 1.00 | 3449 | 4798 |
| cor(TD.scale β_1_, TD.scale β_2_) | 0.13 | 0.50 | -0.83 | 0.92 | 1.00 | 3313 | 5130 |
| Population-level effects |  |  |  |  |  |  |  |
| Intercept | -0.02 | 0.08 | -0.19 | 0.15 | 1.00 | 3405 | 4518 |
| TD.scale β_1_ | -0.66 | 2.99 | -6.73 | 4.81 | 1.00 | 2161 | 4766 |
| TD.scale β_2_ | -2.76 | 2.39 | -7.87 | 1.72 | 1.00 | 5176 | 5721 |
| Family specific parameters |  |  |  |  |  |  |  |
| Sigma | 0.96* | 0.02 | 0.92 | 1.01 | 1.00 | 9665 | 6011 |
| Alpha | 1.57* | 0.22 | 1.14 | 2.01 | 1.00 | 9619 | 5780 |
| Functional richness SES |  |  |  |  |  |  |  |
| Elevational zone random effects |  |  |  |  |  |  |  |
| Group-level effects |  |  |  |  |  |  |  |
| sd(Intercept) | 0.12* | 0.08 | 0.01 | 0.30 | 1.00 | 1379 | 1917 |
| sd(TD.scale β_1_) | 3.59* | 2.24 | 0.25 | 8.77 | 1.00 | 1806 | 2547 |
| sd(TD.scale β_2_) | 2.22* | 1.72 | 0.09 | 6.43 | 1.00 | 2478 | 3747 |
| cor(Intercept, TD.scale β_1_) | -0.11 | 0.46 | -0.88 | 0.77 | 1.00 | 3185 | 4256 |
| cor(Intercept, TD.scale β_2_) | -0.10 | 0.47 | -0.90 | 0.82 | 1.00 | 5817 | 5065 |
| cor(TD.scale β_1_, TD.scale β_2_) | -0.12 | 0.48 | -0.90 | 0.81 | 1.00 | 5513 | 5707 |
| Population-level effects |  |  |  |  |  |  |  |
| Intercept | 0.05 | 0.05 | -0.06 | 0.16 | 1.00 | 3377 | 3917 |
| TD.scale β_1_ | 5.94* | 1.96 | 2.15 | 10.1 | 1.00 | 2843 | 4453 |
| TD.scale β_2_ | -2.12 | 1.79 | -5.97 | 1.21 | 1.00 | 3392 | 4199 |
| Family specific parameters |  |  |  |  |  |  |  |
| Sigma | 0.95* | 0.02 | 0.91 | 1.00 | 1.00 | 8133 | 6011 |
| Alpha | 2.29* | 0.25 | 1.81 | 2.81 | 1.00 | 8013 | 5360 |
| Functional dispersion SES |  |  |  |  |  |  |  |
| Latitudinal zone random effects |  |  |  |  |  |  |  |
| Group-level effects |  |  |  |  |  |  |  |
| sd(Intercept) | 0.05 | 0.04 | 0.00 | 0.16 | 1.00 | 3656 | 4410 |
| sd(TD.scale β_1_) | 1.60* | 1.22 | 0.06 | 4.51 | 1.00 | 5074 | 4667 |
| sd(TD.scale β_2_) | 1.63* | 1.31 | 0.06 | 4.91 | 1.00 | 5161 | 4480 |
| cor(Intercept, TD.scale β_1_) | 0.07 | 0.51 | -0.87 | 0.91 | 1.00 | 9044 | 5159 |
| cor(Intercept, TD.scale β_2_) | -0.02 | 0.50 | -0.88 | 0.87 | 1.00 | 9204 | 5691 |
| cor(TD.scale β_1_, TD.scale β_2_) | 0.00 | 0.50 | -0.88 | 0.88 | 1.00 | 6629 | 6479 |
| Population-level effects |  |  |  |  |  |  |  |
| Intercept | 0.07 | 0.04 | -0.01 | 0.14 | 1.00 | 8150 | 6165 |
| TD.scale β_1_ | -0.46 | 1.45 | -3.28 | 2.51 | 1.00 | 6339 | 6144 |
| TD.scale β_2_ | -1.00 | 1.40 | -3.84 | 1.70 | 1.00 | 8135 | 6073 |
| Family specific parameters |  |  |  |  |  |  |  |
| Sigma | 0.90* | 0.02 | 0.87 | 0.94 | 1.00 | 16733 | 5966 |
| Alpha | 1.37* | 0.27 | 0.82 | 1.83 | 1.00 | 11633 | 3546 |
| Functional dispersion SES |  |  |  |  |  |  |  |
| Elevational zone random effects |  |  |  |  |  |  |  |
| Group-level effects |  |  |  |  |  |  |  |
| sd(Intercept) | 0.23* | 0.08 | 0.10 | 0.42 | 1.00 | 3017 | 4664 |
| sd(TD.scale β_1_) | 5.49* | 3.28 | 0.37 | 12.71 | 1.00 | 1665 | 2660 |
| sd(TD.scale β_2_) | 3.78* | 2.55 | 0.19 | 9.65 | 1.00 | 1745 | 3157 |
| cor(Intercept, TD.scale β_1_) | -0.06 | 0.38 | -0.74 | 0.68 | 1.00 | 5161 | 4975 |
| cor(Intercept, TD.scale β_2_) | 0.02 | 0.43 | -0.83 | 0.79 | 1.00 | 5045 | 4683 |
| cor(TD.scale β_1_, TD.scale β_2_) | -0.29 | 0.45 | -0.94 | 0.70 | 1.00 | 4413 | 5111 |
| Population-level effects |  |  |  |  |  |  |  |
| Intercept | 0.08 | 0.08 | -0.08 | 0.24 | 1.00 | 3085 | 4003 |
| TD.scale β_1_ | 5.40* | 2.32 | 0.82 | 10.06 | 1.00 | 5732 | 5605 |
| TD.scale β_2_ | -7.21* | 2.02 | -11.32 | -3.26 | 1.00 | 4987 | 4947 |
| Family specific parameters |  |  |  |  |  |  |  |
| Sigma | 0.93* | 0.02 | 0.89 | 0.97 | 1.00 | 11123 | 5936 |
| Alpha | 1.62* | 0.21 | 1.22 | 2.04 | 1.00 | 11886 | 5502 |
| Mean temperature difference (*z*-scores) |  |  |  |  |  |  |  |
| Latitudinal zone random effects |  |  |  |  |  |  |  |
| Group-level effects |  |  |  |  |  |  |  |
| sd(Intercept) | 1.04* | 0.27 | 0.66 | 1.67 | 1.00 | 2340 | 3420 |
| sd(Elevation.scale β_1_) | 9.02* | 3.89 | 2.26 | 17.66 | 1.00 | 2532 | 2816 |
| sd(Elevation.scale β_2_) | 7.36* | 2.01 | 4.33 | 12.13 | 1.00 | 3678 | 4722 |
| cor(Intercept, Elevation.scale β_1_) | -0.63* | 0.22 | -0.90 | -0.06 | 1.00 | 4263 | 4532 |
| cor(Intercept, Elevation.scale β_2_) | -0.14 | 0.33 | -0.75 | 0.51 | 1.00 | 3572 | 5124 |
| cor(Elevation.scale β_1_, Elevation.scale β_2_) | -0.29 | 0.29 | -0.79 | 0.32 | 1.00 | 5971 | 5816 |
| Population-level effects |  |  |  |  |  |  |  |
| Intercept | -0.08 | 0.31 | -0.65 | 0.57 | 1.00 | 1754 | 2727 |
| Elevation.scale β_1_ | -1.71 | 2.70 | -7.50 | 3.15 | 1.00 | 3586 | 5092 |
| Elevation.scale _β2_ | -3.63 | 2.35 | -7.93 | 1.37 | 1.00 | 4152 | 4928 |
| Family specific parameters |  |  |  |  |  |  |  |
| Sigma | 0.32* | 0.01 | 0.30 | 0.33 | 1.00 | 12261 | 5811 |
| Mean temperature difference (*z*-scores) |  |  |  |  |  |  |  |
| Elevational zone random effects |  |  |  |  |  |  |  |
| Group-level effects |  |  |  |  |  |  |  |
| sd(Intercept) | 0.26* | 0.10 | 0.13 | 0.50 | 1.00 | 1985 | 3051 |
| sd(Latitude.scale β_1_) | 25.05* | 6.43 | 14.87 | 39.95 | 1.00 | 3799 | 4855 |
| sd(Latitude.scale β_2_) | 23.79* | 5.95 | 14.63 | 37.73 | 1.00 | 2456 | 4573 |
| cor(Intercept, Latitude.scale β_1_) | 0.52 | 0.31 | -0.26 | 0.92 | 1.00 | 1105 | 1692 |
| cor(Intercept, Latitude.scale β_2_) | 0.27 | 0.29 | -0.34 | 0.76 | 1.00 | 2185 | 3315 |
| cor(Latitude.scale β_1_, Latitude.scale β_2_) | 0.21 | 0.24 | -0.29 | 0.63 | 1.00 | 4292 | 5397 |
| Population-level effects |  |  |  |  |  |  |  |
| Intercept | -0.06* | 0.14 | -0.37 | 0.17 | 1.00 | 1026 | 1491 |
| Latitude.scale β_1_ | 10.73 | 5.39 | -0.08 | 21.20 | 1.00 | 6023 | 5000 |
| Latitude.scale β_2_ | 3.68 | 4.48 | -5.15 | 12.47 | 1.00 | 7838 | 5971 |
| Family specific parameters |  |  |  |  |  |  |  |
| Sigma | 0.33* | 0.01 | 0.32 | 0.35 | 1.00 | 14551 | 4897 |

*Note*: Model random effects correspond to either elevational or latitudinal zones; parameter uncertainties (est. error) are presented as standard deviations; L- and U-95% CI denote lower and upper 95% credible intervals; Bulk EES denotes bulk effective sample size produced using rank normalized draws; Tail EES denotes tail effective sample size produced by computing the minimum of effective sample sizes for 5% and 95% quantiles; β_1_ and β_2_ denote slope coefficients for first- and second-degree orthogonal polynomial predictors, respectively; and * denotes parameters with 95% credible intervals that do not include zero.**Table S7.** Results for generalized linear multilevel/mixed effect models evaluating relationships between waterbody area, depth, elevation, latitude, and species richness

| **Parameter** | **Estimate** | **Est.**  **Error** | **L-95%**  **Cl** | **U-95%**  **CI** | **Rhat** | **Bulk**  **ESS** | **Tail**  **ESS** |
| --- | --- | --- | --- | --- | --- | --- | --- |
| **(a) Species richness ~ Area** |  |  |  |  |  |  |  |
| Population-level effects |  |  |  |  |  |  |  |
| Intercept | 1.67* | 0.02 | 1.64 | 1.71 | 1.00 | 6196 | 5183 |
| Area.scale | 0.05* | 0.02 | 0.01 | 0.08 | 1.00 | 6465 | 4874 |
| Family specific parameters |  |  |  |  |  |  |  |
| Shape | 6.01* | 0.50 | 5.12 | 7.08 | 1.00 | 6110 | 5137 |
| **(b) Species richness ~ Area + Elevation Poly (Latitude Random Effect)** | | |  |  |  |  |  |
| Group-level effects |  |  |  |  |  |  |  |
| sd(Intercept) | 0.21* | 0.09 | 0.09 | 0.42 | 1.00 | 2431 | 4580 |
| sd(Elevation.scale β_1_) | 5.06* | 1.73 | 2.3 | 8.95 | 1.00 | 3228 | 4434 |
| sd(Elevation.scale β_2_) | 4.09* | 1.62 | 1.61 | 7.97 | 1.00 | 4189 | 4201 |
| cor(Intercept, Elevation.scale β_1_) | -0.06 | 0.33 | -0.67 | 0.57 | 1.00 | 5131 | 5325 |
| cor(Intercept, Elevation.scale β_2_) | 0.25 | 0.37 | -0.54 | 0.83 | 1.00 | 5631 | 5808 |
| cor(Elevation.scale β_1_, Elevation.scale β_2_ | 0.48 | 0.29 | -0.21 | 0.90 | 1.00 | 5107 | 5936 |
| Population-level effects |  |  |  |  |  |  |  |
| Intercept | 1.61* | 0.07 | 1.46 | 1.75 | 1.00 | 2782 | 4040 |
| Elevation.scale β_1_ | -8.99* | 2.43 | -13.4 | -3.92 | 1.00 | 2639 | 4250 |
| Elevation.scale β_2_ | -4.86* | 1.87 | -8.46 | -0.96 | 1.00 | 4102 | 4449 |
| Area.scale | 0.01 | 0.01 | -0.01 | 0.04 | 1.00 | 11412 | 6214 |
| Family specific parameters |  |  |  |  |  |  |  |
| Shape | 11.98* | 1.48 | 9.48 | 15.33 | 1.00 | 11169 | 5918 |
| **(c) Species richness ~ Area + Latitude Poly (Elevation Random Effect)** | | |  |  |  |  |  |
| Group-level effects |  |  |  |  |  |  |  |
| sd(Intercept) | 0.33* | 0.09 | 0.19 | 0.56 | 1.00 | 2342 | 3844 |
| sd(Latitude.scale β_1_) | 3.79* | 1.64 | 1.08 | 7.62 | 1.00 | 2426 | 3497 |
| sd(Latitude.scale β_2_) | 5.52* | 1.96 | 2.44 | 10.07 | 1.00 | 2575 | 4198 |
| cor(Intercept, Latitude.scale β_1_) | 0.07 | 0.4 | -0.68 | 0.79 | 1.00 | 5789 | 5581 |
| cor(Intercept, Latitude.scale β_2_) | -0.52 | 0.28 | -0.91 | 0.14 | 1.00 | 4094 | 5031 |
| cor(Latitude.scale β_1_, Latitude.scale β_2_ | -0.31 | 0.39 | -0.92 | 0.50 | 1.00 | 2594 | 4688 |
| Population -level effects |  |  |  |  |  |  |  |
| Intercept | 1.66* | 0.10 | 1.46 | 1.86 | 1.00 | 2086 | 3220 |
| Latitude.scale β_1_ | 0.58 | 1.65 | -2.84 | 3.66 | 1.00 | 4255 | 5110 |
| Latitude.scale β_2_ | -2.25 | 1.82 | -5.69 | 1.53 | 1.00 | 4168 | 5240 |
| Area.scale | 0.02 | 0.01 | -0.01 | 0.05 | 1.00 | 13277 | 5573 |
| Family specific parameters |  |  |  |  |  |  |  |
| Shape | 11.54* | 1.42 | 9.14 | 14.74 | 1.00 | 9839 | 5708 |
| **(d) Area ~ Elevation** |  |  |  |  |  |  |  |
| Population-level effects |  |  |  |  |  |  |  |
| Intercept | 0.55* | 0.02 | 0.51 | 0.59 | 1.00 | 3423 | 3547 |
| Elevation.scale | -0.03* | 0.01 | -0.05 | -0.01 | 1.00 | 4961 | 4568 |
| Family specific parameters |  |  |  |  |  |  |  |
| Sigma | 0.61* | 0.01 | 0.59 | 0.64 | 1.00 | 3997 | 4078 |
| Alpha | 22.85* | 2.29 | 18.58 | 27.58 | 1.00 | 4246 | 3702 |
| **(e) Area ~ Latitude** |  |  |  |  |  |  |  |
| Population-level effects |  |  |  |  |  |  |  |
| Intercept | 0.55* | 0.02 | 0.52 | 0.59 | 1.00 | 3455 | 3745 |
| Latitude.scale | 0.02 | 0.01 | 0.00 | 0.03 | 1.00 | 4645 | 4272 |
| Family specific parameters |  |  |  |  |  |  |  |
| Sigma | 0.61* | 0.01 | 0.59 | 0.64 | 1.00 | 3890 | 4279 |
| Alpha | 23.05* | 2.18 | 19.00 | 27.51 | 1.00 | 4146 | 4352 |
| **(f) Species richness ~ Depth** |  |  |  |  |  |  |  |
| Population-level effects |  |  |  |  |  |  |  |
| Intercept | 1.67* | 0.02 | 1.64 | 1.71 | 1.00 | 5931 | 5013 |
| Depth.scale | 0.08* | 0.02 | 0.05 | 0.11 | 1.00 | 6487 | 5081 |
| Family specific parameters |  |  |  |  |  |  |  |
| Shape | 6.18* | 0.53 | 5.22 | 7.30 | 1.00 | 5880 | 4701 |
| **(g) Species richness ~ Depth + Elevation Poly (Latitude Random Effect)** | | |  |  |  |  |  |
| Group-level effects |  |  |  |  |  |  |  |
| sd(Intercept) | 0.20* | 0.09 | 0.08 | 0.41 | 1.00 | 2163 | 3823 |
| sd(Elevation.scale β_1_) | 5.07* | 1.69 | 2.34 | 8.95 | 1.00 | 3392 | 4322 |
| sd(Elevation.scale β_2_) | 4.05* | 1.56 | 1.61 | 7.73 | 1.00 | 4201 | 4701 |
| cor(Intercept, Elevation.scale β_1_) | -0.05 | 0.33 | -0.67 | 0.58 | 1.00 | 4716 | 5256 |
| cor(Intercept, Elevation.scale β_2_) | 0.23 | 0.37 | -0.56 | 0.82 | 1.00 | 6215 | 5707 |
| cor(Elevation.scale β_1_, Elevation.scale β_2_ | 0.49 | 0.29 | -0.19 | 0.91 | 1.00 | 5003 | 6203 |
| Population -level effects |  |  |  |  |  |  |  |
| Intercept | 1.61* | 0.07 | 1.46 | 1.76 | 1.00 | 3128 | 3793 |
| Elevation.scale β_1_ | -8.82* | 2.38 | -13.05 | -3.77 | 1.00 | 2731 | 4967 |
| Elevation.scale β_2_ | -5.10* | 1.88 | -8.62 | -1.29 | 1.00 | 4591 | 5076 |
| Depth.scale | 0.02 | 0.02 | -0.01 | 0.05 | 1.00 | 13813 | 5354 |
| Family specific parameters |  |  |  |  |  |  |  |
| Shape | 12.04* | 1.49 | 9.51 | 15.27 | 1.00 | 11676 | 5738 |
| **(h) Species richness ~ Depth + Latitude Poly (Elevation Random Effect)** | | |  |  |  |  |  |
| Group-level effects |  |  |  |  |  |  |  |
| sd(Intercept) | 0.31* | 0.09 | 0.19 | 0.52 | 1.00 | 2660 | 4416 |
| sd(Latitude.scale β_1_) | 3.69* | 1.58 | 0.94 | 7.27 | 1.00 | 2595 | 2095 |
| sd(Latitude.scale β_2_) | 5.32* | 1.87 | 2.32 | 9.66 | 1.00 | 2834 | 4631 |
| cor(Intercept, Latitude.scale β_1_) | 0.11 | 0.39 | -0.65 | 0.82 | 1.00 | 4912 | 4199 |
| cor(Intercept, Latitude.scale β_2_) | -0.51 | 0.27 | -0.90 | 0.13 | 1.00 | 4749 | 5469 |
| cor(Latitude.scale β_1_, Latitude.scale β_2_ | -0.35 | 0.38 | -0.93 | 0.47 | 1.00 | 2823 | 4370 |
| Population -level effects |  |  |  |  |  |  |  |
| Intercept | 1.65* | 0.10 | 1.46 | 1.85 | 1.00 | 2075 | 3580 |
| Latitude.scale β_1_ | 0.45 | 1.62 | -2.81 | 3.55 | 1.00 | 4565 | 5491 |
| Latitude.scale β_2_ | -2.21 | 1.82 | -5.65 | 1.60 | 1.00 | 3894 | 5389 |
| Depth.scale | 0.04 | 0.02 | 0.00 | 0.07 | 1.00 | 11657 | 6051 |
| Family specific parameters |  |  |  |  |  |  |  |
| Shape | 11.60* | 1.44 | 9.13 | 14.79 | 1.00 | 10489 | 5526 |
| **(j) Depth ~ Elevation** |  |  |  |  |  |  |  |
| Population-level effects |  |  |  |  |  |  |  |
| Intercept | 0.32* | 0.02 | 0.29 | 0.36 | 1.00 | 3174 | 3273 |
| Elevation.scale | -0.05* | 0.01 | -0.08 | -0.03 | 1.00 | 4279 | 4670 |
| Family specific parameters |  |  |  |  |  |  |  |
| Sigma | 0.68* | 0.01 | 0.65 | 0.70 | 1.00 | 3330 | 3332 |
| Alpha | 24.18* | 2.25 | 19.99 | 28.79 | 1.00 | 3802 | 3634 |
| **(i) Depth ~ Latitude** |  |  |  |  |  |  |  |
| Population-level effects |  |  |  |  |  |  |  |
| Intercept | 0.34* | 0.02 | 0.30 | 0.37 | 1.00 | 3270 | 3665 |
| Latitude.scale | 0.03* | 0.01 | 0.01 | 0.04 | 1.00 | 4950 | 4763 |
| Family specific parameters |  |  |  |  |  |  |  |
| Sigma | 0.68* | 0.01 | 0.65 | 0.71 | 1.00 | 3231 | 3897 |
| Alpha | 24.49* | 2.29 | 20.3 | 29.12 | 1.00 | 4499 | 4315 |

*Note*: Generalized linear models with species richness responses were negative binomial with log link; generalized linear models with depth and area response were skew normal; random effects correspond to either elevational or latitudinal zones; parameter uncertainties (est. error) are presented as standard deviations; L- and U-95% CI denote lower and upper 95% credible intervals; Bulk EES denotes bulk effective sample size produced using rank normalized draws; Tail EES denotes tail effective sample size produced by computing the minimum of effective sample sizes for 5% and 95% quantiles; β_1_ and β_2_ denote slope coefficients for first- and second-degree orthogonal polynomial predictors (of elevation and latitude), respectively; and * denotes parameters with 95% credible intervals that do not include zero.**Table S8.** Results for generalized linear multilevel/mixed effect models evaluating relationships between sampling effort, sampling year, elevation, latitude, and species richness

| **Parameter** | **Estimate** | **Est.**  **Error** | **L-95%**  **Cl** | **U-95%**  **CI** | **Rhat** | **Bulk**  **ESS** | **Tail**  **ESS** |
| --- | --- | --- | --- | --- | --- | --- | --- |
| **(b) Species richness ~ Estimated number of sampling events** | | | | | | | |
| Population-level effects |  |  |  |  |  |  |  |
| Intercept | 1.65* | 0.02 | 1.62 | 1.68 | 1.00 | 5683 | 4794 |
| Sampling.events.scale | 0.19* | 0.02 | 0.16 | 0.23 | 1.00 | 5152 | 4306 |
| Family specific parameters |  |  |  |  |  |  |  |
| shape | 7.46* | 0.69 | 6.26 | 8.96 | 1.00 | 5461 | 4691 |
| **(c) Species richness ~ Estimated number of sampling events + Elevation Poly (Latitude Random Effect)** | | | | | | | |
| Group-level effects |  |  |  |  |  |  |  |
| sd(Intercept) | 0.19* | 0.08 | 0.08 | 0.39 | 1.00 | 1776 | 4007 |
| sd(Elevation.scale β_1_) | 4.86* | 1.60 | 2.30 | 8.58 | 1.00 | 3266 | 4642 |
| sd(Elevation.scale β_2_) | 2.71* | 1.41 | 0.50 | 6.04 | 1.00 | 3310 | 3164 |
| cor(Intercept, Elevation.scale β_1_) | 0.07 | 0.33 | -0.58 | 0.67 | 1.00 | 4575 | 5642 |
| cor(Intercept, Elevation.scale β_2_) | 0.13 | 0.43 | -0.73 | 0.83 | 1.00 | 6135 | 5483 |
| cor(Elevation.scale β_1_, Elevation.scale β_2_ | 0.55 | 0.32 | -0.23 | 0.96 | 1.00 | 5765 | 6114 |
| Population -level effects |  |  |  |  |  |  |  |
| Intercept | 1.61* | 0.07 | 1.47 | 1.75 | 1.00 | 2748 | 3455 |
| Elevation.scale β_1_ | -8.09* | 2.16 | -12.06 | -3.72 | 1.00 | 2488 | 4209 |
| Elevation.scale β_2_ | -6.01* | 1.50 | -8.79 | -2.88 | 1.00 | 4095 | 5110 |
| Sampling.events.scale | 0.14* | 0.02 | 0.11 | 0.17 | 1.00 | 9414 | 6348 |
| Family specific parameters |  |  |  |  |  |  |  |
| shape | 13.93* | 1.87 | 10.84 | 18.17 | 1.00 | 10043 | 6170 |
| **(d) Species richness ~ Estimated number of sampling events + Latitude poly (Elevation random effect)** | | | | | | | |
| Group-level effects |  |  |  |  |  |  |  |
| sd(Intercept) | 0.29* | 0.08 | 0.17 | 0.48 | 1.00 | 2522 | 3736 |
| sd(Latitude.scale β_1_) | 2.84* | 1.57 | 0.24 | 6.43 | 1.00 | 2559 | 2455 |
| sd(Latitude.scale β_2_) | 5.40* | 1.83 | 2.44 | 9.58 | 1.00 | 2843 | 5187 |
| cor(Intercept, Latitude.scale β_1_) | 0.12 | 0.43 | -0.72 | 0.85 | 1.00 | 6392 | 5071 |
| cor(Intercept, Latitude.scale β_2_) | -0.49 | 0.27 | -0.89 | 0.14 | 1.00 | 4037 | 5415 |
| cor(Latitude.scale β_1_, Latitude.scale β_2_ | -0.27 | 0.42 | -0.91 | 0.60 | 1.00 | 2046 | 3244 |
| Population -level effects |  |  |  |  |  |  |  |
| Intercept | 1.63* | 0.09 | 1.45 | 1.81 | 1.00 | 1965 | 3064 |
| Latitude.scale β_1_ | 0.93 | 1.46 | -2.09 | 3.65 | 1.00 | 4546 | 4897 |
| Latitude.scale β_2_ | -1.17 | 1.78 | -4.51 | 2.58 | 1.00 | 4453 | 5507 |
| Sampling.events.scale | 0.15* | 0.02 | 0.11 | 0.18 | 1.00 | 10599 | 6307 |
| Family specific parameters |  |  |  |  |  |  |  |
| shape | 13.54* | 1.79 | 10.54 | 17.55 | 1.00 | 10598 | 6196 |
| **(f) Species richness ~ Estimated number of sampling years** | | | | | | | |
| Population-level effects |  |  |  |  |  |  |  |
| Intercept | 1.65* | 0.02 | 1.62 | 1.68 | 1.00 | 6344 | 4821 |
| Sampling.years.scale | 0.21* | 0.01 | 0.18 | 0.23 | 1.00 | 6467 | 4951 |
| Family specific parameters |  |  |  |  |  |  |  |
| shape | 8.46* | 0.85 | 6.96 | 10.3 | 1.00 | 6187 | 4602 |
| **(g) Species richness ~ Estimated number of sampling years + Elevation Poly (Latitude Random Effect)** | | | | | | | |
| Group-level effects |  |  |  |  |  |  |  |
| sd(Intercept) | 0.23* | 0.09 | 0.08 | 0.44 | 1.00 | 1734 | 3446 |
| sd(Elevation.scale β_1_) | 3.89* | 1.93 | 0.41 | 7.96 | 1.00 | 1712 | 2388 |
| sd(Elevation.scale β_2_) | 3.33* | 1.52 | 0.81 | 6.72 | 1.00 | 2737 | 1970 |
| cor(Intercept, Elevation.scale β_1_) | 0.24 | 0.36 | -0.56 | 0.81 | 1.00 | 6319 | 5077 |
| cor(Intercept, Elevation.scale β_2_) | 0.09 | 0.41 | -0.73 | 0.77 | 1.00 | 5281 | 4523 |
| cor(Elevation.scale β_1_, Elevation.scale β_2_ | 0.20 | 0.38 | -0.61 | 0.84 | 1.00 | 4477 | 5331 |
| Population -level effects |  |  |  |  |  |  |  |
| Intercept | 1.63* | 0.08 | 1.47 | 1.79 | 1.00 | 3064 | 3952 |
| Elevation.scale β_1_ | -9.42* | 2.40 | -13.38 | -4.44 | 1.00 | 1741 | 4914 |
| Elevation.scale β_2_ | -4.52* | 1.58 | -7.70 | -1.42 | 1.00 | 5754 | 5491 |
| Sampling.years.scale | 0.21* | 0.01 | 0.18 | 0.23 | 1.00 | 10561 | 5638 |
| Family specific parameters |  |  |  |  |  |  |  |
| shape | 27.42* | 6.83 | 17.9 | 43.87 | 1.00 | 10258 | 4974 |
| **(h) Species richness ~ Estimated number of sampling years + Latitude poly (Elevation random effect)** | | | | | | | |
| Group-level effects |  |  |  |  |  |  |  |
| sd(Intercept) | 0.34* | 0.09 | 0.21 | 0.56 | 1.00 | 2503 | 3749 |
| sd(Latitude.scale β_1_) | 2.14* | 1.42 | 0.11 | 5.37 | 1.00 | 2531 | 3515 |
| sd(Latitude.scale β_2_) | 6.50* | 1.98 | 3.24 | 11.04 | 1.00 | 2701 | 4437 |
| cor(Intercept, Latitude.scale β_1_) | -0.05 | 0.46 | -0.85 | 0.82 | 1.00 | 6829 | 5399 |
| cor(Intercept, Latitude.scale β_2_) | -0.52 | 0.24 | -0.87 | 0.04 | 1.00 | 4120 | 5267 |
| cor(Latitude.scale β_1_, Latitude.scale β_2_ | -0.08 | 0.45 | -0.85 | 0.78 | 1.01 | 1488 | 2792 |
| Population -level effects |  |  |  |  |  |  |  |
| Intercept | 1.65* | 0.10 | 1.45 | 1.86 | 1.00 | 1318 | 3068 |
| Latitude.scale β_1_ | 0.16 | 1.31 | -2.50 | 2.62 | 1.00 | 4865 | 4960 |
| Latitude.scale β_2_ | -0.20 | 1.99 | -4.20 | 3.79 | 1.00 | 3115 | 4380 |
| Sampling.years.scale | 0.21* | 0.01 | 0.18 | 0.23 | 1.00 | 13624 | 5673 |
| Family specific parameters |  |  |  |  |  |  |  |
| shape | 27.36* | 6.72 | 18.00 | 43.56 | 1.00 | 12116 | 4719 |

*Note*: Generalized linear models were negative binomial family and log link; random effects correspond to either elevational or latitudinal zones; parameter uncertainties (est. error) are presented as standard deviations; L- and U-95% CI denote lower and upper 95% credible intervals; Bulk EES denotes bulk effective sample size produced using rank normalized draws; Tail EES denotes tail effective sample size produced by computing the minimum of effective sample sizes for 5% and 95% quantiles; β_1_ and β_2_ denote slope coefficients for first- and second-degree orthogonal polynomial predictors (of elevation and latitude), respectively; and * denotes parameters with 95% credible intervals that do not include zero.


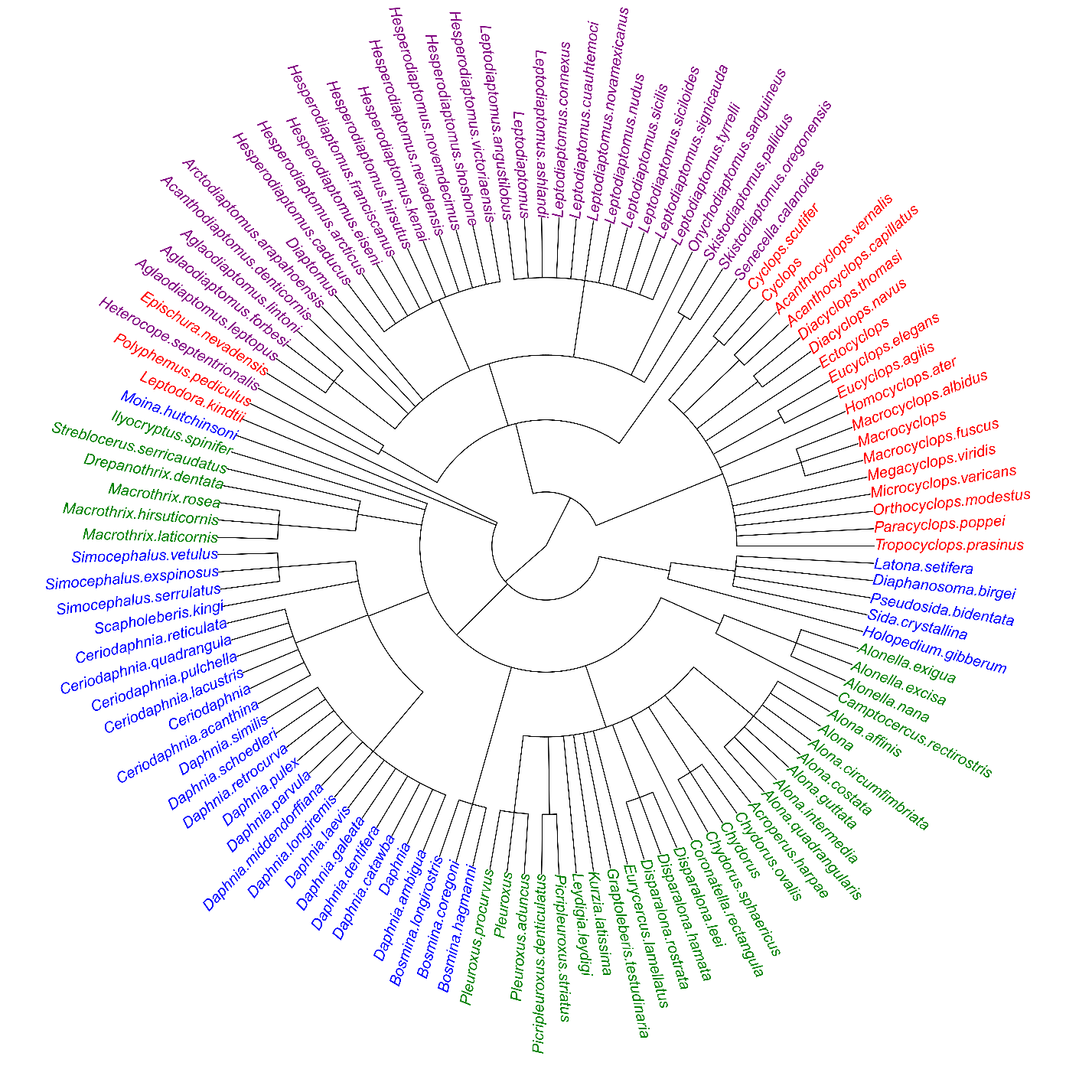


**Figure S1.** Fan plot of taxonomic rankings and feeding traits (substrate-grazing taxa = green; seston-filtering taxa = blue; stationary suspension-feeding taxa = purple; and raptorial-feeding taxa = red).


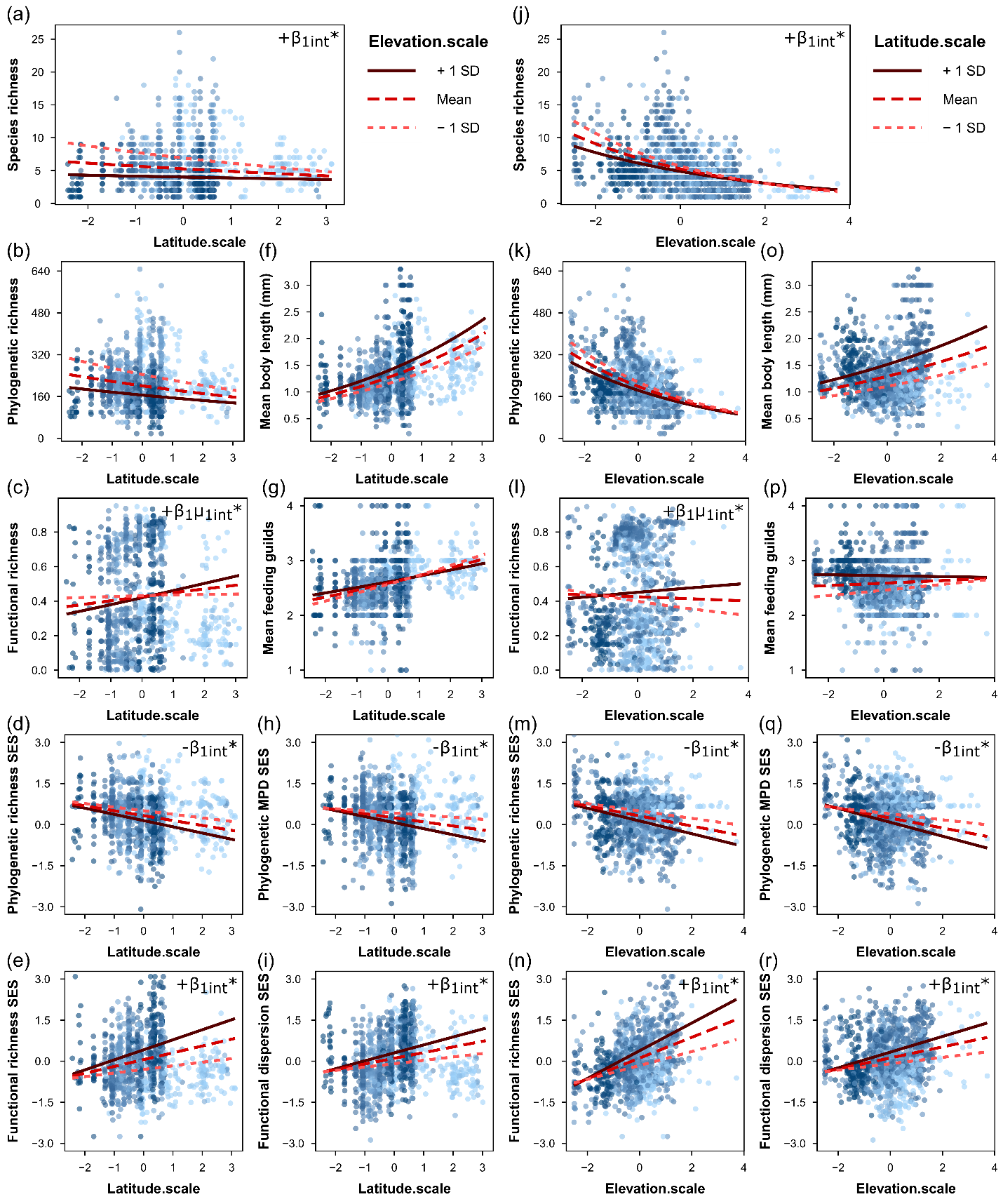


**Figure S2.** Predictions estimated from posterior draws of the linear predictor for generalized linear interaction models of each biodiversity metric regressed on latitude, elevation, and their multiplicative interaction. Panels (a–i) present latitudinal predictions at the mean elevation and +/- one standard deviation. Panels (j–r) present elevational predictions at the mean latitude and +/- one standard deviation. Elevation and latitude were centered and scaled (as *z*-scores). Mean population-level slope coefficients for multiplicative interaction terms with 95% probability of being either positive or negative are noted in the top right corners of each plot (β_1int_). Parameters for functional richness are from mixture models and assessed for each component separately (μ_1_ and μ_2_). Family and link distributions for response metrics as per Table S2. See Table S3 for detailed results.
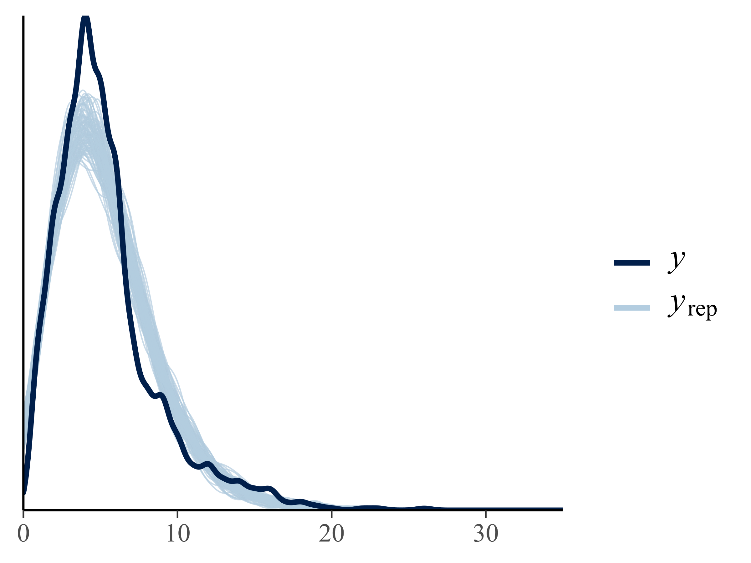


**Figure S3.** Posterior predictive check for generalized linear multilevel/mixed effect model of species richness regressed on first- and second-degree orthogonal polynomials of elevation (with latitudinal zone random effects) showing kernel density estimates for each of 100 draws from the posterior distribution (*y*_rep_) overlain with that of the response variable (*y*).

**
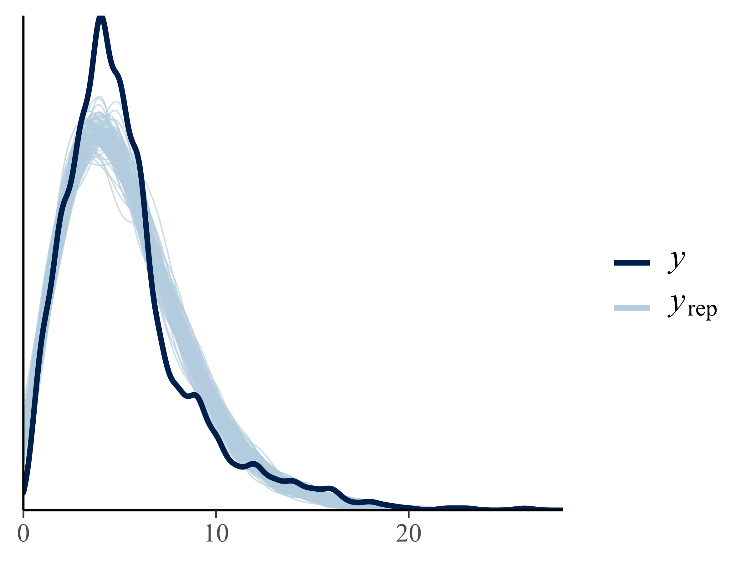
**

**Figure S4.** Posterior predictive check for generalized linear multilevel/mixed effect model of species richness regressed on first- and second-degree orthogonal polynomials of latitude (with elevational zone random effects) showing kernel density estimates for each of 100 draws from the posterior distribution (*y*_rep_) overlain with that of the response variable (*y*).

**
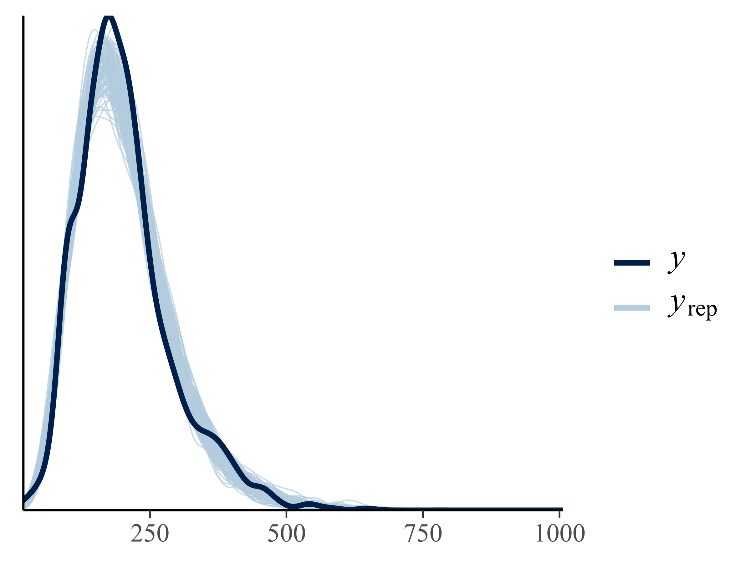
**

**Figure S5.** Posterior predictive check for generalized linear multilevel/mixed effect model of phylogenetic richness regressed on first- and second-degree orthogonal polynomials of elevation (with latitudinal zone random effects) showing kernel density estimates for each of 100 draws from the posterior distribution (*y*_rep_) overlain with that of the response variable (*y*).

**
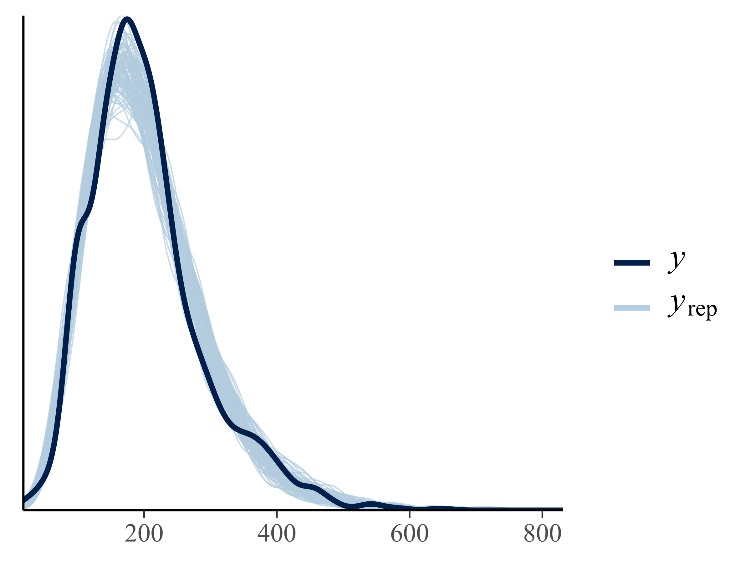
**

**Figure S6.** Posterior predictive check for generalized linear multilevel/mixed effect model of phylogenetic richness regressed on first- and second-degree orthogonal polynomials of latitude (with elevational zone random effects) showing kernel density estimates for each of 100 draws from the posterior distribution (*y*_rep_) overlain with that of the response variable (*y*).

**
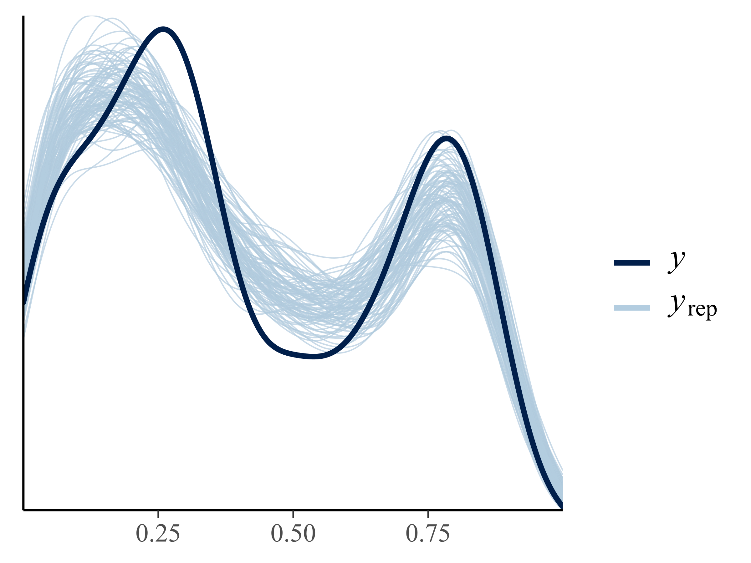
**

**Figure S7.** Posterior predictive check for generalized linear multilevel/mixed effect model of functional richness regressed on first- and second-degree orthogonal polynomials of elevation (with latitudinal zone random effects) showing kernel density estimates for each of 100 draws from the posterior distribution (*y*_rep_) overlain with that of the response variable (*y*).

**
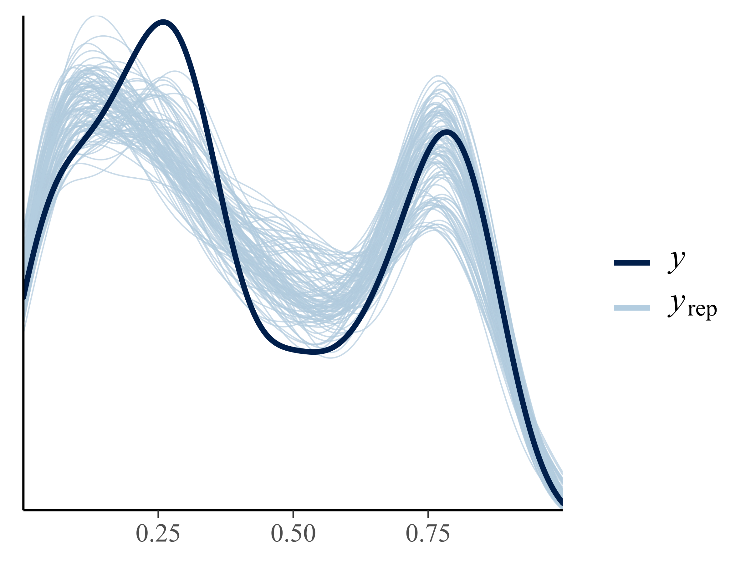
**

**Figure S8.** Posterior predictive check for generalized linear multilevel/mixed effect model of functional richness regressed on first- and second-degree orthogonal polynomials of latitude (with elevational zone random effects) showing kernel density estimates for each of 100 draws from the posterior distribution (*y*_rep_) overlain with that of the response variable (*y*).


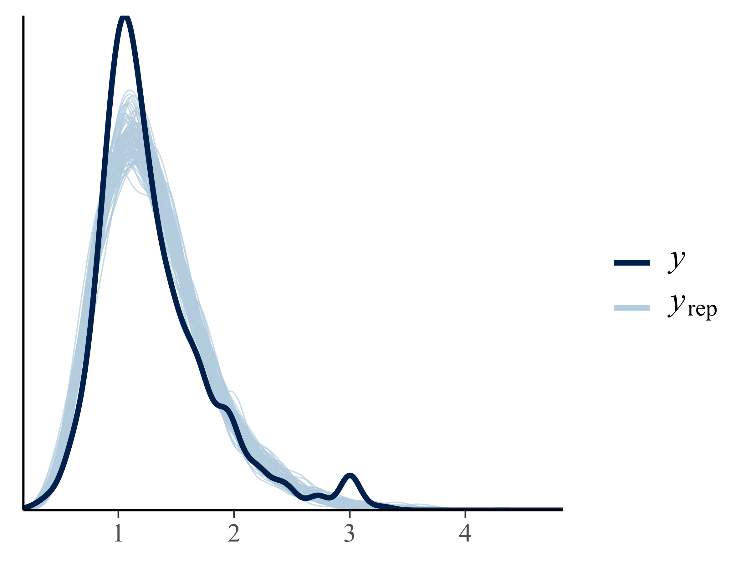


**Figure S9.** Posterior predictive check for generalized linear multilevel/mixed effect model of community mean body length regressed on first- and second-degree orthogonal polynomials of elevation (with latitudinal zone random effects) showing kernel density estimates for each of 100 draws from the posterior distribution (*y*_rep_) overlain with that of the response variable (*y*).


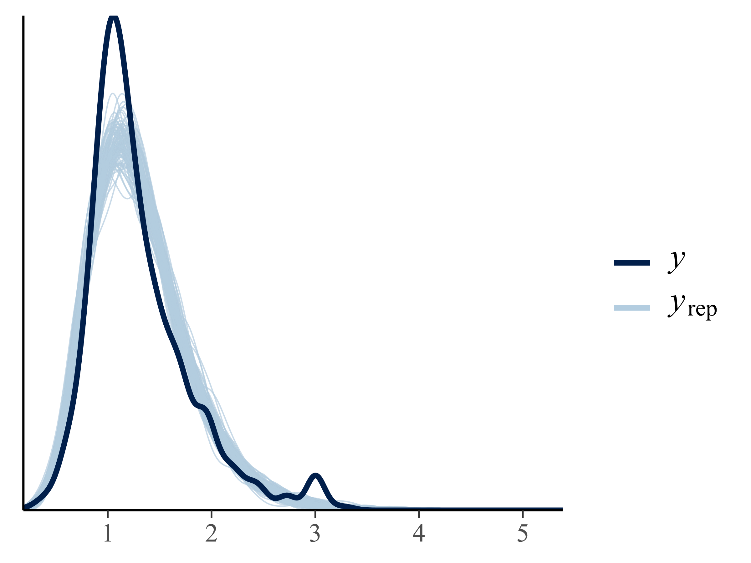


**Figure S10.** Posterior predictive check for generalized linear multilevel/mixed effect model of community mean body length regressed on first- and second-degree orthogonal polynomials of latitude (with elevational zone random effects) showing kernel density estimates for each of 100 draws from the posterior distribution (*y*_rep_) overlain with that of the response variable (*y*).


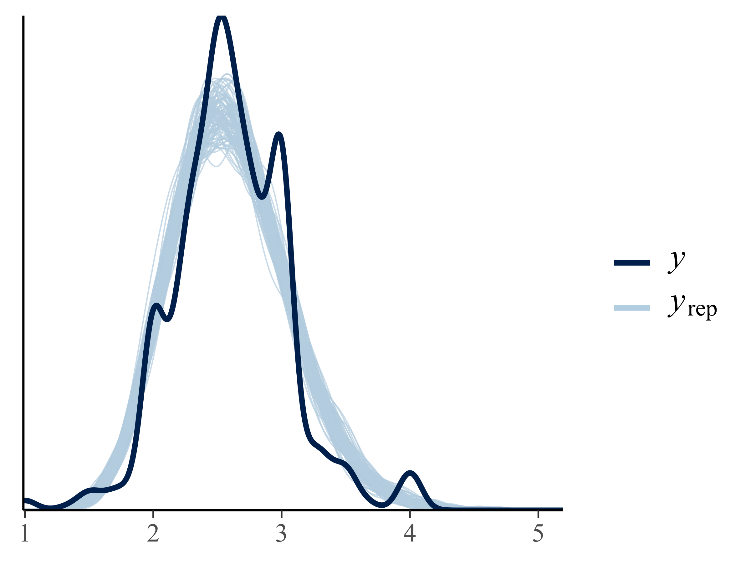


**Figure S11.** Posterior predictive check for generalized linear multilevel/mixed effect model of community mean feeding guilds regressed on first- and second-degree orthogonal polynomials of elevation (with latitudinal zone random effects) showing kernel density estimates for each of 100 draws from the posterior distribution (*y*_rep_) overlain with that of the response variable (*y*).


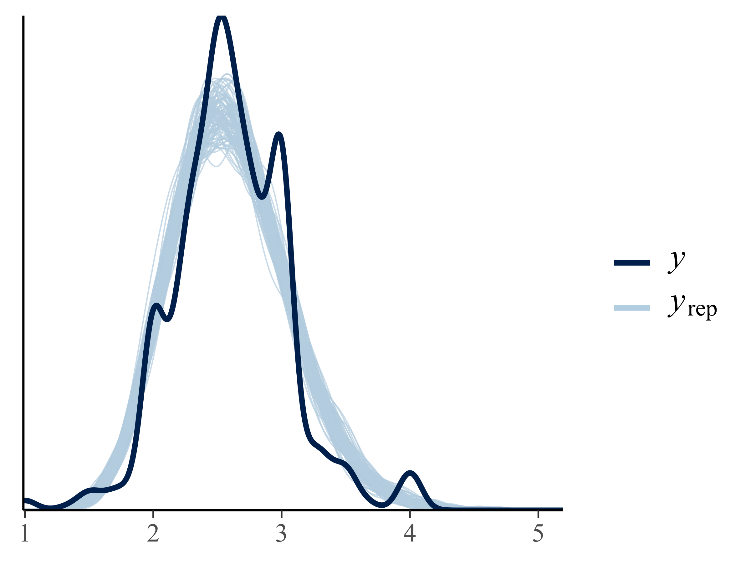


**Figure S12.** Posterior predictive check for generalized linear multilevel/mixed effect model of community mean feeding guilds regressed on first- and second-degree orthogonal polynomials of latitude (with elevational zone random effects) showing kernel density estimates for each of 100 draws from the posterior distribution (*y*_rep_) overlain with that of the response variable (*y*).

**
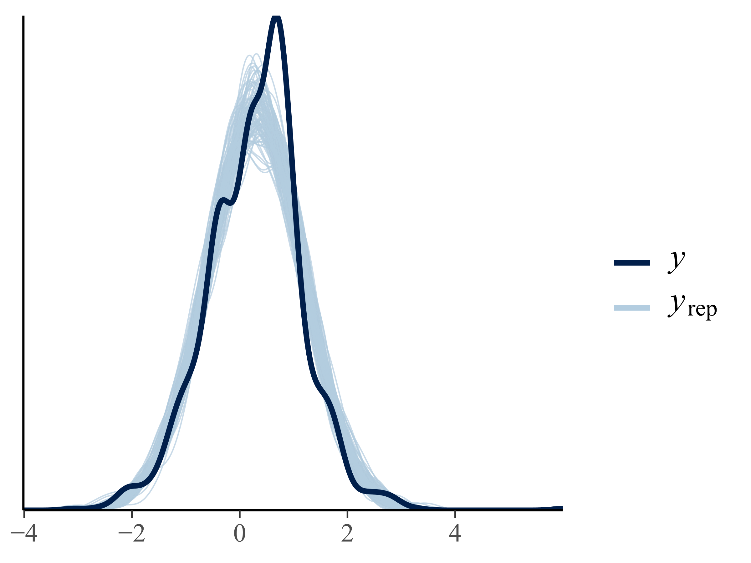
**

**Figure S13.** Posterior predictive check for generalized linear multilevel/mixed effect model of phylogenetic richness SES regressed on first- and second-degree orthogonal polynomials of elevation (with latitudinal zone random effects) showing kernel density estimates for each of 100 draws from the posterior distribution (*y*_rep_) overlain with that of the response variable (*y*).


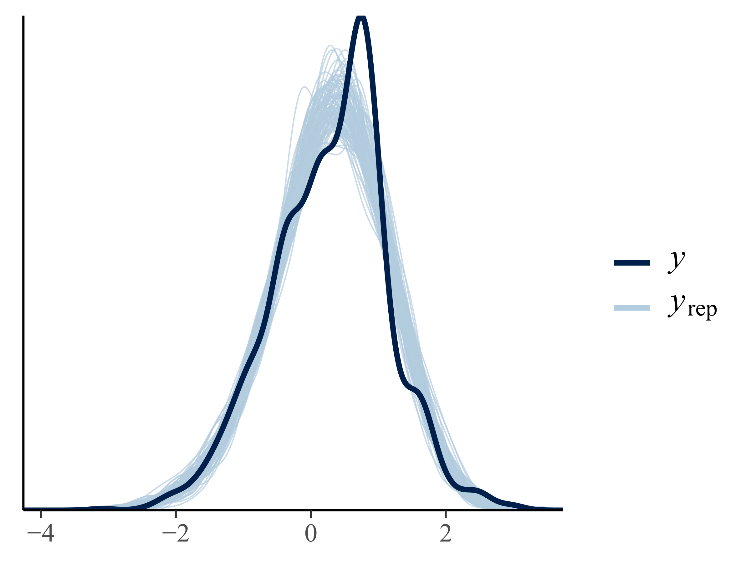


**Figure S14.** Posterior predictive check for generalized linear multilevel/mixed effect model of phylogenetic richness SES regressed on first- and second-degree orthogonal polynomials of latitude (with elevational zone random effects) showing kernel density estimates for each of 100 draws from the posterior distribution (*y*_rep_) overlain with that of the response variable (*y*).

**
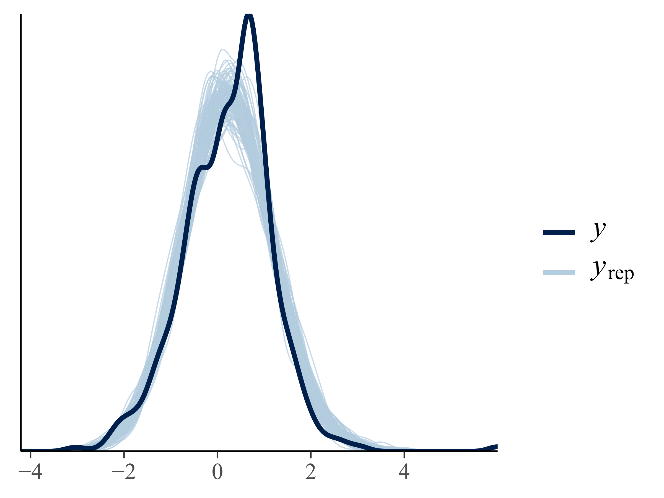
**

**Figure S15.** Posterior predictive check for generalized linear multilevel/mixed effect model of phylogenetic mean pairwise SES distance regressed on first- and second-degree orthogonal polynomials of elevation (with latitudinal zone random effects) showing kernel density estimates for each of 100 draws from the posterior distribution (*y*_rep_) overlain with that of the response variable (*y*).

**
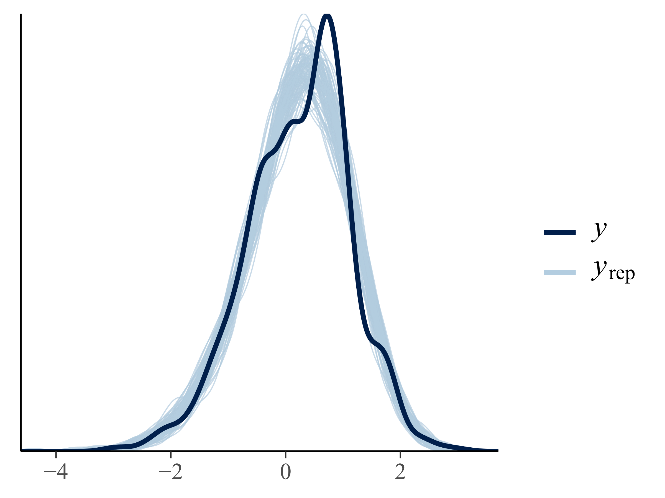
**

**Figure S16.** Posterior predictive check for generalized linear multilevel/mixed effect model of phylogenetic mean pairwise SES distance regressed on first- and second-degree orthogonal polynomials of latitude (with elevational zone random effects) showing kernel density estimates for each of 100 draws from the posterior distribution (*y*_rep_) overlain with that of the response variable (*y*).

**
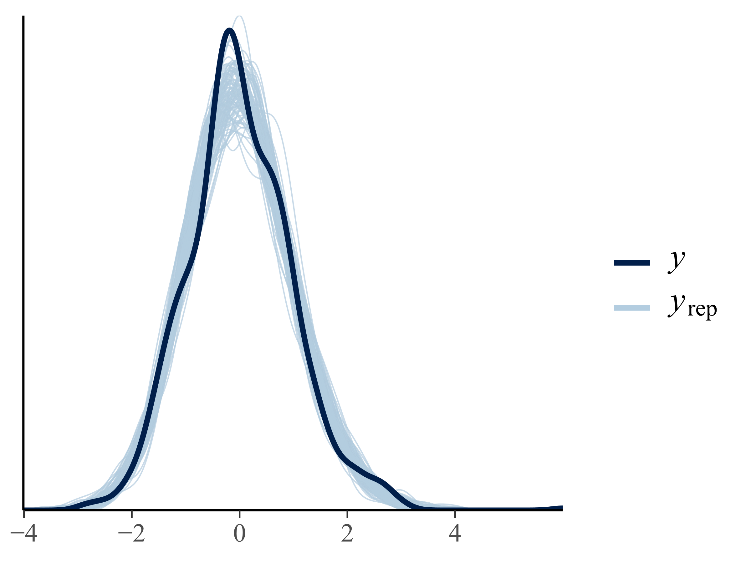
**

**Figure S17.** Posterior predictive check for generalized linear multilevel/mixed effect model of functional richness SES regressed on first- and second-degree orthogonal polynomials of elevation (with latitudinal zone random effects) showing kernel density estimates for each of 100 draws from the posterior distribution (*y*_rep_) overlain with that of the response variable (*y*).


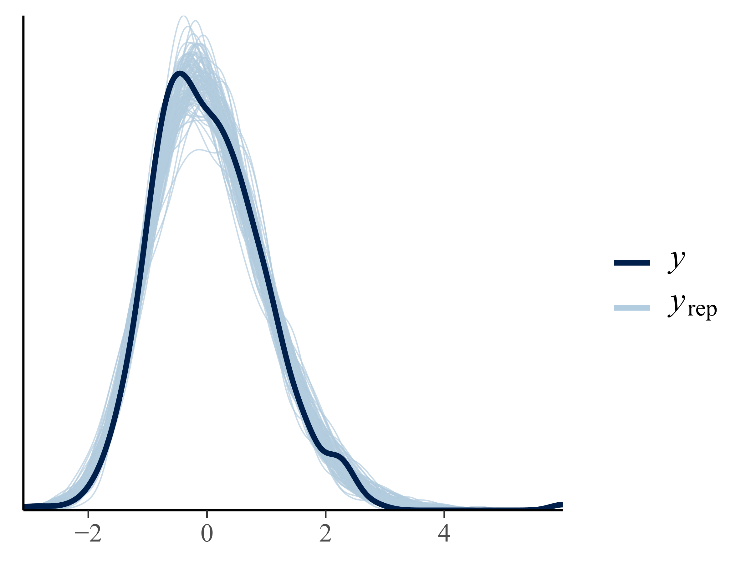


**Figure S18.** Posterior predictive check for generalized linear multilevel/mixed effect model of functional richness SES regressed on first- and second-degree orthogonal polynomials of latitude (with elevational zone random effects) showing kernel density estimates for each of 100 draws from the posterior distribution (*y*_rep_) overlain with that of the response variable (*y*).


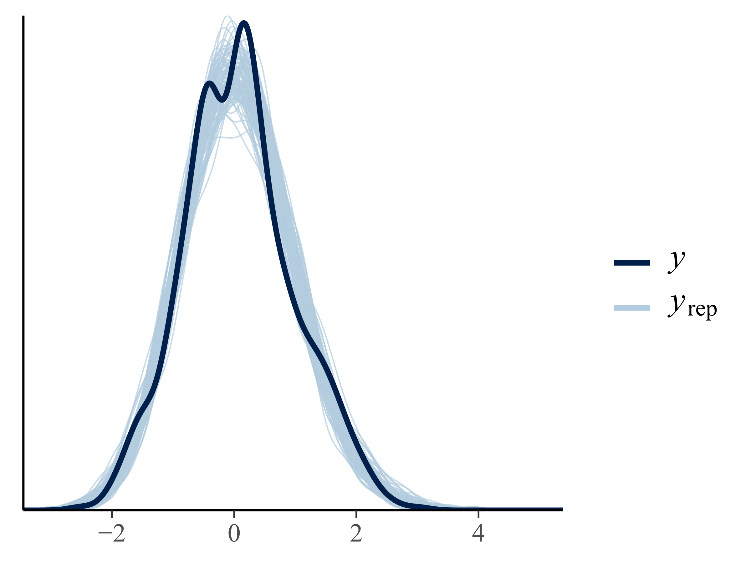


**Figure S19.** Posterior predictive check for generalized linear multilevel/mixed effect model of functional dispersion SES regressed on first- and second-degree orthogonal polynomials of elevation (with latitudinal zone random effects) showing kernel density estimates for each of 100 draws from the posterior distribution (*y*_rep_) overlain with that of the response variable (*y*).

**
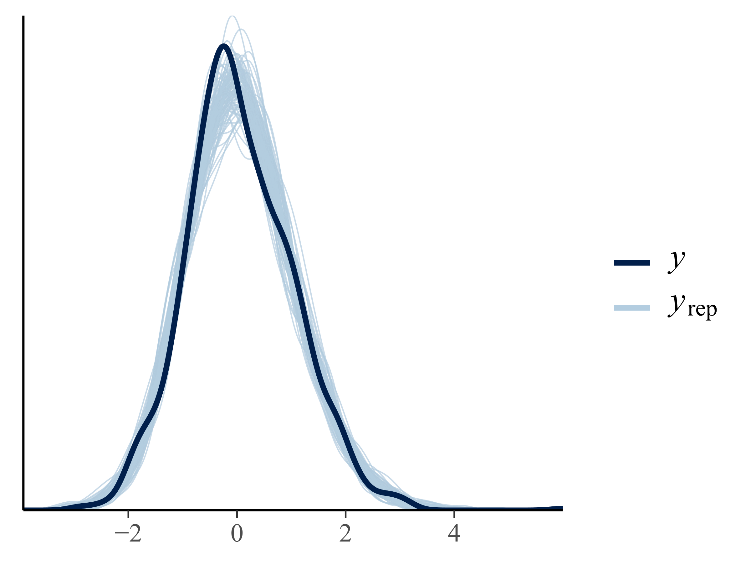
**

**Figure S20.** Posterior predictive check for generalized linear multilevel/mixed effect model of functional dispersion SES regressed on first- and second-degree orthogonal polynomials of latitude (with elevational zone random effects) showing kernel density estimates for each of 100 draws from the posterior distribution (*y*_rep_) overlain with that of the response variable (*y*).

**
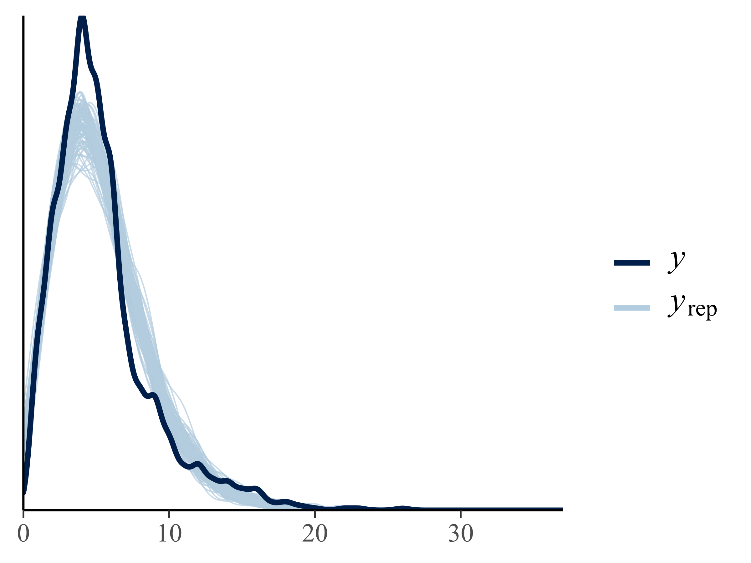
**

**Figure S21.** Posterior predictive check for generalized linear multilevel/mixed effect model of species richness regressed on first- and second-degree orthogonal polynomials of mean annual temperature (with latitudinal zone random effects) showing kernel density estimates for each of 100 draws from the posterior distribution (*y*_rep_) overlain with that of the response variable (*y*).


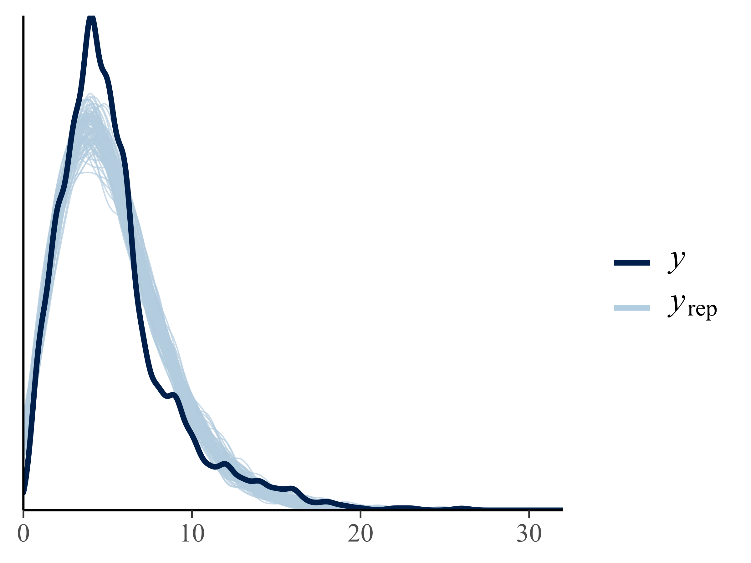


**Figure S22.** Posterior predictive check for generalized linear multilevel/mixed effect model of species richness regressed on first- and second-degree orthogonal polynomials of mean annual temperature (with elevational zone random effects) showing kernel density estimates for each of 100 draws from the posterior distribution (*y*_rep_) overlain with that of the response variable (*y*).


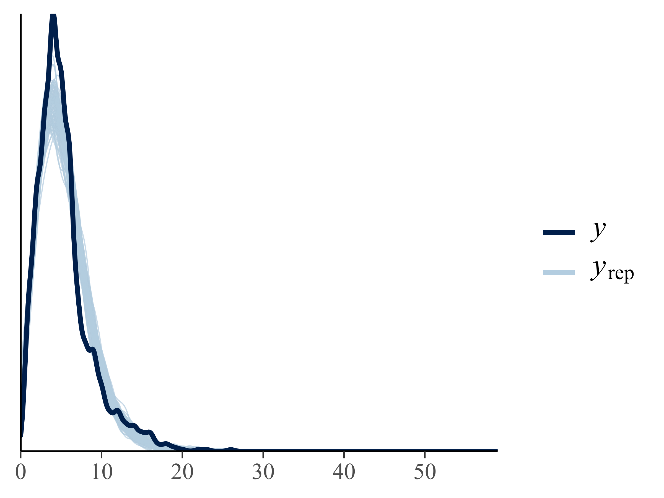


**Figure S23.** Posterior predictive check for generalized linear multilevel/mixed effect model of species richness regressed on first- and second-degree orthogonal polynomials of mean temperature difference (with latitudinal zone random effects) showing kernel density estimates for each of 100 draws from the posterior distribution (*y*_rep_) overlain with that of the response variable (*y*).


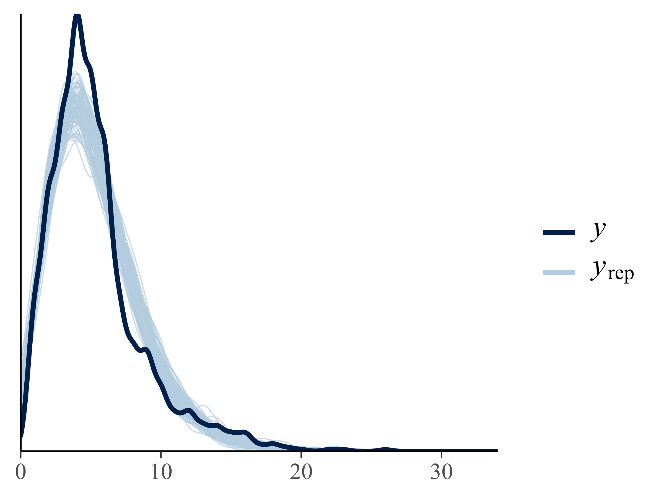


**Figure S24.** Posterior predictive check for generalized linear multilevel/mixed effect model of species richness regressed on first- and second-degree orthogonal polynomials of mean temperature difference (with elevational zone random effects) showing kernel density estimates for each of 100 draws from the posterior distribution (*y*_rep_) overlain with that of the response variable (*y*).


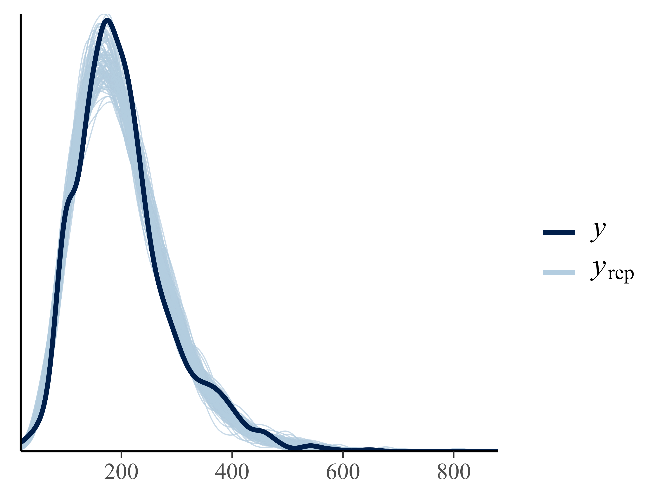


**Figure S25.** Posterior predictive check for generalized linear multilevel/mixed effect model of phylogenetic richness regressed on first- and second-degree orthogonal polynomials of mean annual temperature (with latitudinal zone random effects) showing kernel density estimates for each of 100 draws from the posterior distribution (*y*_rep_) overlain with that of the response variable (*y*).


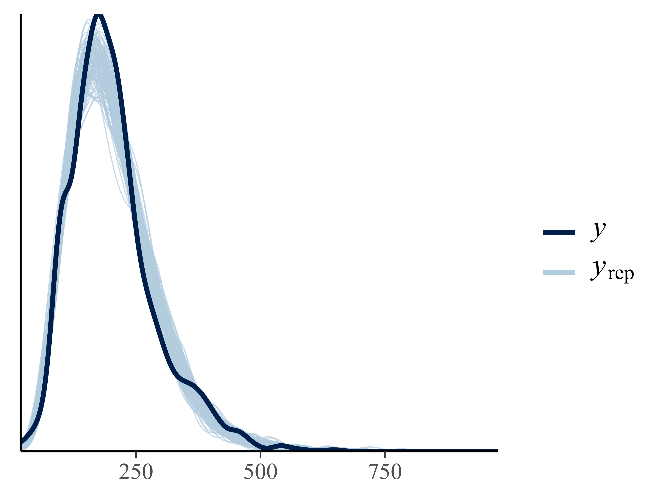


**Figure S26.** Posterior predictive check for generalized linear multilevel/mixed effect model of phylogenetic richness regressed on first- and second-degree orthogonal polynomials of mean annual temperature (with elevational zone random effects) showing kernel density estimates for each of 100 draws from the posterior distribution (*y*_rep_) overlain with that of the response variable (*y*).


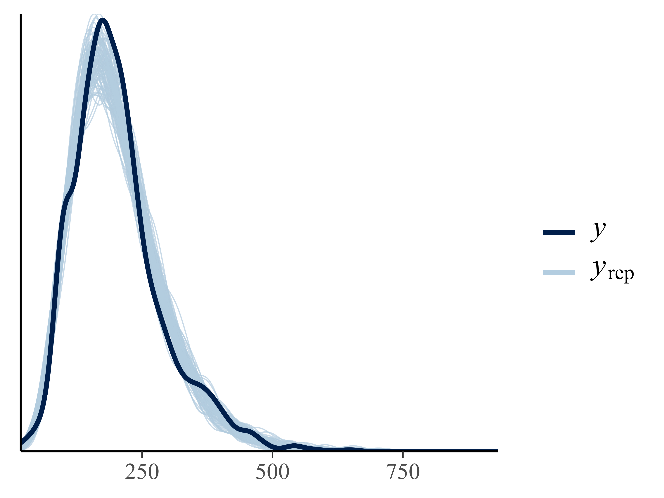


**Figure S27.** Posterior predictive check for generalized linear multilevel/mixed effect model of phylogenetic richness regressed on first- and second-degree orthogonal polynomials of mean temperature difference (with latitudinal zone random effects) showing kernel density estimates for each of 100 draws from the posterior distribution (*y*_rep_) overlain with that of the response variable (*y*).


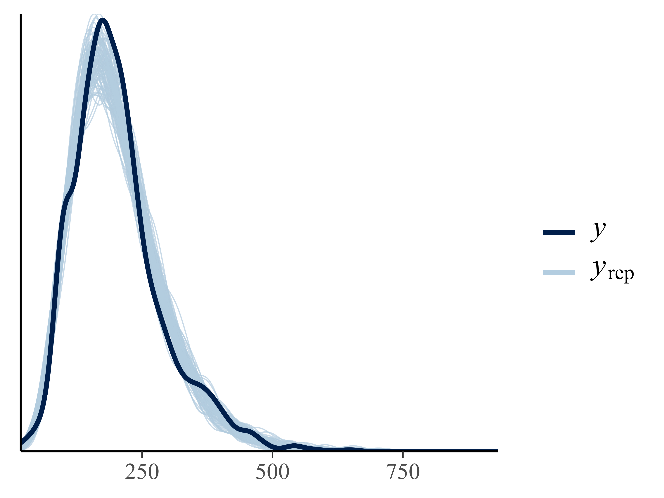


**Figure S28.** Posterior predictive check for generalized linear multilevel/mixed effect model of phylogenetic richness regressed on first- and second-degree orthogonal polynomials of mean temperature difference (with elevational zone random effects) showing kernel density estimates for each of 100 draws from the posterior distribution (*y*_rep_) overlain with that of the response variable (*y*).


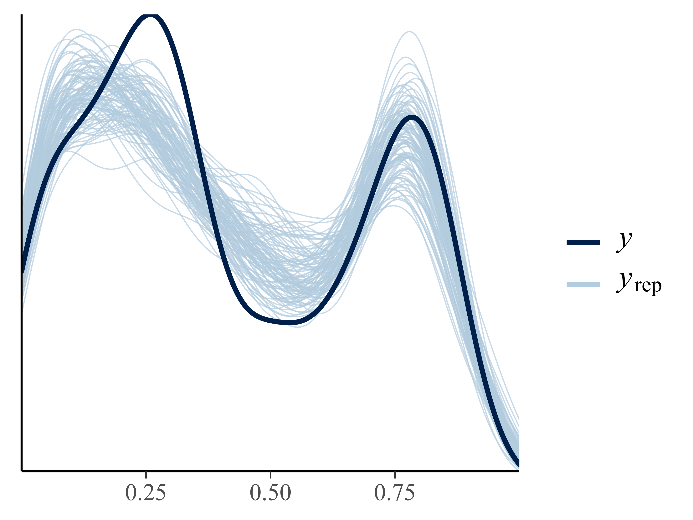


**Figure S29.** Posterior predictive check for generalized linear multilevel/mixed effect model of functional richness regressed on first- and second-degree orthogonal polynomials of mean annual temperature (with latitudinal zone random effects) showing kernel density estimates for each of 100 draws from the posterior distribution (*y*_rep_) overlain with that of the response variable (*y*).


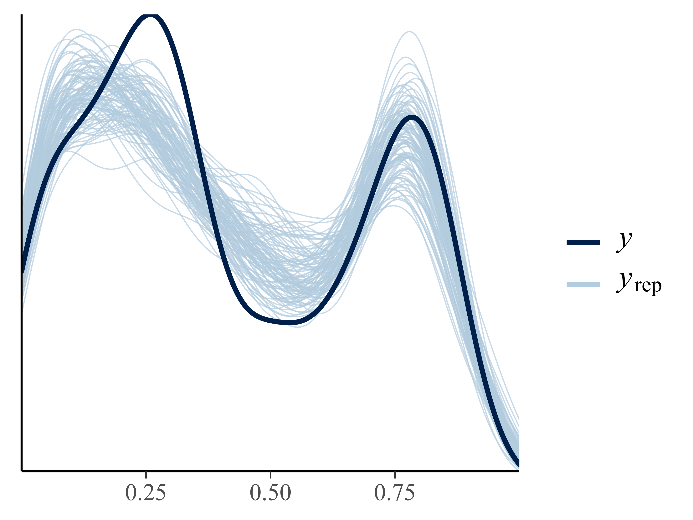


**Figure S30.** Posterior predictive check for generalized linear multilevel/mixed effect model of functional richness regressed on first- and second-degree orthogonal polynomials of mean annual temperature (with elevational zone random effects) showing kernel density estimates for each of 100 draws from the posterior distribution (*y*_rep_) overlain with that of the response variable (*y*).


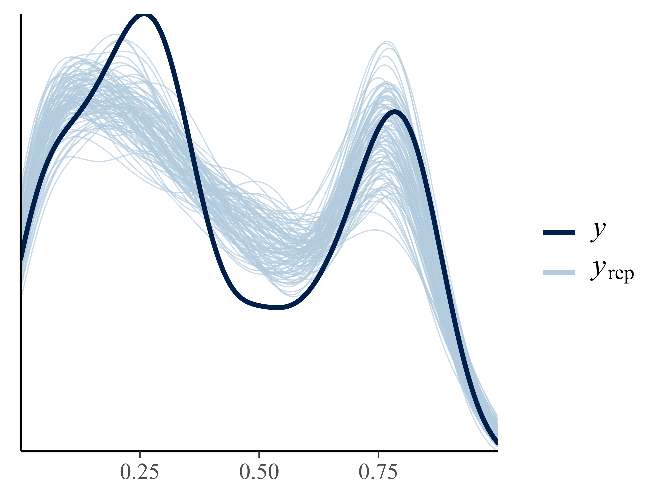


**Figure S31.** Posterior predictive check for generalized linear multilevel/mixed effect model of functional richness regressed on first- and second-degree orthogonal polynomials of mean temperature difference (with latitudinal zone random effects) showing kernel density estimates for each of 100 draws from the posterior distribution (*y*_rep_) overlain with that of the response variable (*y*).


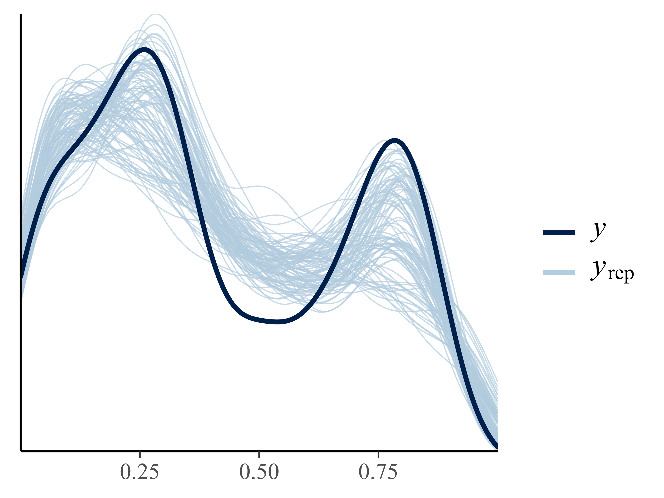


**Figure S32.** Posterior predictive check for generalized linear multilevel/mixed effect model of functional richness regressed on first- and second-degree orthogonal polynomials of mean temperature difference (with elevational zone random effects) showing kernel density estimates for each of 100 draws from the posterior distribution (*y*_rep_) overlain with that of the response variable (*y*).

**
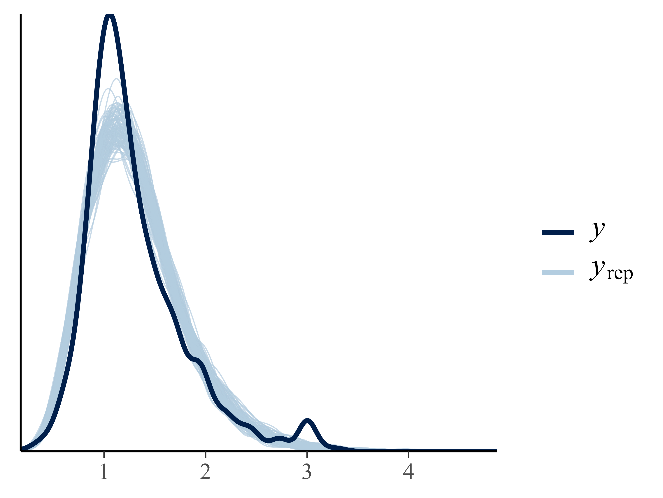
**

**Figure S33.** Posterior predictive check for generalized linear multilevel/mixed effect model of community mean body length regressed on first- and second-degree orthogonal polynomials of mean annual temperature (with latitudinal zone random effects) showing kernel density estimates for each of 100 draws from the posterior distribution (*y*_rep_) overlain with that of the response variable (*y*).


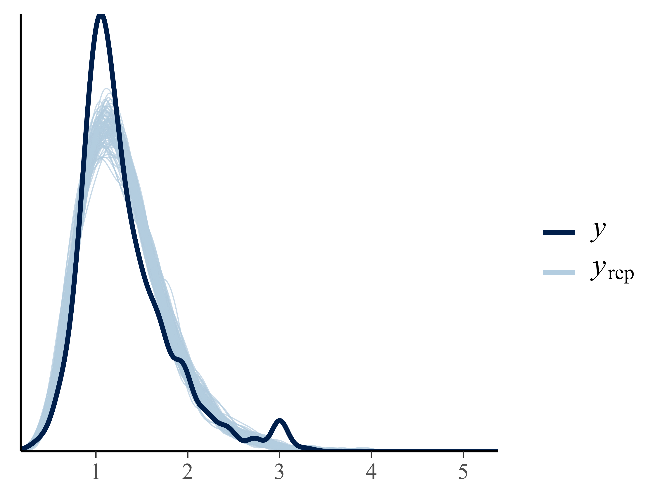


**Figure S34.** Posterior predictive check for generalized linear multilevel/mixed effect model of community mean body length regressed on first- and second-degree orthogonal polynomials of mean annual temperature (with elevational zone random effects) showing kernel density estimates for each of 100 draws from the posterior distribution (*y*_rep_) overlain with that of the response variable (*y*).

**
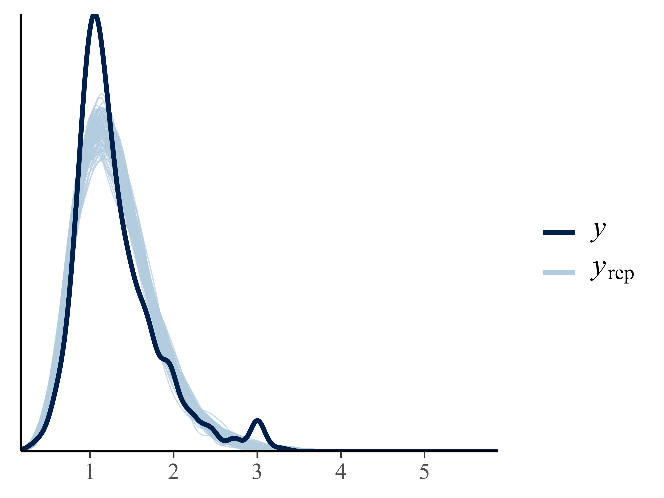
**

**Figure S35.** Posterior predictive check for generalized linear multilevel/mixed effect model of community mean body length regressed on first- and second-degree orthogonal polynomials of mean temperature difference (with latitudinal zone random effects) showing kernel density estimates for each of 100 draws from the posterior distribution (*y*_rep_) overlain with that of the response variable (*y*).


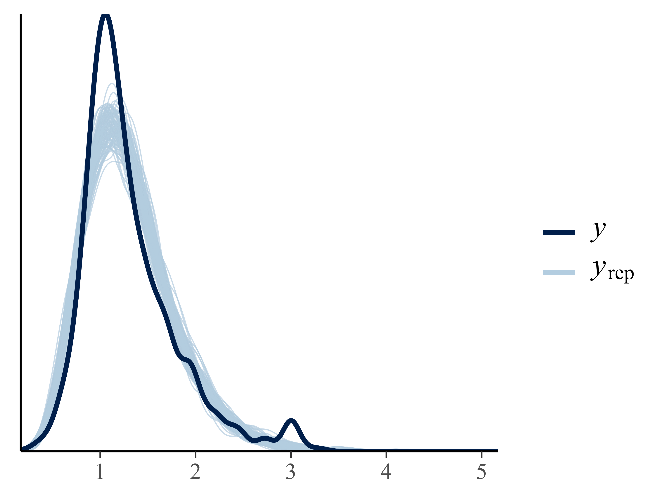


**Figure S36.** Posterior predictive check for generalized linear multilevel/mixed effect model of community mean body length regressed on first- and second-degree orthogonal polynomials of mean temperature difference (with elevational zone random effects) showing kernel density estimates for each of 100 draws from the posterior distribution (*y*_rep_) overlain with that of the response variable (*y*).

**
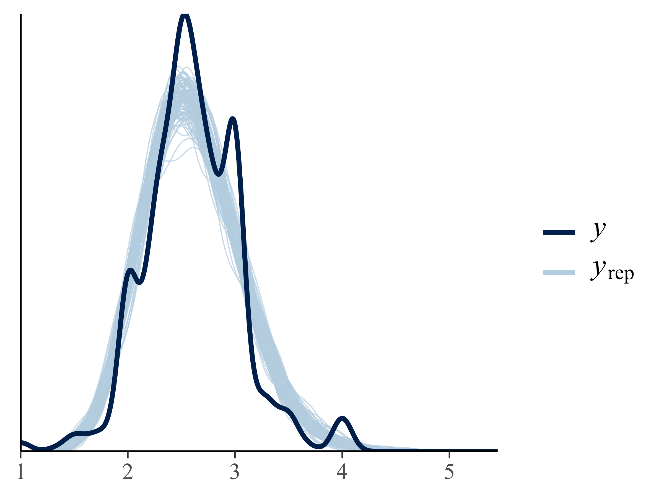
**

**Figure S37.** Posterior predictive check for generalized linear multilevel/mixed effect model of community mean feeding guilds regressed on first- and second-degree orthogonal polynomials of mean annual temperature (with latitudinal zone random effects) showing kernel density estimates for each of 100 draws from the posterior distribution (*y*_rep_) overlain with that of the response variable (*y*).


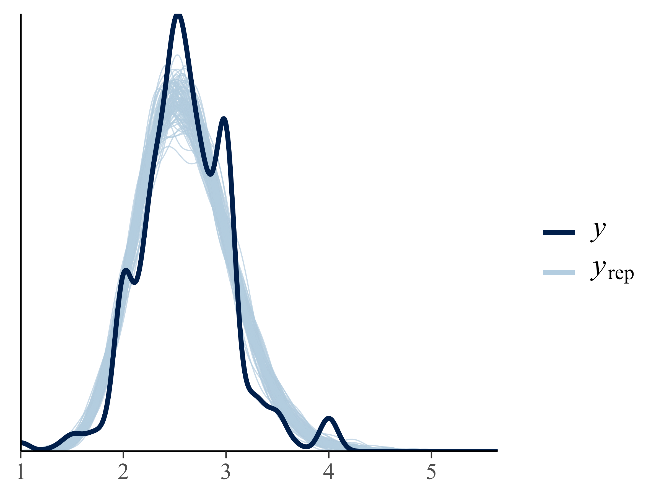


**Figure S38.** Posterior predictive check for generalized linear multilevel/mixed effect model of community mean feeding guilds regressed on first- and second-degree orthogonal polynomials of mean annual temperature (with elevational zone random effects) showing kernel density estimates for each of 100 draws from the posterior distribution (*y*_rep_) overlain with that of the response variable (*y*).

**
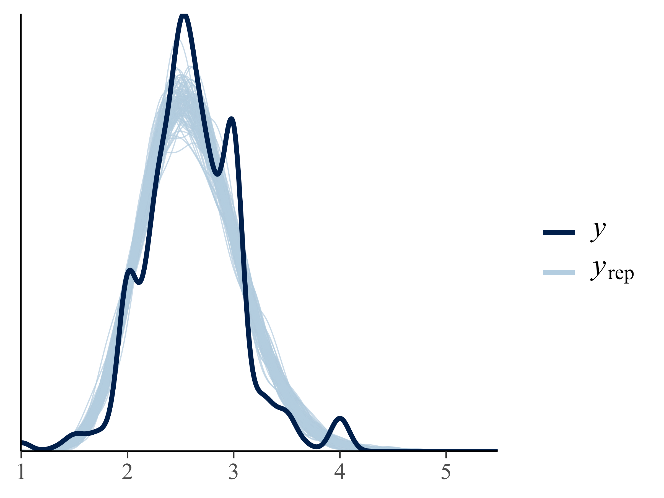
**

**Figure S39.** Posterior predictive check for generalized linear multilevel/mixed effect model of community mean feeding guilds regressed on first- and second-degree orthogonal polynomials of mean temperature difference (with latitudinal zone random effects) showing kernel density estimates for each of 100 draws from the posterior distribution (*y*_rep_) overlain with that of the response variable (*y*).


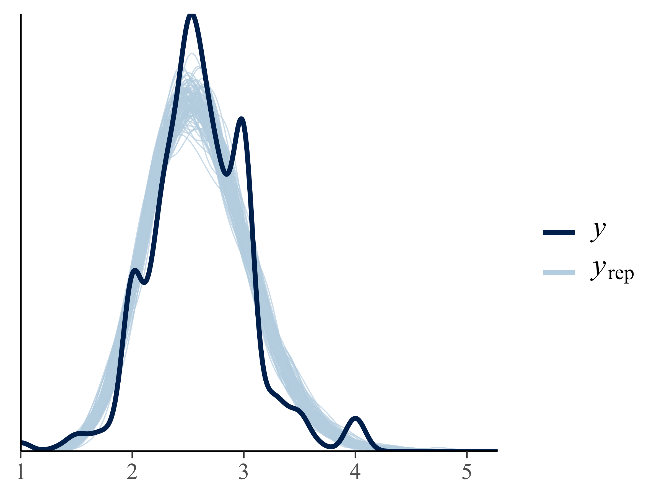


**Figure S40.** Posterior predictive check for generalized linear multilevel/mixed effect model of community mean feeding guilds regressed on first- and second-degree orthogonal polynomials of mean temperature difference (with elevational zone random effects) showing kernel density estimates for each of 100 draws from the posterior distribution (*y*_rep_) overlain with that of the response variable (*y*).

**
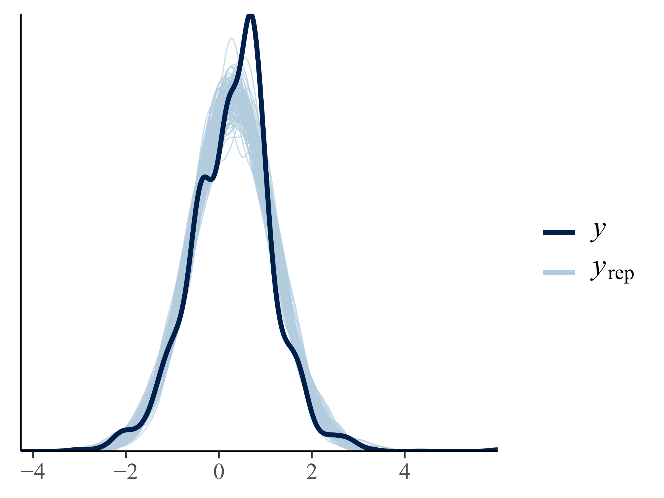
**

**Figure S41.** Posterior predictive check for generalized linear multilevel/mixed effect model of phylogenetic richness SES regressed on first- and second-degree orthogonal polynomials of mean annual temperature (with latitudinal zone random effects) showing kernel density estimates for each of 100 draws from the posterior distribution (*y*_rep_) overlain with that of the response variable (*y*).


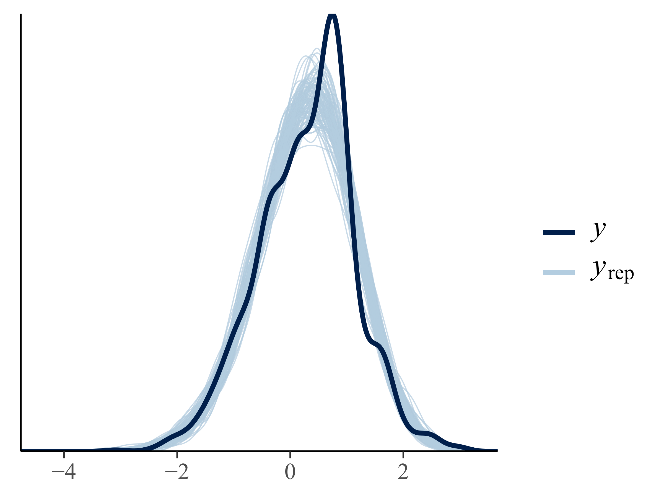


**Figure S42.** Posterior predictive check for generalized linear multilevel/mixed effect model of phylogenetic richness SES regressed on first- and second-degree orthogonal polynomials of mean annual temperature (with elevational zone random effects) showing kernel density estimates for each of 100 draws from the posterior distribution (*y*_rep_) overlain with that of the response variable (*y*).

**
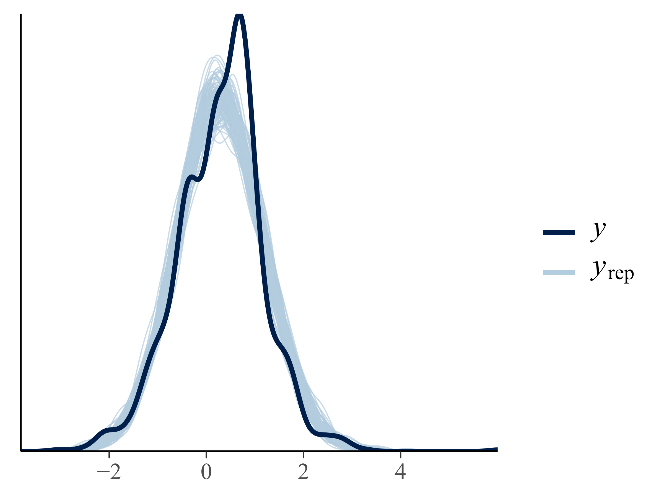
**

**Figure S43.** Posterior predictive check for generalized linear multilevel/mixed effect model of phylogenetic richness SES regressed on first- and second-degree orthogonal polynomials of mean temperature difference (with latitudinal zone random effects) showing kernel density estimates for each of 100 draws from the posterior distribution (*y*_rep_) overlain with that of the response variable (*y*).


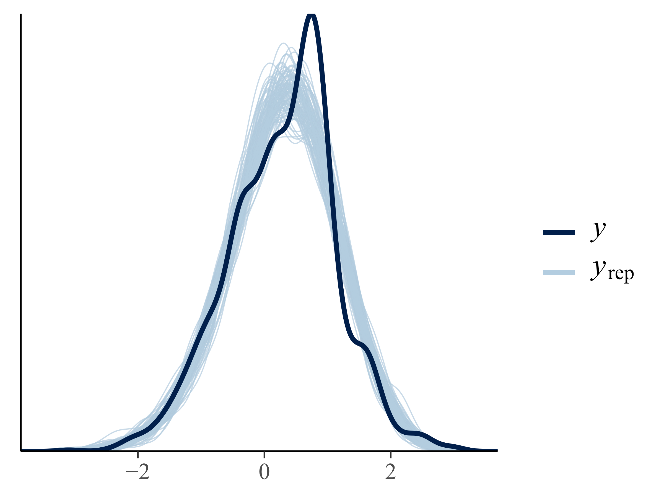


**Figure S44.** Posterior predictive check for generalized linear multilevel/mixed effect model of phylogenetic richness SES regressed on first- and second-degree orthogonal polynomials of mean temperature difference (with elevational zone random effects) showing kernel density estimates for each of 100 draws from the posterior distribution (*y*_rep_) overlain with that of the response variable (*y*).

**
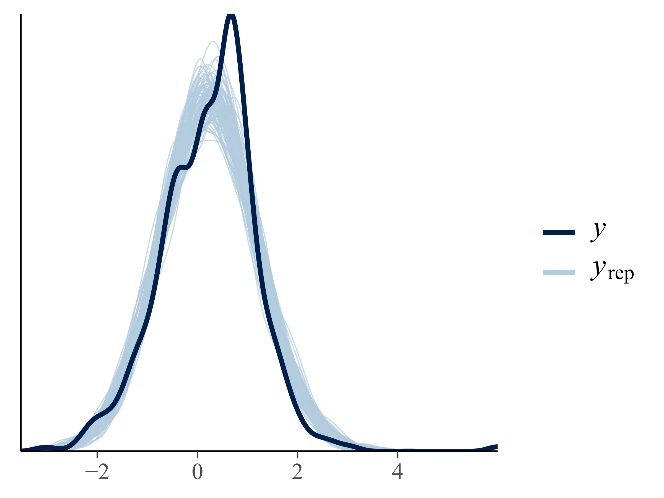
**

**Figure S45.** Posterior predictive check for generalized linear multilevel/mixed effect model of phylogenetic mean pairwise distance SES regressed on first- and second-degree orthogonal polynomials of mean annual temperature (with latitudinal zone random effects) showing kernel density estimates for each of 100 draws from the posterior distribution (*y*_rep_) overlain with that of the response variable (*y*).


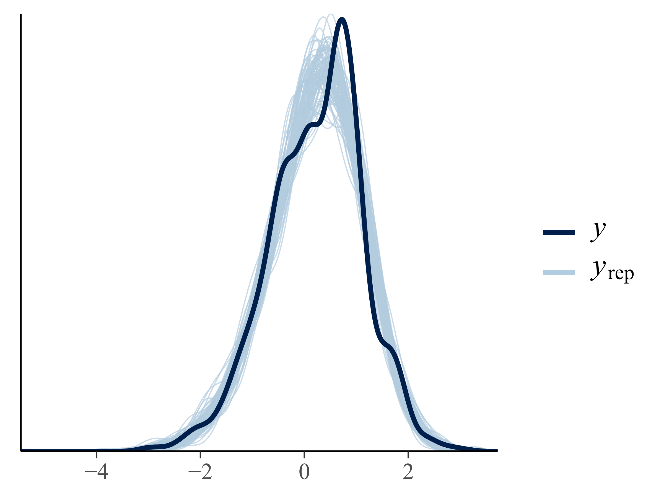


**Figure S46.** Posterior predictive check for generalized linear multilevel/mixed effect model of phylogenetic mean pairwise distance SES regressed on first- and second-degree orthogonal polynomials of mean annual temperature (with elevational zone random effects) showing kernel density estimates for each of 100 draws from the posterior distribution (*y*_rep_) overlain with that of the response variable (*y*).

**
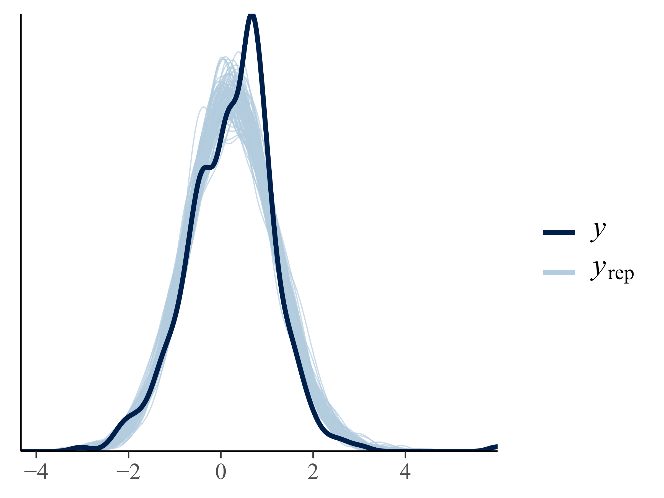
**

**Figure S47.** Posterior predictive check for generalized linear multilevel/mixed effect model of phylogenetic mean pairwise distance SES regressed on first- and second-degree orthogonal polynomials of mean temperature difference (with latitudinal zone random effects) showing kernel density estimates for each of 100 draws from the posterior distribution (*y*_rep_) overlain with that of the response variable (*y*).


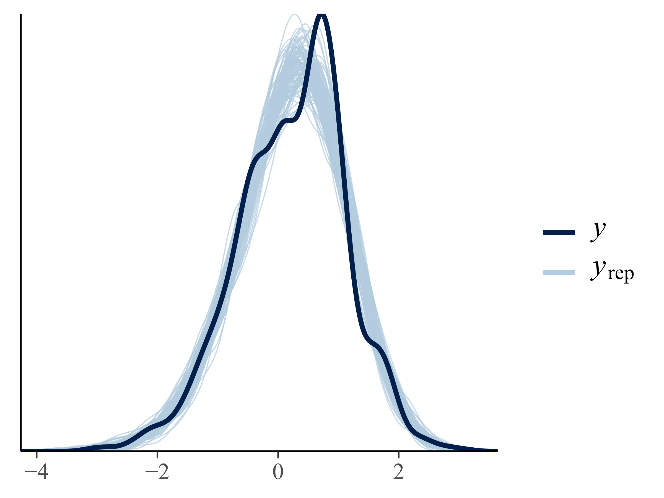


**Figure S48.** Posterior predictive check for generalized linear multilevel/mixed effect model of phylogenetic mean pairwise distance SES regressed on first- and second-degree orthogonal polynomials of mean temperature difference (with elevational zone random effects) showing kernel density estimates for each of 100 draws from the posterior distribution (*y*_rep_) overlain with that of the response variable (*y*).

**
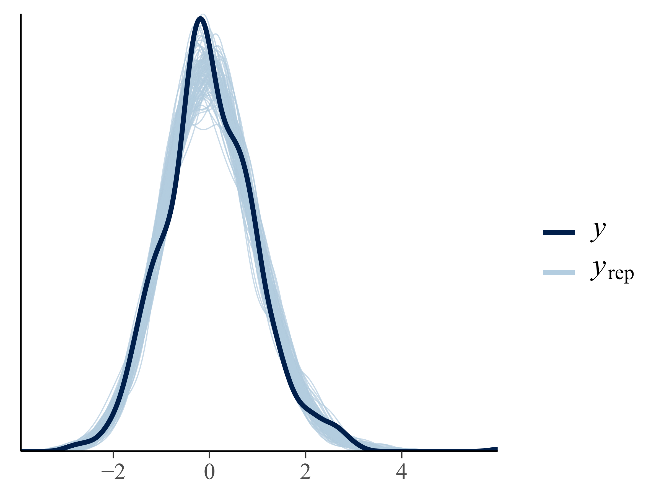
**

**Figure S49.** Posterior predictive check for generalized linear multilevel/mixed effect model of functional richness SES regressed on first- and second-degree orthogonal polynomials of mean annual temperature (with latitudinal zone random effects) showing kernel density estimates for each of 100 draws from the posterior distribution (*y*_rep_) overlain with that of the response variable (*y*).


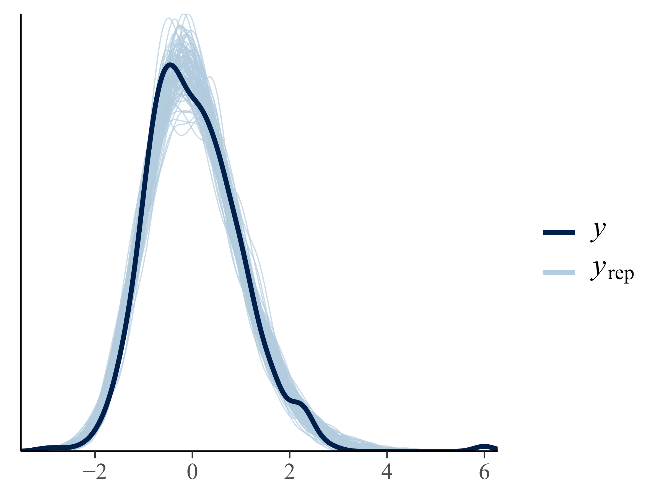


**Figure S50.** Posterior predictive check for generalized linear multilevel/mixed effect model of functional richness SES regressed on first- and second-degree orthogonal polynomials of mean annual temperature (with elevational zone random effects) showing kernel density estimates for each of 100 draws from the posterior distribution (*y*_rep_) overlain with that of the response variable (*y*).


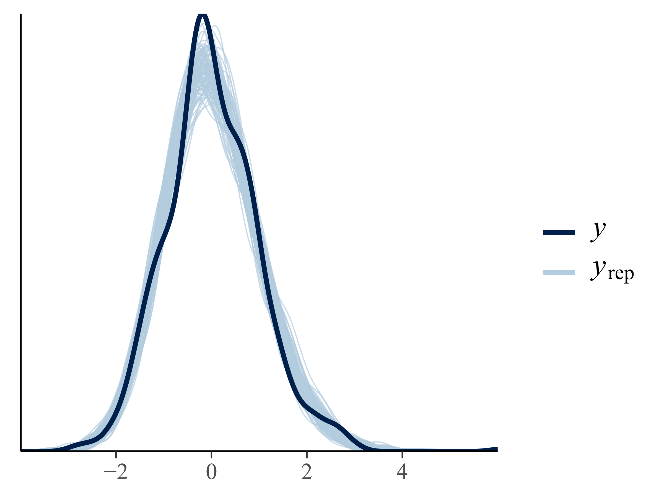


**Figure S51.** Posterior predictive check for generalized linear multilevel/mixed effect model of functional richness SES regressed on first- and second-degree orthogonal polynomials of mean temperature difference (with latitudinal zone random effects) showing kernel density estimates for each of 100 draws from the posterior distribution (*y*_rep_) overlain with that of the response variable (*y*).


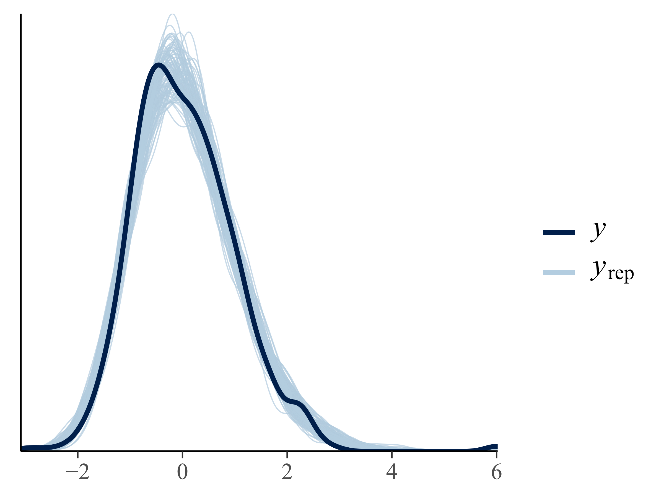


**Figure S52.** Posterior predictive check for generalized linear multilevel/mixed effect model of functional richness SES regressed on first- and second-degree orthogonal polynomials of mean temperature difference (with elevational zone random effects) showing kernel density estimates for each of 100 draws from the posterior distribution (*y*_rep_) overlain with that of the response variable (*y*).


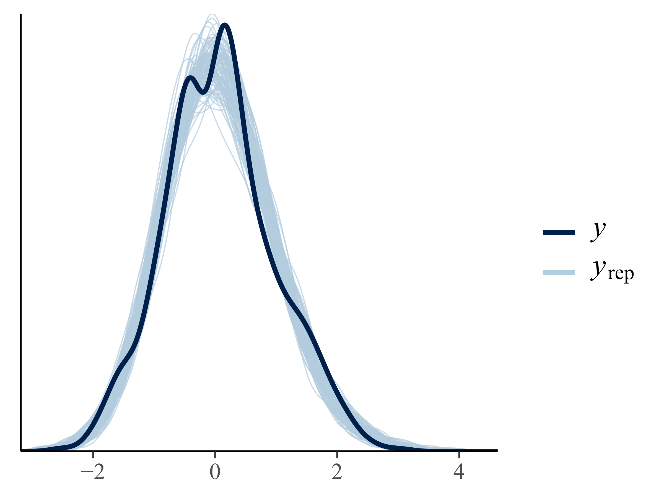


**Figure S53.** Posterior predictive check for generalized linear multilevel/mixed effect model of functional dispersion SES regressed on first- and second-degree orthogonal polynomials of mean annual temperature (with latitudinal zone random effects) showing kernel density estimates for each of 100 draws from the posterior distribution (*y*_rep_) overlain with that of the response variable (*y*).


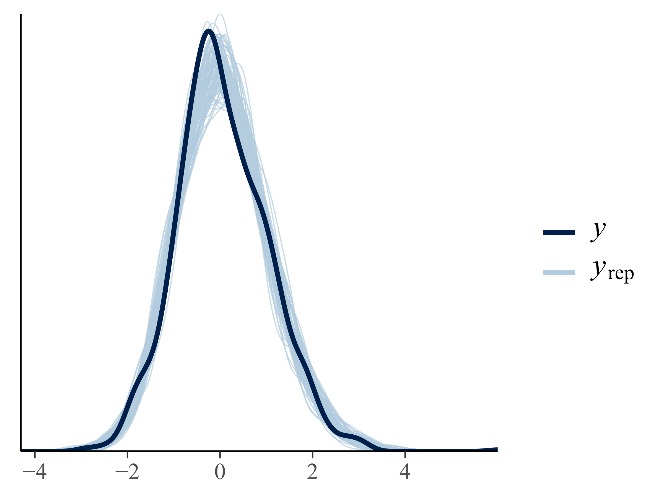


**Figure S54.** Posterior predictive check for generalized linear multilevel/mixed effect model of functional dispersion SES regressed on first- and second-degree orthogonal polynomials of mean annual temperature (with elevational zone random effects) showing kernel density estimates for each of 100 draws from the posterior distribution (*y*_rep_) overlain with that of the response variable (*y*).


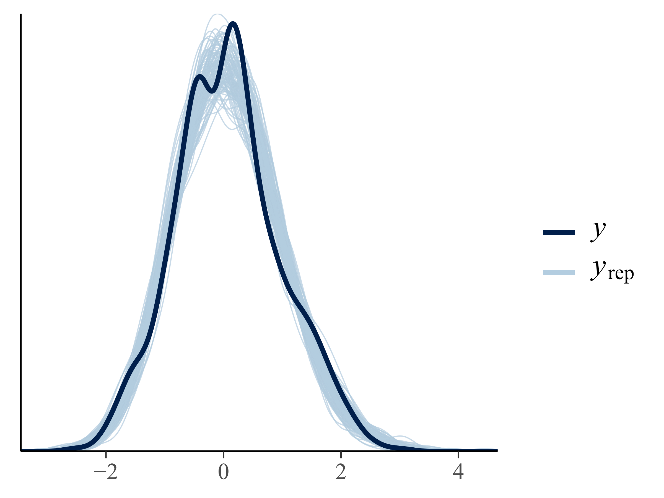


**Figure S55.** Posterior predictive check for generalized linear multilevel/mixed effect model of functional dispersion SES regressed on first- and second-degree orthogonal polynomials of mean temperature difference (with latitudinal zone random effects) showing kernel density estimates for each of 100 draws from the posterior distribution (*y*_rep_) overlain with that of the response variable (*y*).


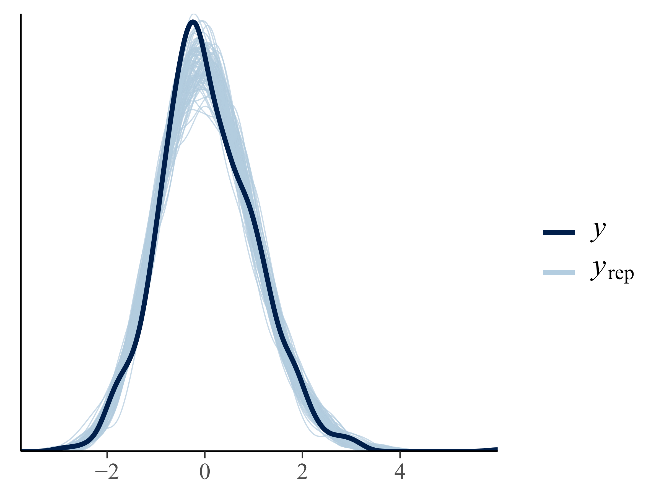


**Figure S56.** Posterior predictive check for generalized linear multilevel/mixed effect model of functional dispersion SES regressed on first- and second-degree orthogonal polynomials of mean temperature difference (with elevational zone random effects) showing kernel density estimates for each of 100 draws from the posterior distribution (*y*_rep_) overlain with that of the response variable (*y*).


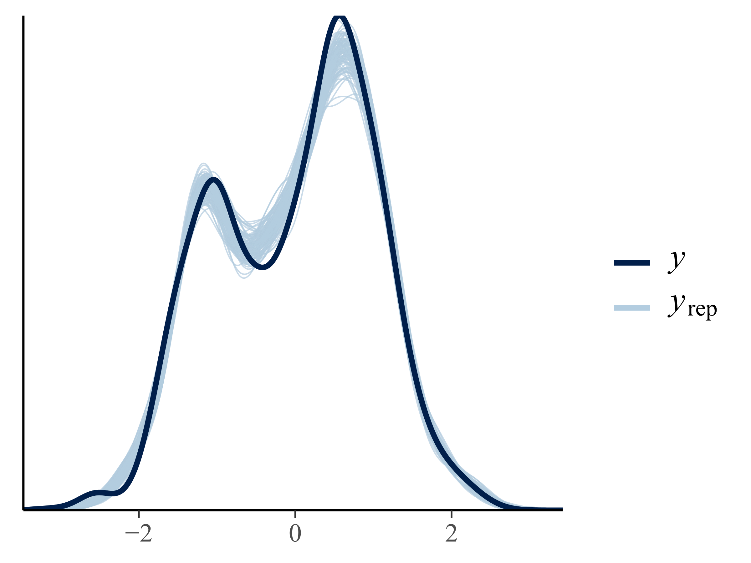


**Figure S57.** Posterior predictive check for generalized linear multilevel/mixed effect model of mean annual temperature regressed on first- and second-degree orthogonal polynomials of elevation (with latitudinal zone random effects) showing kernel density estimates for each of 100 draws from the posterior distribution (*y*_rep_) overlain with that of the response variable (*y*).


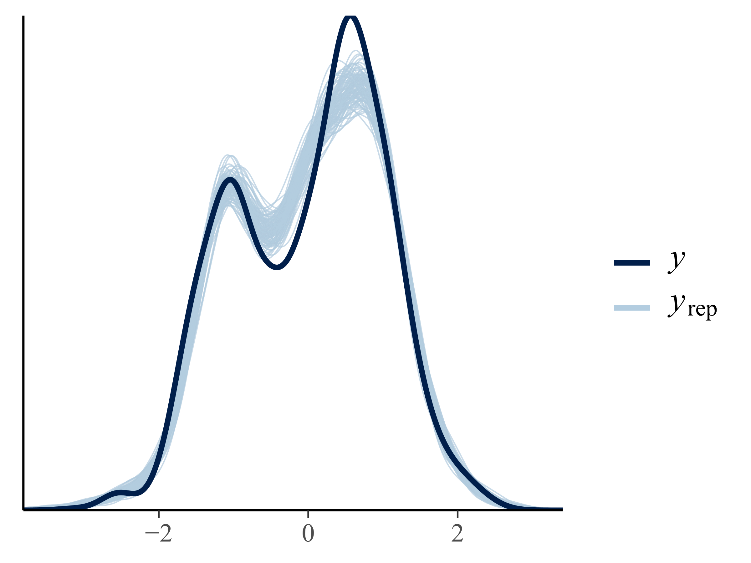


**Figure S58.** Posterior predictive check for generalized linear multilevel/mixed effect model of mean annual temperature regressed on first- and second-degree orthogonal polynomials of latitude (with elevational zone random effects) showing kernel density estimates for each of 100 draws from the posterior distribution (*y*_rep_) overlain with that of the response variable (*y*).


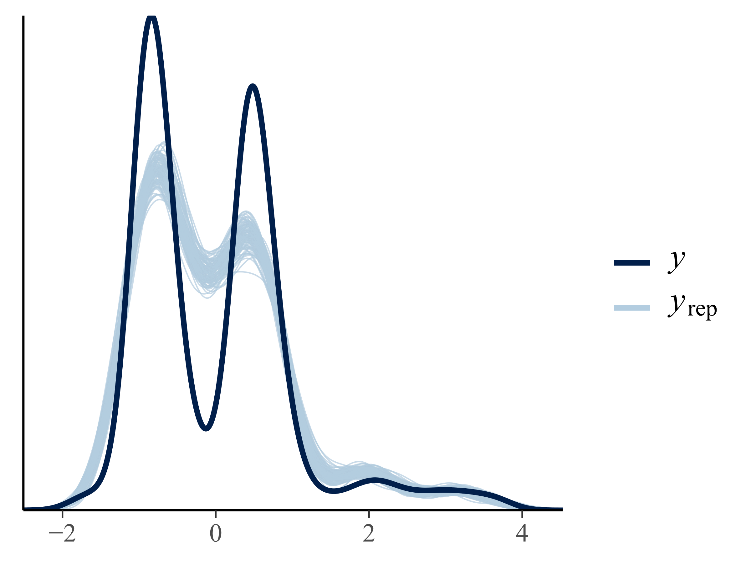


**Figure S59.** Posterior predictive check for generalized linear multilevel/mixed effect model of mean temperature difference regressed on first- and second-degree orthogonal polynomials of elevation (with latitudinal zone random effects) showing kernel density estimates for each of 100 draws from the posterior distribution (*y*_rep_) overlain with that of the response variable (*y*).


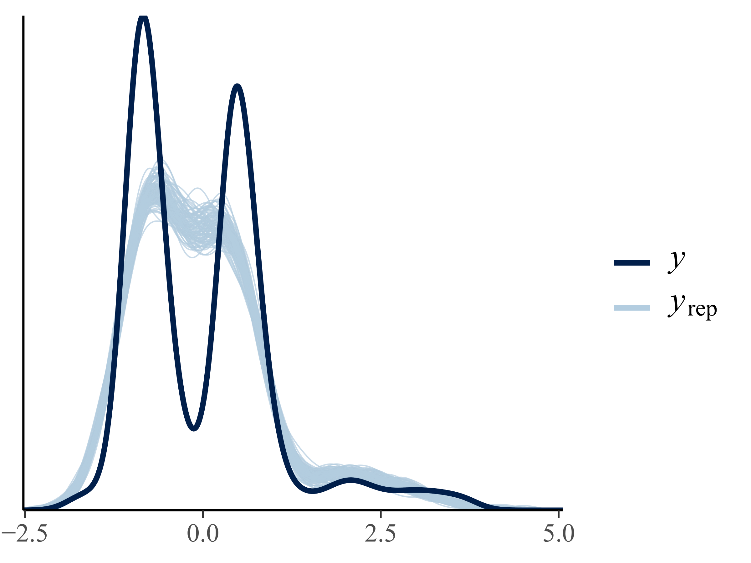


**Figure S60.** Posterior predictive check for generalized linear multilevel/mixed effect model of mean temperature difference regressed on first- and second-degree orthogonal polynomials of latitude (with elevational zone random effects) showing kernel density estimates for each of 100 draws from the posterior distribution (*y*_rep_) overlain with that of the response variable (*y*).


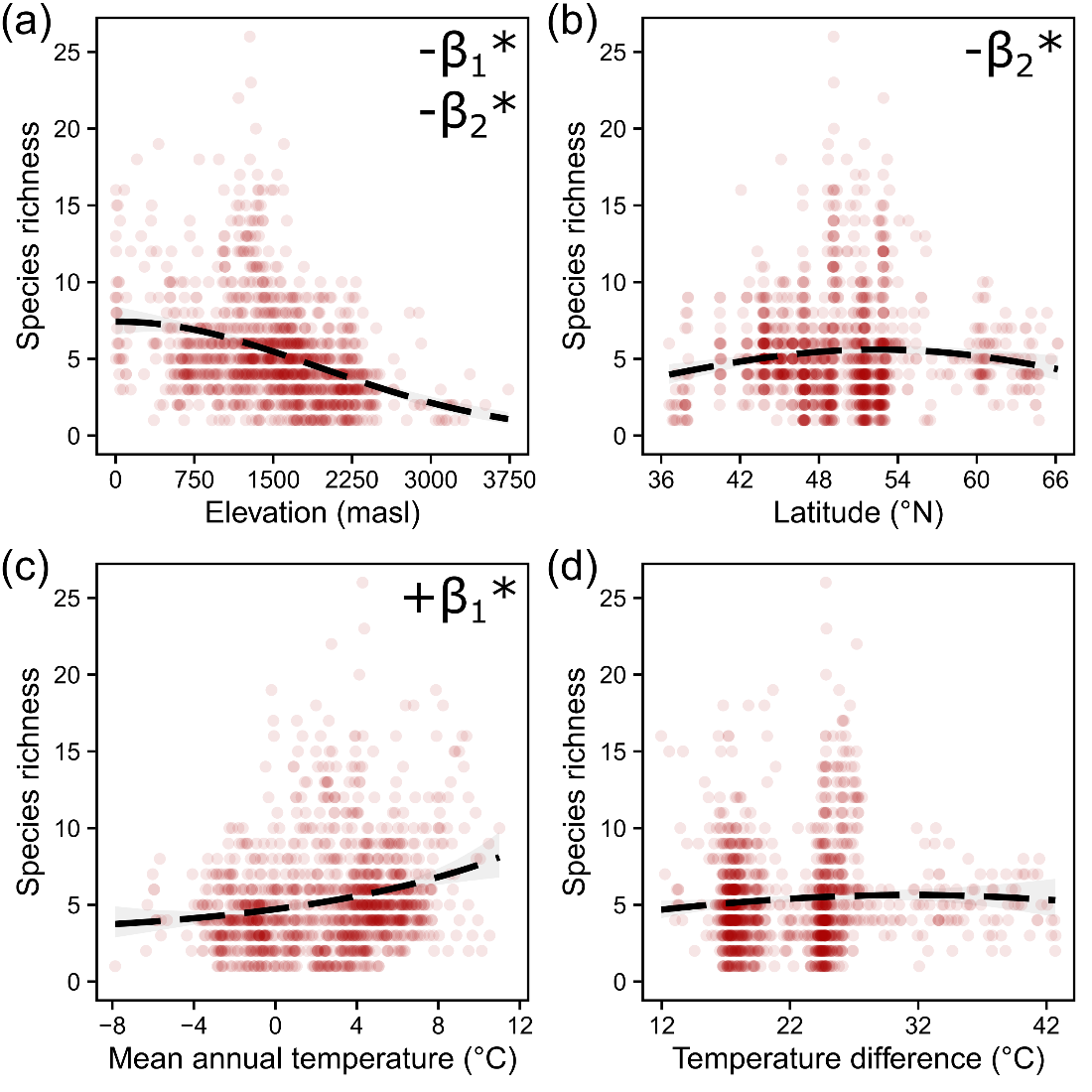


**Figure S61.**  Predictions estimated from posterior draws of the linear predictor for generalized linear (negative binomial) fixed effect models of species richness regressed on first- and second-degree orthogonal polynomials of elevation (a), latitude (b), mean annual temperature (c), and temperature difference between the means of the warmest and coldest months (d). Mean slope coefficients with 95% probability of being either positive or negative are noted in the top right corners of each plot for steepness (β_1_) and curvature (β_2_). Shaded regions are 95% prediction intervals and points are values at individual sampling locations. Note these predictions do not account for random effects (latitudinal or elevational zones presented in Figure 3 of the main article) and thus should be considered ‘naïve’ analyses that ignore potential interactions between elevation and latitude.

**
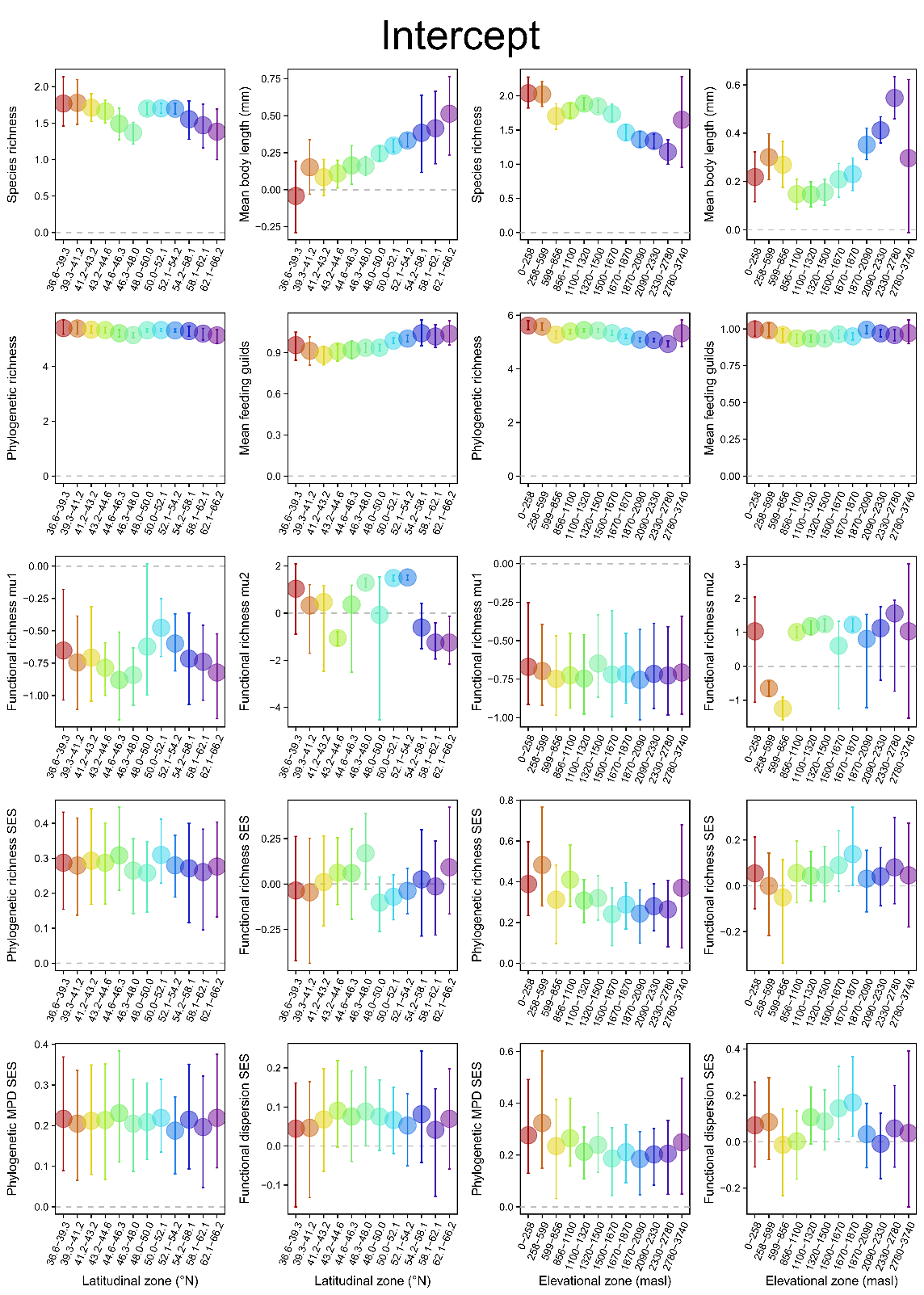
**

**Figure S62.** Mean group-level intercepts for generalized linear multilevel/mixed effect models of each biodiversity metric regressed on first- and second-degree orthogonal polynomials of elevation and latitude. Intercepts for functional richness are from mixture models and presented for each component separately. Error bars show 95% credible intervals. Colours correspond to elevational and latitudinal zones presented in Figure 1.

**
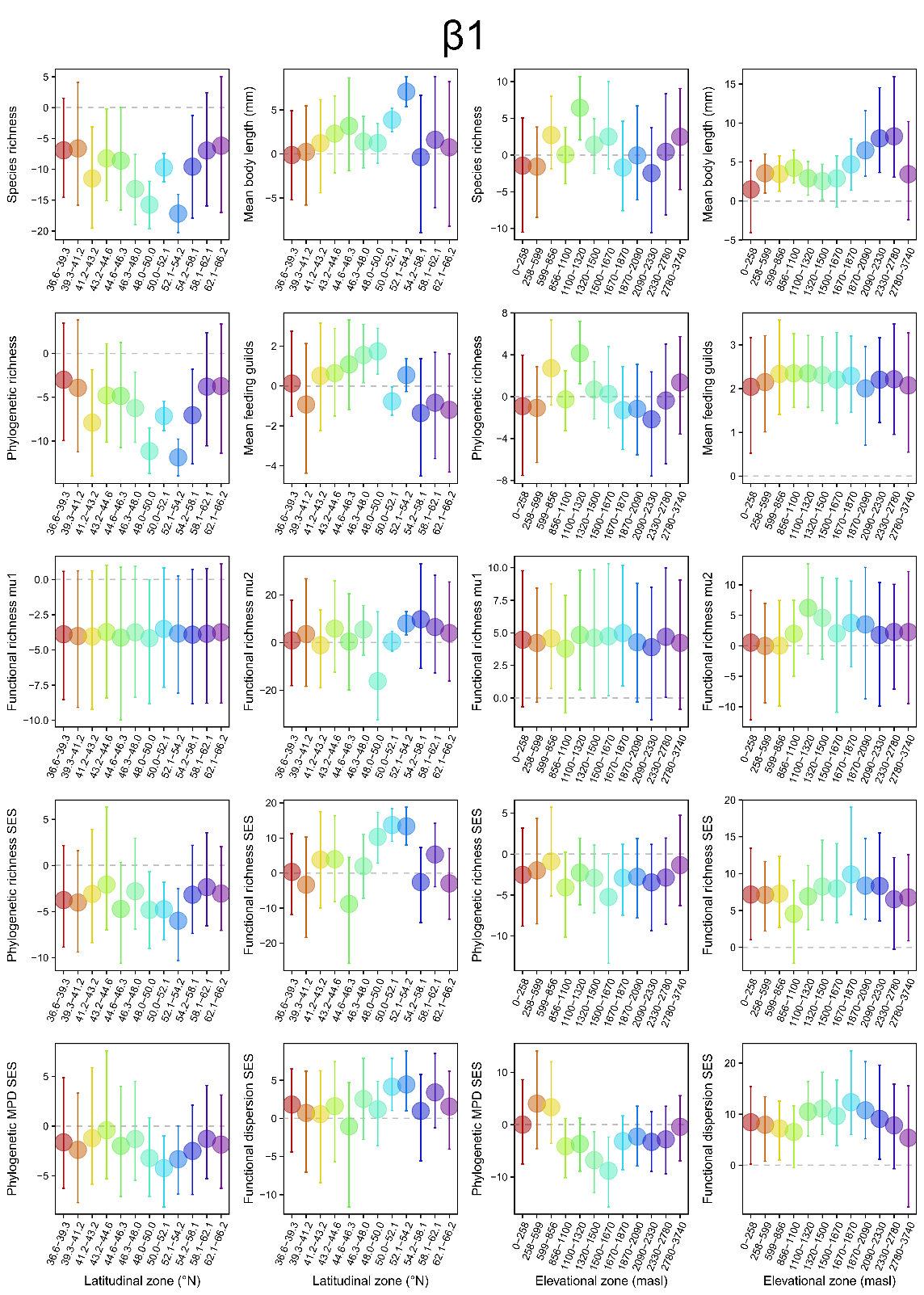
**

**Figure S63.** Mean group-level β_1_ coefficients for generalized linear multilevel/mixed effect models of each biodiversity metric regressed on first- and second-degree orthogonal polynomials of elevation and latitude. Coefficients for functional richness are from mixture models and presented for each component separately. Error bars show 95% credible intervals. Colours correspond to elevational and latitudinal zones presented in Figure 1.


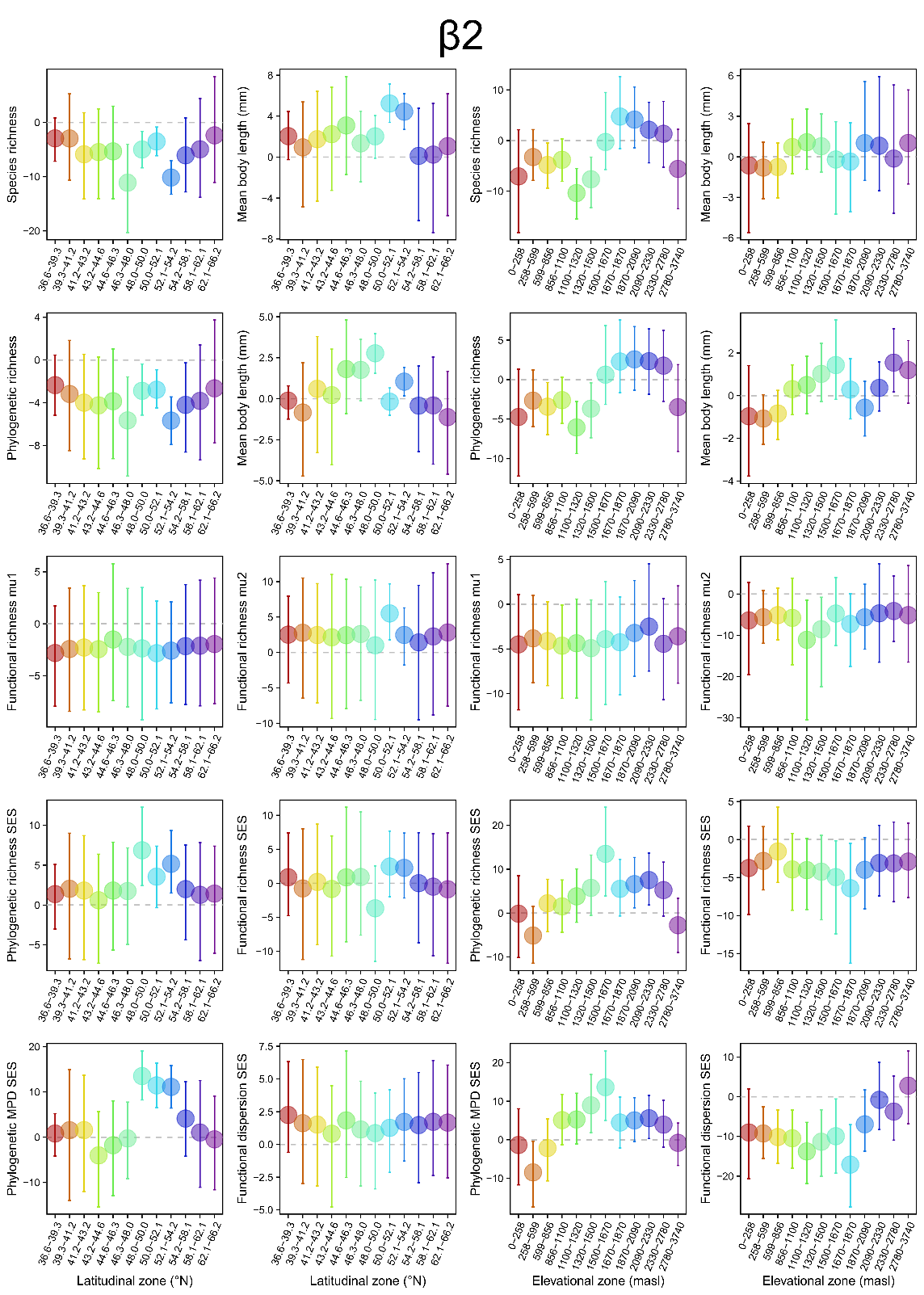


**Figure S64.** Mean group-level β_2_ coefficients for generalized linear multilevel/mixed effect models of each biodiversity metric regressed on first- and second-degree orthogonal polynomials of elevation and latitude. Coefficients for functional richness are from mixture models and presented for each component separately. Error bars show 95% credible intervals. Colours correspond to elevational and latitudinal zones presented in Figure 1.

**
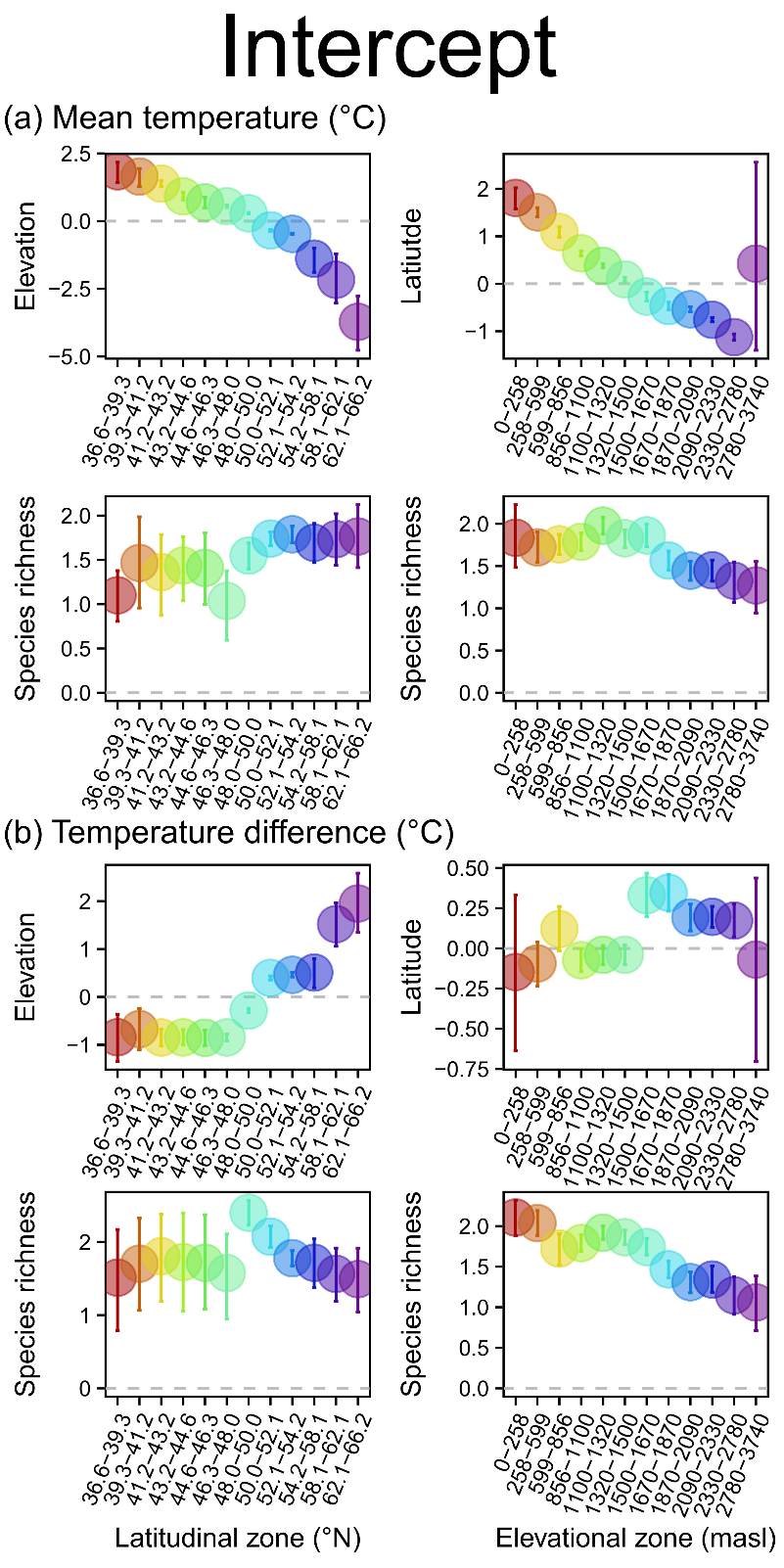
**

**Figure S65.** Mean group-level intercepts for generalized linear multilevel/mixed effect models of species richness, elevation, and latitude regressed on first- and second-degree orthogonal polynomials of (a) mean annual temperature and (b) temperature difference between the means of the warmest and coldest months. Error bars show 95% credible intervals. Colours correspond to elevational and latitudinal zones presented in Figure 1.

**
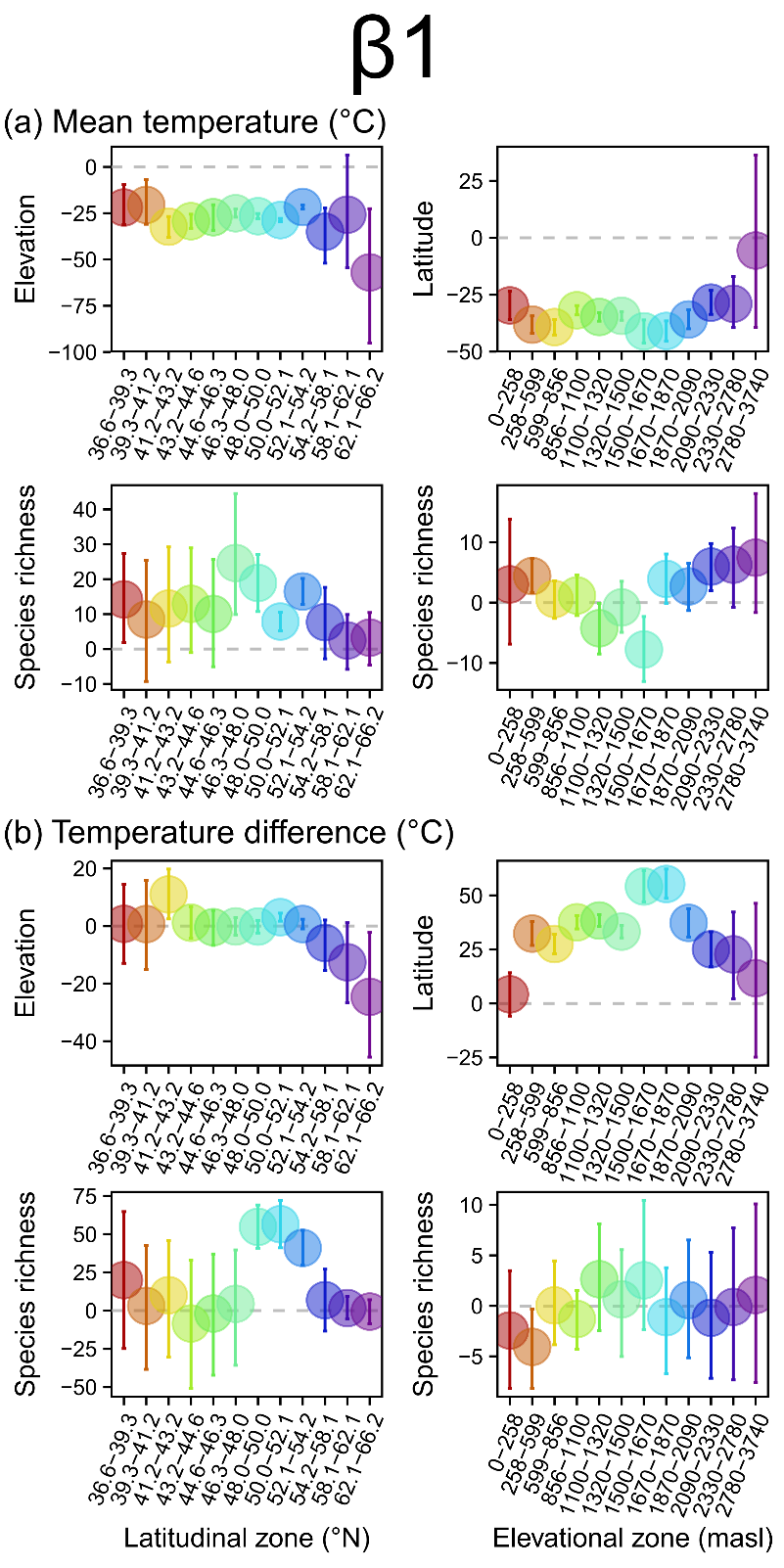
**

**Figure S66.** Mean group-level β_1_ coefficients for generalized linear multilevel/mixed effect models of species richness, elevation, and latitude regressed on first- and second-degree orthogonal polynomials of (a) mean annual temperature and (b) temperature difference between the means of the warmest and coldest months. Error bars show 95% credible intervals. Colours correspond to elevational and latitudinal zones presented in Figure 1.


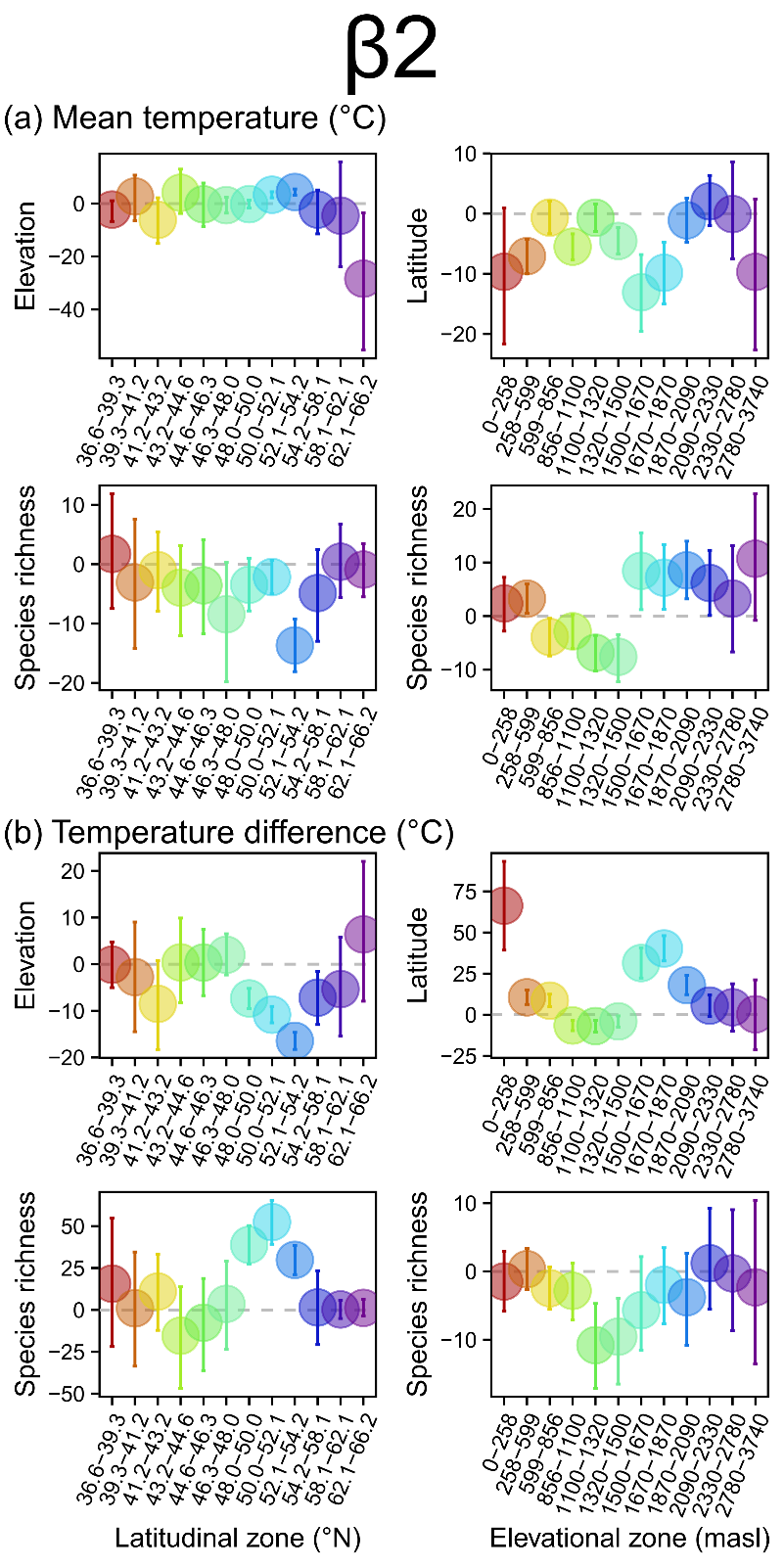


**Figure S67.** Mean group-level β_2_ coefficients for generalized linear multilevel/mixed effect models of species richness, elevation, and latitude regressed on first- and second-degree orthogonal polynomials of (a) mean annual temperature and (b) temperature difference between the means of the warmest and coldest months. Error bars show 95% credible intervals. Colours correspond to elevational and latitudinal zones presented in Figure 1.


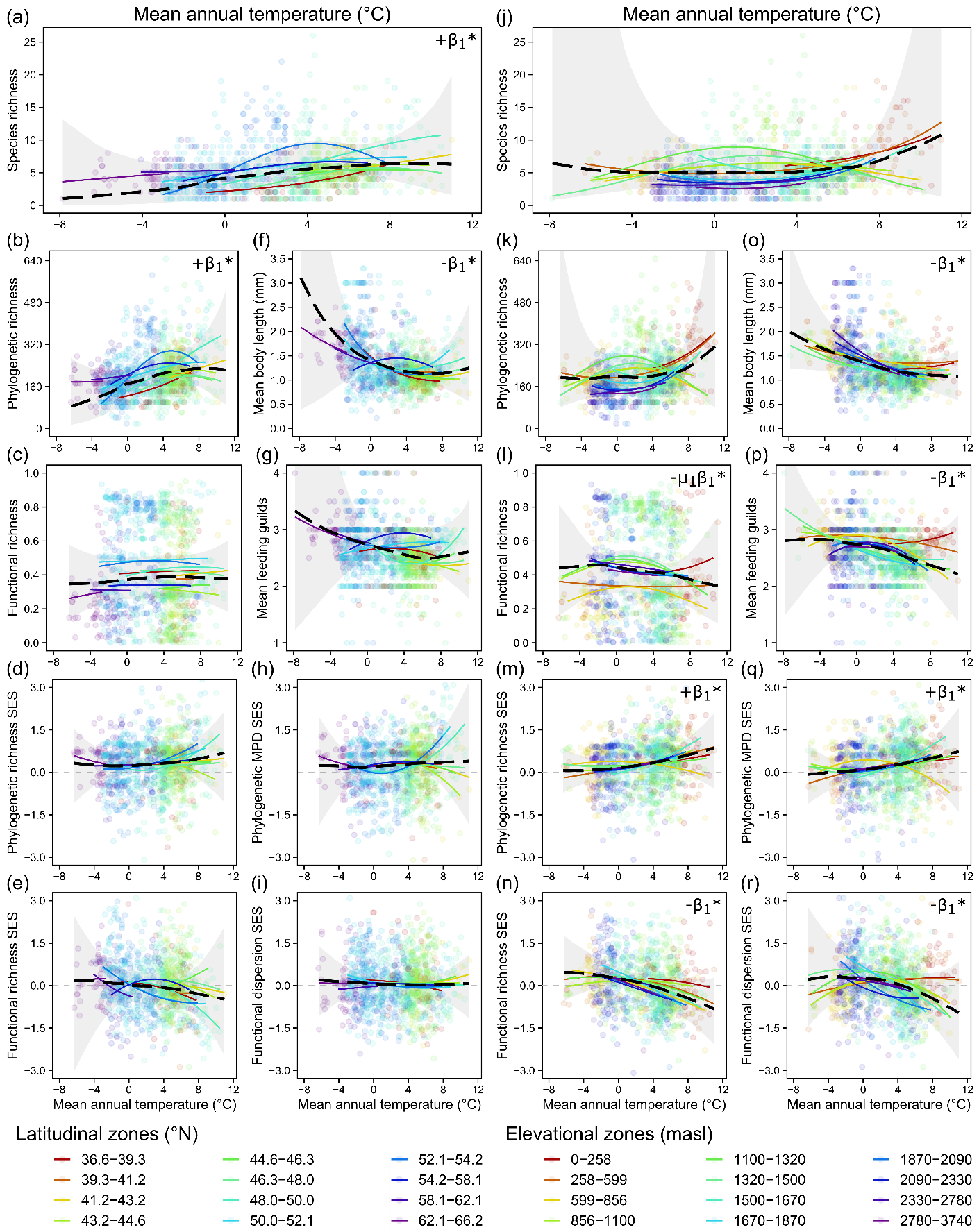


**Figure S68.** Population- and group-level predictions (representing overall and zone-specific trends presented as black dashed and coloured lines, respectively) estimated from posterior draws of the linear predictor for generalized linear multilevel/mixed effects models of each biodiversity metric regressed on first- and second-degree orthogonal polynomials of mean annual temperature. Geographic predictors were centered and scaled (as *z*-scores). Random effects were specified as latitudinal zones (a–i) emphasizing elevation-driven change and elevational zones (j–r) emphasizing latitude-driven change. Mean population-level slope coefficients with 95% probability of being either positive or negative are included in the top right corners of each plot for steepness (β_1_) and curvature (β_2_). Parameters for functional richness are from mixture models and assessed for each component separately (μ_1_ and μ_2_). Group-level predictions are shown only for their distributional bounds. Shaded regions are population-level 95% prediction intervals and points are individual sampling locations. Grey dashed horizontal lines on plots for standardized metrics indicate no difference from the null expectation. SES denotes standardized effect sizes and MPD denotes mean pairwise distance. See Table S5 for detailed results.


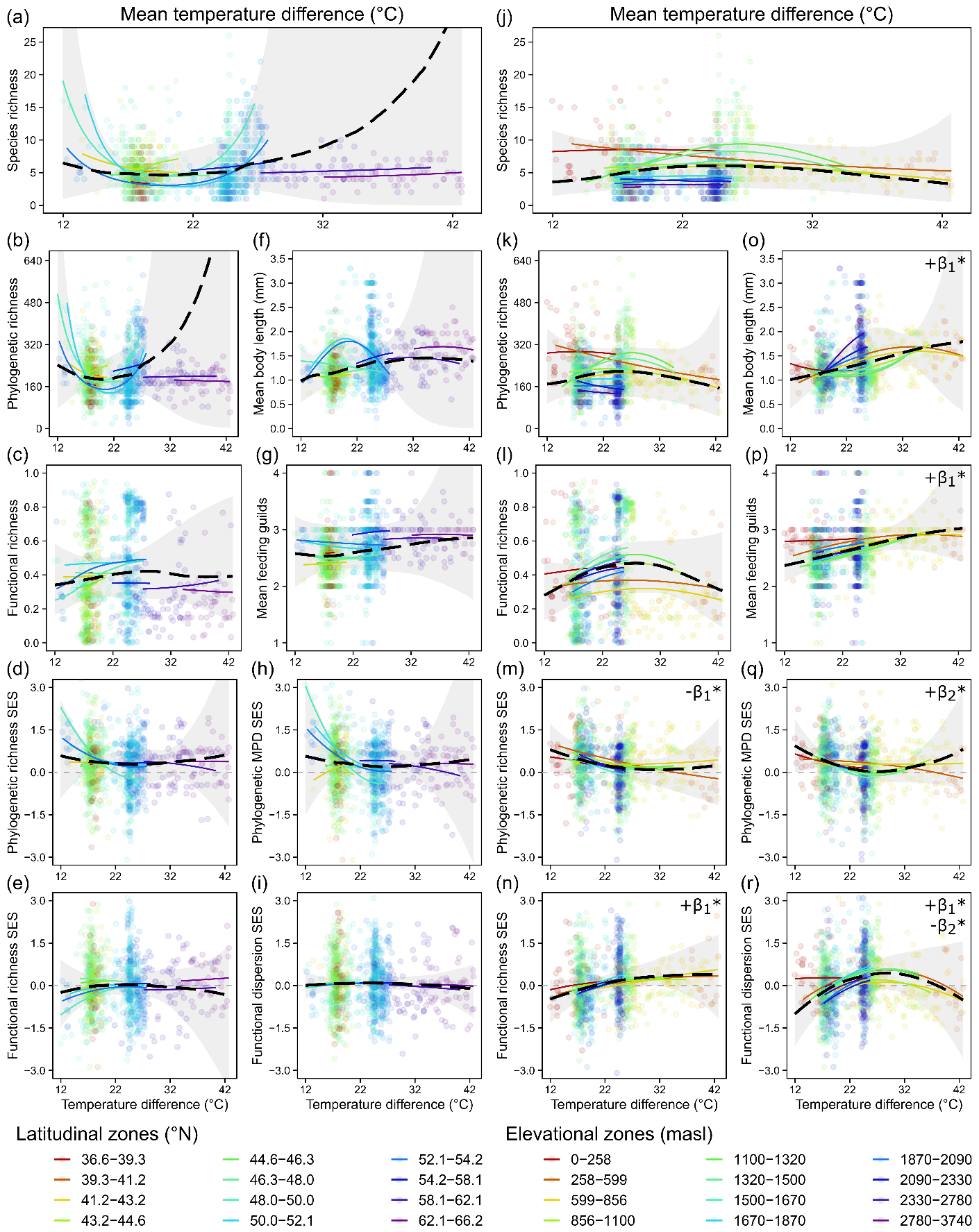


**Figure S69.** Population- and group-level predictions (representing overall and zone-specific trends presented as black dashed and coloured lines, respectively) estimated from posterior draws of the linear predictor for generalized linear multilevel/mixed effects models of each biodiversity metric regressed on first- and second-degree orthogonal polynomials of mean temperature difference. Geographic predictors were centered and scaled (as *z*-scores). Random effects were specified as latitudinal zones (a–i) emphasizing elevation-driven change and elevational zones (j–r) emphasizing latitude-driven change. Mean population-level slope coefficients with 95% probability of being either positive or negative are included in the top right corners of each plot for steepness (β_1_) and curvature (β_2_). Parameters for functional richness are from mixture models and assessed for each component separately (μ_1_ and μ_2_). Group-level predictions are shown only for their distributional bounds. Shaded regions are population-level 95% prediction intervals and points are individual sampling locations. Grey dashed horizontal lines on plots for standardized metrics indicate no difference from the null expectation. SES denotes standardized effect sizes and MPD denotes mean pairwise distance. See Table S5 for detailed results.


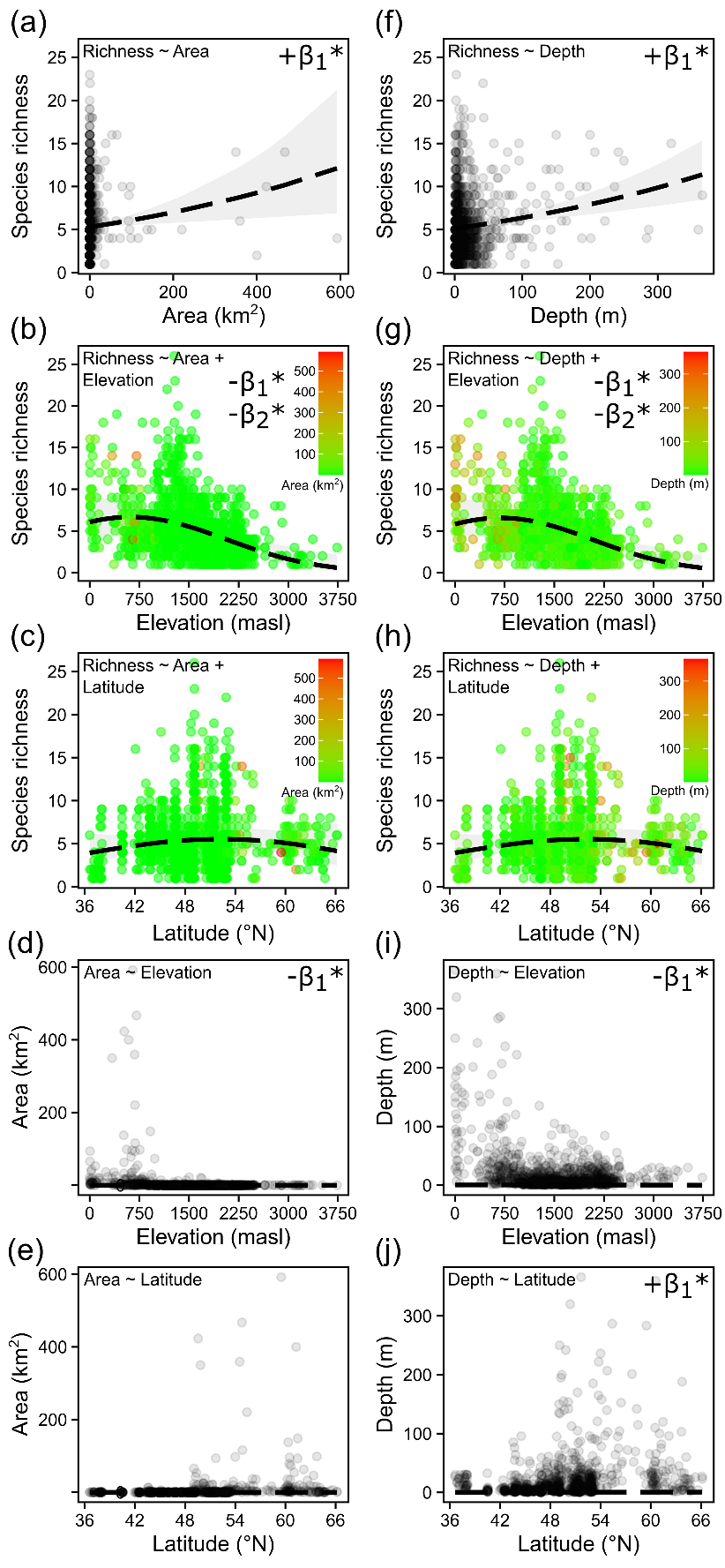


**Figure S70.** Population-level predictions (overall trends presented as black dashed lines) estimated from posterior draws of the linear predictor for generalized linear multilevel/mixed effects models evaluating relationships with waterbody area (a–e) and depth (f–j). Panels (a) and (f) present simple negative binomial regression of species richness regressed on area and depth, respectively. Panel (b) presents negative binomial regression of species richness regressed on area and first- and second-degree orthogonal polynomials of elevation (with latitudinal zone random effects). Panel (c) presents negative binomial regression of species richness regressed on area and first- and second-degree orthogonal polynomials of latitude (with elevational zone random effects). Panel (g) presents negative binomial regression of species richness regressed on depth and first- and second-degree orthogonal polynomials of elevational (with latitudinal zone random effects). Panel (h) presents negative binomial regression of species richness regressed on depth and first- and second-degree orthogonal polynomials of latitude (with elevational zone random effects). Panels (d–e) present simple skew-normal regression of area regressed on elevation and latitude, respectively. Panels (i–j) present simple skew-normal regression of depth regressed on elevation and latitude, respectively. All predictors were centered and scaled (as *z*-scores). Population-level slope coefficients with 95% probability of being either positive or negative are included in the top right corners of each plot (β_1_ and β_2_). Shaded regions are population-level 95% prediction intervals and points are individual sampling locations. See Table S7 for detailed results.


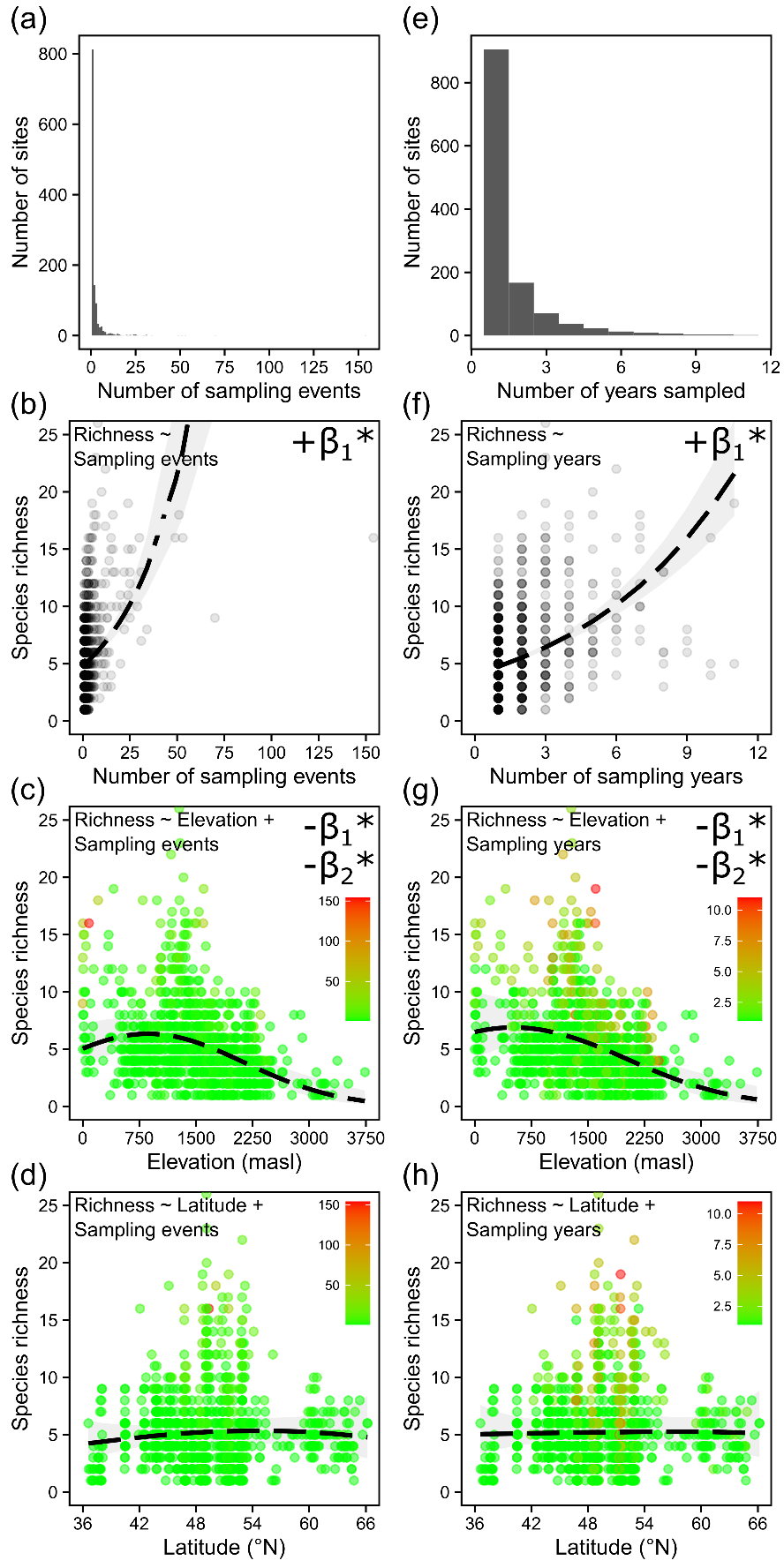


**Figure S71.** Histograms presenting number of sampling events (a) and years sampled (e) at each site and plots of population-level predictions (overall trends presented as black dashed lines) estimated from posterior draws of the linear predictor for generalized linear multilevel/mixed effects models (negative binomial regression) evaluating species richness regressed on elevation, latitude, number of sampling events (b–d) and years sampled (f–h). Panels (c) and (g) present species richness regressed on first- and second-degree orthogonal polynomials of elevation (with latitudinal zone random effects). Panel (d) and (h) present species richness regressed on first- and second-degree orthogonal polynomials of latitude (with elevational zone random effects). All predictors were centered and scaled (as *z*-scores). Mean population-level slope coefficients with 95% probability of being either positive or negative are included in the top right corners of each plot (β_1_ and β_2_). Shaded regions are population-level 95% prediction intervals and points are individual sampling locations. See Table S8 for detailed results.
